# MyoD induced enhancer RNA interacts with hnRNPL to activate target gene transcription during myogenic differentiation

**DOI:** 10.1038/s41467-019-13598-0

**Published:** 2019-12-19

**Authors:** Yu Zhao, Jiajian Zhou, Liangqiang He, Yuying Li, Jie Yuan, Kun Sun, Xiaona Chen, Xichen Bao, Miguel A. Esteban, Hao Sun, Huating Wang

**Affiliations:** 1 Department of Orthopaedics and Traumatology, Li Ka Shing Institute of Health Sciences, The Chinese University of Hong Kong, Hong Kong, China; 2 Department of Chemical Pathology, Li Ka Shing Institute of Health Sciences, The Chinese University of Hong Kong, Hong Kong, China; 3 Laboratory of RNA Molecular Biology, Guangzhou Institutes of Biomedicine and Health, Chinese Academy of Sciences, Guangzhou, 510530 China; 4 Laboratory of Chromatin and Human Disease, Key Laboratory of Regenerative Biology, South China Institute for Stem Cell Biology and Regenerative Medicine, Guangzhou Institutes of Biomedicine and Health, Chinese Academy of Sciences, Guangzhou, China; 5 Present Address: Dermatology Hospital, Southern Medical University, Guangzhou, China; 6 Present Address: Institute of Cancer Research, Shenzhen Bay Laboratory, Shenzhen, 518000 China

**Keywords:** Differentiation, Non-coding RNAs, Transcription

## Abstract

Emerging evidence supports roles of enhancer RNAs (eRNAs) in regulating target gene. Here, we study eRNA regulation and function during skeletal myoblast differentiation. We provide a panoramic view of enhancer transcription and categorization of eRNAs. Master transcription factor MyoD is crucial in activating eRNA production. Super enhancer (se) generated *seRNA-1* and *-2* promote myogenic differentiation in vitro and in vivo. *seRNA-1* regulates expression levels of two nearby genes, myoglobin (*Mb*) and apolipoprotein L6 (*Apol6*), by binding to heterogeneous nuclear ribonucleoprotein L (hnRNPL). A CAAA tract on *seRNA-1* is essential in mediating *seRNA-1*/hnRNPL binding and function. Disruption of *seRNA-1*-hnRNPL interaction attenuates Pol II and H3K36me3 deposition at the *Mb* locus, in coincidence with the reduction of its transcription. Furthermore, analyses of hnRNPL binding transcriptome-wide reveal its association with eRNAs is a general phenomenon in multiple cells. Collectively, we propose that eRNA-hnRNPL interaction represents a mechanism contributing to target mRNA activation.

## Abstract (web summary)

Enhancers are often transcribed into non-coding RNAs called enhancer RNAs. Here the authors show that super enhancer generated *seRNA-1* regulates the expression of nearby myoglobin (*Mb*) gene by binding to heterogeneous nuclear ribonucleoprotein L during myogenic differentiation.

## Introduction

Cell-type specific transcriptional programs are generally dictated by a class of cis-acting regulatory elements known as enhancers. Transcription factors (TFs) bind these enhancer elements via embedded recognition sequences, recruit and cooperate with cofactors to modulate the target gene transcription through chromatin looping between enhancers and promoters^1^. Clusters of enhancers, termed super-enhancers (SEs) or stretch enhancers, are thought to play especially prominent role in determining cell identity^2,3^. Compared to typical enhancers (TEs), SEs encompass larger open chromatin domains demarcated with acetylation of histone H3 at lysine 27 (H3K27ac) and enriched for master TFs binding. Remodeling of TE/SE landscape often occurs in response to developmental and/or environmental stimuli, accounting for the ensuing transcriptomic change^4,5^. Our recent work consolidates this notion in the process of skeletal myoblast (MB) differentiation^6^. This process involves MBs differentiation into myotubes (MTs) and is a critical step in skeletal muscle formation during muscle development and injury induced regeneration process^7^. In addition, we have solidified a pivotal role of myogenic differentiation protein (*MyoD*) in enhancer/SEs assembly and activation^6^. In addition to serving as binding hubs for key TFs, it is widely accepted that enhancers are also prevalently bound by RNA polymerase II (Pol II), generating bi-directional non-coding RNAs dubbed enhancer RNAs (eRNAs)^8–12^. However, current knowledge about their functions and mechanistic roles is limited.

Emerging evidence also suggests eRNAs are integral components of enhancer function. In most situations, eRNAs act *in cis* to stimulate transcription of target mRNAs which are neighboring to or reside in the same topologically associating domain (TAD) with the eRNA loci^9–11,13^. Mechanistically, Lai et al. and Li et al. demonstrated that eRNAs can establish and/or stabilize chromatin looping between enhancers and promoters through interacting with components of mediator or cohesin complex^10,14^. Similarly, a recent study revealed eRNA expressed from a distal enhancer of *MyoD1* (^DRR^eRNA) activates *Myogenin* expression *in trans* through interacting with cohesin complex^15^. In a separate study, eRNAs are also directly involved in transcription process by acting as decoy for negative elongation factor (NELF) to promote the release of paused Pol II into productive elongation stage^16^. Zhao et al. later also showed that eRNAs may directly interact with component of positive transcription elongation factor b (P-TEFB) to control transcription elongation^17^. More recently, eRNAs, or nascent RNAs in a broader sense, were shown to trap the transcription factor YY1 and increase its local concentration at DNA^18^. Lastly, eRNAs also interact with transcriptional co-activator CREB binding protein (CBP) in a sequence independent manner to stimulate core histone acetyltransferase activity, thereby promoting gene expression^19^. Despite these substantial advances in our understanding of eRNAs, the investigation of mechanistic roles in their host enhancers remains largely incomplete, warranting the efforts in searching for additional protein binding partners and uncharacterized mode of action through which eRNAs regulate target gene expression.

Here, in this study we provide the compendium of eRNAs and categorize different eRNA subfamilies through comparing data from global run-on sequencing (GRO-seq), PolyA^+^ and total RNA-seq in differentiating myoblast cells. We demonstrate the presence of a variety of eRNA species with different features of expression level, Pol II association, histone modifications and TF binding etc. We also show the essential role of MyoD in inducing eRNAs production upon myogenic differentiation. Using two eRNAs generated from SEs, *seRNA-1* and *seRNA-2* as paradigm, we further show that seRNAs induced upon differentiation function to promote myogenesis in vitro and in vivo. In depth dissection of how *seRNA-1* regulates the target gene *Mb* transcription leads to the revelation that *seRNA-1* specifically binds to hnRNPL protein and disruption of *seRNA-1*-hnRNPL interaction attenuates Pol II and H3K36me3 deposition at the target genes. A CAAA tract is pinpointed to be crucial in mediating the interaction and function of *seRNA-1*/hnRNPL, thus modulating neighboring gene expression both in vitro and in vivo. CLIP-seq identifies that hnRNPL binds to eRNAs transcriptome-wide in several cell types, suggesting hnRNPL/eRNA binding could be a general mechanism regulating target gene transcription. Collectively, we provide evidence that seRNAs play key roles in orchestrating target mRNA transcription through interacting with hnRNPL.

## Results

### Elucidation of enhancer transcription in muscle cells

**Fig. 1:**
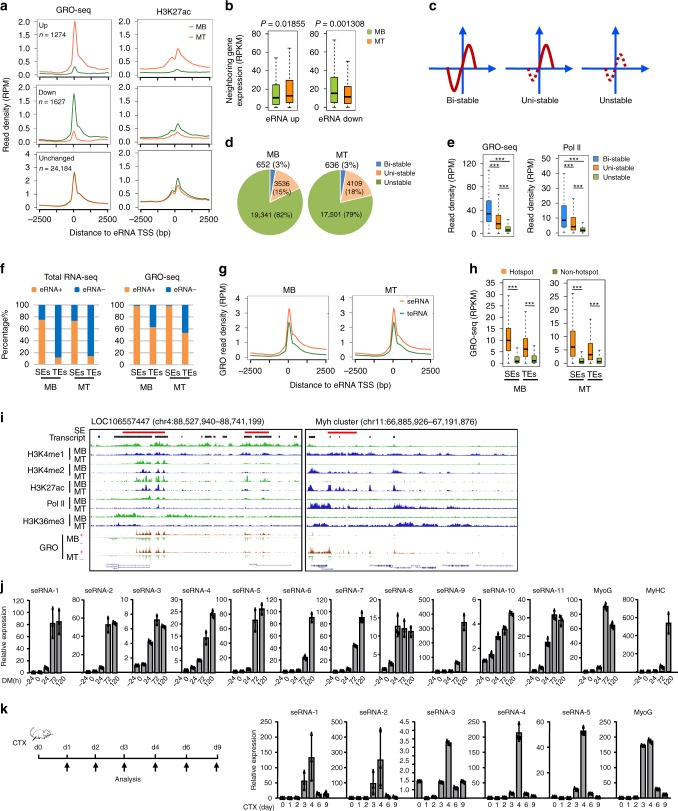
Elucidation of enhancer transcription in muscle cells. **a** GRO-seq detected eRNAs that were up-, down-regulated or unchanged in myotube (MT) vs myoblast (MB) cells, and the changes were correlated with H3K27ac remodeling. **b** Expression of neighboring genes associated with up- or down-regulated eRNAs in MT vs MB. **c** The eRNAs were categorized into ‘stable’ (captured by GRO-seq and total RNA-seq) and ‘unstable’ transcripts (captured only by GRO-seq); the divergent enhancer transcription was further categorized into three types of pairs: Bi-stable (both directions generate stable transcripts), Uni-stable (only one direction generates stable transcript) and Unstable (both directions generate unstable transcripts). **d** Distribution of the above types of divergent eRNAs. **e** Box plot showing the read density (RPM) of GRO-seq signals or Pol II binding on the above three types of eRNAs in MT. **f** Analyzing total RNA-seq or GRO-seq revealed that a higher percentage of SEs in MB or MT gave rise to eRNAs (eRNA+) compared to TEs. **g** seRNAs displayed higher level of GRO-seq signals compared to teRNAs. **h** TF hotspot regions showed markedly higher levels of GRO-seq signals compared to non-hotspot enhancer regions. **i** Genomic snapshots of representative eRNAs identified from MB- (left) or MT-expressed SE (right), showing H3K4me1, H3K4me2, H3K27ac, H3K4me3, Pol II and H3K36me3 ChIP-seq profiles, and GRO-seq in MB and MT cells. The red bar highlights the SE region. The “transcript” track indicates transcript units identified through GRO-seq. GRO-seq signals are displayed in “+” (red) and “−” (light green) strands separately. **j** qRT-PCR measurement of expression dynamics of several MT seRNAs during 120 h differentiation course of C2C12 myoblast. **k** seRNA expressions were measured in the muscles after cardiotoxin (CTX) injection induced regeneration. *n* = 3 per group. Data in **j** and **k** represent the average of three independent experiments ± s.d. Data in **b**, **e**, and **h** are presented in boxplots. Center line, median; box limits, upper and lower quartiles; whiskers, 1.5x interquartile range. Statistical analyses in **b**, **e**, and **h** were done by Mann–Whitney non-parametric test; ***P < 0.001. Source data are provided as a Source Data file.

To gain a panoramic and high resolution view of enhancer transcription and their remodeling during cell differentiation, we took advantage of the well-studied MB differentiation process using C2C12 mouse cell line (Supplementary Fig. 1a, b). GRO-seq was performed with proliferating MBs and differentiating MTs (differentiated for 48 h) in two biological replicates (Supplementary Fig. 1c). The generated data were analyzed to define transcription units, which were then overlapped with enhancer repertoire previously classified in C2C12 cells^6^. As a result, a total of 16,835 TE associated RNAs (teRNAs) together with 6,698 SE associated RNAs (seRNAs) were obtained in MB cells; similarly, 14,997 teRNAs and 7,252 seRNAs were defined in MT cells (Supplementary Fig. 1d and Supplementary Data 1). As expected, due to the power of GRO-seq in detecting nascent transcripts, the number of teRNAs/seRNAs far exceeded the actual number of TEs/SEs^20^. When examining their expression dynamics during MB differentiation, we found 1,274 were significantly up-regulated in MT vs MB and 1,627 down-regulated, while the majority remained unaltered (*n* = 24,184) (Fig. 1a, Supplementary Fig. 1e and Supplementary Data 1). Of note, a concomitant change in levels of H3K27ac (Fig. 1a) and expression of neighboring genes within a ±100 kb window^14^ (Fig. 1b) was observed in concert with changes in eRNA expression, in line with the previous findings suggesting that eRNA production is a key signature of active enhancers. Among them, 423 neighboring genes showed the same expression trend as eRNAs during muscle differentiation (157 up- and 256 down-regulated respectively) (Supplementary Note 1).

Next, attempting to categorize eRNAs, we applied an integrated analysis leveraging multiple RNA-seq datasets using total or PolyA^+^ RNAs from C2C12 cells (Supplementary Fig. 1d). In brief, RNA transcripts were assembled from total RNA-seq to generate lincRNA catalogue. When overlapped with enhancer repertoire, it yielded a catalogue of 1,746 teRNAs and 1,747 seRNAs in MB (Supplementary Fig. 1d), of which 803 teRNAs and 819 seRNAs could be detected in PolyA^+^ RNA-seq (Supplementary Fig. 1d, g). Similarly, a total of 2,406 teRNAs and 2,275 seRNAs were defined in MT among which 965 and 1,016 were found in PolyA^+^ RNA-seq. We reasoned that the eRNAs captured by both GRO-seq and total RNA-seq were relatively stable and those only by GRO-seq as unstable. Consistent with the common notion, many of them were divergently transcribed; the transcription start site (TSS) of divergent pairs were further categorized into three classes: Bi-directional (Bi)-stable (both directions yield stable transcripts detected by total RNA-seq), Uni-directional (Uni)-stable (only one direction yields stable transcript) and Unstable (can only be detected by GRO-seq) (Fig. 1c). Expectedly, the majority of eRNAs were unstable; very small fraction were Bi-stable (Fig. 1d, Supplementary Fig. 1g, h). Our results showed that Bi-stable eRNAs displayed strongest GRO-seq profiles and Pol II binding compared to Uni-stable and Unstable ones (Fig. 1e, Supplementary Fig. 2a). The distinction was also prominent in histone marks and TF binding (Supplementary Note 1).

SEs exhibit magnified enhancer activities compared to TEs^3^. Consistently, we found a higher percentage of SEs generates eRNAs termed seRNAs (Fig. 1f). By GRO-seq, almost all SEs were transcribed whereas only ~60% for TEs. By total RNA-seq, ~70% of SEs in MB or MT produced seRNAs compared to <20% of TEs. Expectedly, seRNAs showed much higher expression than teRNAs, globally (Fig. 1g); SEs also produced a much higher portion of stable eRNAs (both Bi- and Uni-stable ones) than TEs (Supplementary Fig. 1h), suggesting seRNAs are integrated components of SE function. In addition, we found TF hotspots defined in our recent study^6^ were associated with stronger eRNAs expression than non-hotspots (Fig. 1h), further underscoring TF hotspots are super active centers that may modulate enhancer functionality.

We previously demonstrated the stage-specificity of SEs and their gradual remodeling during MB fate transition into MT^6^. Concordantly, seRNAs exhibited distinct expression dynamics in this process, exemplified on the enhancers of the *LOC106557447* and Myosin heavy chain (Myh) gene cluster (*Myh1*, *Myh2*, *Myh4*, *Myh8*, and *Myh13*) (Fig. 1i). Myh genes are associated with muscle contraction in MT and a corresponding stage-specific SE was identified in MT by H3K27ac mark. GRO-seq revealed bidirectional transcription occured in this SE during differentiation, concomitant to induction of active marks like H3K4me1/2, H3K27ac, Pol II and H3K36me3 (Fig. 1i). On *LOC10655774* gene, reduction in these active marks and seRNA expression, by contrast, was observed on the associated SE (Fig. 1i). By quantitative PCR (qPCR) in cells differentiating for various time points (DM −24, 0, 24, 72, and 120 h), seRNAs associated with MT stage (seRNA1-11) were indeed robustly induced upon differentiation (Fig. 1j); MB seRNAs were largely decreased in fully differentiated MT (DM 120 hr) but some displayed an interesting up-regulation in the early differentiation stages (Supplementary Fig. 3a). To further solidify the above seRNA expression dynamics in muscle cells, we also analyzed their expressions in freshly isolated muscle stem cells (also called satellite cells, SCs) (Supplementary Fig. 3b). Consistent with the results from C2C12 cells, nine out of 11 MT seRNAs showed increased expression during SC differentiation (72 h vs 48 h). For MB seRNAs, seven out of 10 were detectable and indeed five showed a decrease in the process (Supplementary Fig. 3c). Furthermore, to assess seRNA expression profile in vivo, we took advantage of a widely used muscle regeneration model in which cardiotoxin (CTX) or BaCl_2_ administration induces muscle injury followed by muscle regeneration^21–25^. The expression of most MT seRNAs was barely detected before day 2 but sharply induced at day 3–4 after CTX injury (Fig. 1k), concomitant with the peak of myoblast differentiation thus in agreement with the above findings from C2C12 in vitro (Fig. 1j). Moreover, abundant levels of these seRNAs were observed in limb muscles of newborn mice (age 3 days to 2 weeks), which underwent active myogenesis but dropped after 2 weeks when the neonatal myogenesis ceased (Supplementary Fig. 3d and Supplementary Note 1). Collectively, the above results demonstrate that eRNAs are pervasively transcribed in muscle cells and may act as integral component of enhancers, warranting further investigation of their functional mechanisms.

### MyoD plays a crucial role in inducing MT eRNAs

**Fig. 2:**
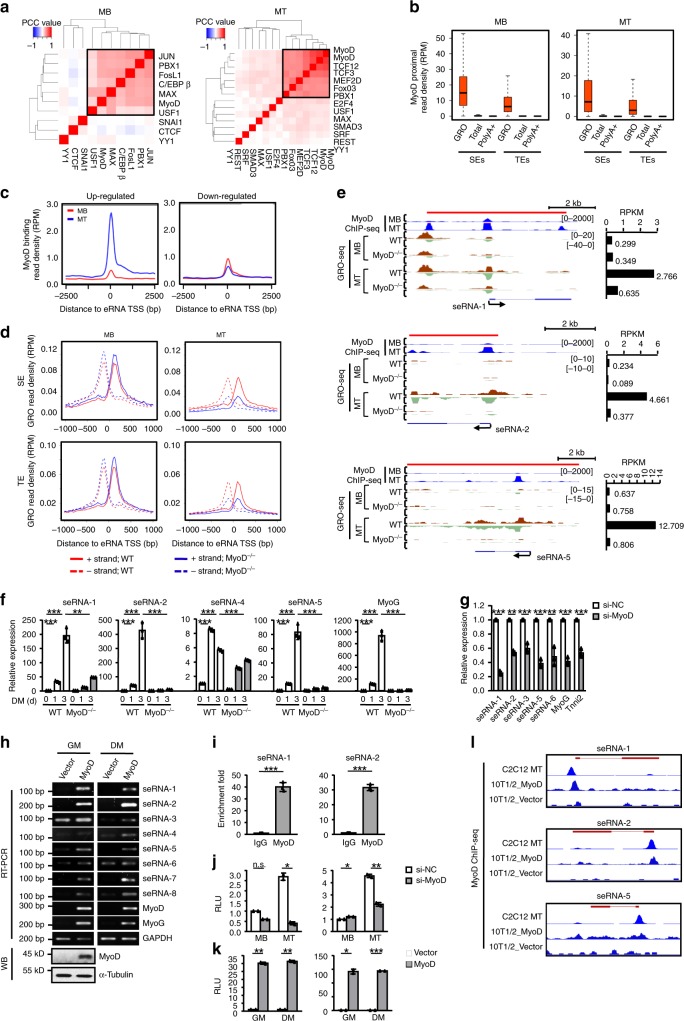
MyoD plays a crucial role in inducing MT eRNAs. **a** Unsupervised clustering of TF binding at eRNA TSSs in MB or MT. The color code indicates the Pearson correlation coefficient (PCC) between two TFs at their binding sites. **b** Comparison between GRO-seq, total and PolyA^+^ RNA-seq signals in the MyoD binding proximal regions (±1 kb of the center of MyoD-binding sites) at SEs and TEs. **c** MyoD ChIP-seq signals within ±2.5 kb flanking TSSs of up-regulated (left) and down-regulated (right) eRNAs. **d** Distribution of averaged GRO-seq signals from SEs or TEs in WT or MyoD knockout (MyoD^−/−^) cells. **e** Illustration of eRNA down-regulation in MyoD^−/−^ vs WT cells by GRO-seq tag counts on seRNA-1, -2, and -5. The bar graph shows the quantification of GRO-seq signals in RPKM. **f** qRT-PCR measurement of seRNAs in the differentiating MyoD^−/−^ vs WT cells. **g** qRT-PCR detection of seRNAs from 48-h-differentiated C2C12 cells transfected with either control or MyoD siRNA. **h** Top: 10T1/2 cells were transfected with either control or MyoD expressing plasmid; the cells were collected in growth medium (GM) or differentiate medium for 48 h (DM). The relative expression of seRNAs, MyoD and Myogenin were measured by semi-quantitative RT-PCR. Bottom: Western blot confirmed MyoD overexpression. **i** MyoD ChIP-PCR at the TSS of seRNA-1 or seRNA-2 in MT cells. **j** Luciferase reporter activity of seRNA promoter was detected in 48-hr-differentiated C2C12 cells transfected with either control or MyoD siRNA. **k** Luciferase reporter activity of the above seRNA promoter in 10T1/2 cells overexpressing MyoD. **l** Distribution of MyoD ChIP-seq signals on the TSSs of seRNA-1, -2, and -5 in 10T1/2 cells overexpressing MyoD. Data in **f**, **g**, **i** represent the average of three independent experiments ± s.d. Data in **b** are presented in boxplot. Center line, median; box limits, upper and lower quartiles; whiskers, 1.5x interquartile range. Statistical analysis was performed by two-way ANOVA with Sidak’s post-hoc test (**f**) or two-tailed unpaired Student’s *t*-test (**g**, **i**, **j**, **k**), n.s., not significant, *P < 0.05, **P < 0.01 and ***P < 0.001. Source data are provided as a Source Data file.

Next, we sought to identify key TFs that determine eRNAs expression dynamics during myogenic differentiation. Applying HOMER^26^, we predicted potential TF binding motifs enriched within the 2 kb window of TSSs of eRNAs that were highly expressed in MB or MT (log_2_ (Fold change) > 2 or <−2 with adjusted *P* value < 0.05) (Supplementary Fig. 4a). A proportion of the top-ranked motifs were bound by known key regulators in the respective cell stage^6^, for instance, MyoD and MyoG in MT and JUN in proliferating MB (Supplementary Fig. 4a). In addition, analyzing the available TF ChIP-seq datasets from C2C12, we noticed convergence of multiple TFs at TSSs of eRNAs in both MB and MT (Fig. 2a, Supplementary Fig. 4b). One distinct combinational module unveiled in MT involved MyoD, MyoG, TCF12, TCF3, MEF2D, PBX1, and FoxO3 (Fig. 2a, Supplementary Fig. 4b), which was in line with our recent finding showing this module partakes in SE assembly in MT^6^. Similar TF associations and a combinational module were also identified in MB (Fig. 2a, Supplementary Fig. 4b). To understand the full spectrum of eRNAs under regulation by master TF MyoD which was suggested in an earlier report^27^, we intersected MyoD ChIP-seq profiles with the above GRO-seq dataset. Our analyses revealed evident signals around MyoD binding sites at enhancers with the read density much higher on SEs than TEs (Fig. 2b). The read density dropped significantly if using total or PolyA^+^ RNA-seq, indicating GRO-seq is a more sensitive approach in detection of eRNA induction by MyoD and the majority of the eRNAs are probably unstable. Among the enhancer transcription units derived from GRO-seq, we observed 34.7% of seRNAs in MB and 59.1% in MT possessed MyoD binding at their TSS while 31.5% and 45.5% for teRNAs, respectively (Supplementary Fig. 4c), again suggesting the prevalent binding of seRNA TSS by MyoD in both MB and MT. Moreover, for up-regulated eRNAs in MT vs MB, MyoD binding was sharply enhanced at their cognate TSSs (Fig. 2c); by contrast, the binding at the TSSs of down-regulated eRNAs was slightly reduced (Fig. 2c), reflecting a crucial role of MyoD binding in eRNA induction during myoblast differentiation. To strengthen the above findings, GRO-seq was performed in *MyoD* knockout (*MyoD*^−/−^) cells that we recently generated by CRISPR-Cas9 (Supplementary Fig. 1c and Supplementary Data 3);^6^ the mutant cell displayed an expected differentiating defect assessed by immunofluorescence (IF) staining of MyoG and MyHC-positive cells (Supplementary Fig. 4d). In response to *MyoD* loss, the expression of MT eRNAs (both teRNAs and seRNAs) was dramatically diminished, but modestly enhanced for MB eRNAs (Fig. 2d, Supplementary Fig. 4e, f and Supplementary Data 3). As illustrated on *seRNA-1*, *-2*, and *-5* (Fig. 2e), *MyoD* depletion almost blocked the dramatic induction of their transcription during differentiation, which was confirmed by qRT-PCR (Fig. 2f, see additional examples on other MT associated seRNAs in Supplementary Fig. 4g). We also applied knock-down strategy using short interfering RNAs (siRNAs) against *MyoD*. We found that knockdown of *MyoD* also decreased MT seRNAs (Fig. 2g), again supporting the activating role of MyoD in seRNAs induction during differentiation. Depletion of *Myogenin*, which was also enriched at the TSS of eRNA, on the other hand, decreased the expression of several but not all seRNAs (Supplementary Fig. 4h). Finally, when *MyoD* was overexpressed in mouse embryonic 10T1/2 fibroblast cells to trigger myogenic trans-differentiation, we noted induction or increase in seRNAs expression (Fig. 2h).

To further elaborate the direct regulation of MyoD, we confirmed the MyoD binding identified by ChIP-seq on *seRNA-1* and *-2* loci by ChIP-PCR (Fig. 2i, Supplementary Fig. 4i). In addition, we cloned their TSS regions *-2* harboring MyoD binding sites into a reporter construct. Expectedly, reporter activity was significantly increased during differentiation but markedly reduced upon *MyoD* repression (Fig. 2j, Supplementary Fig. 4i). Promoter reporters also displayed concomitantly increased activity upon *MyoD* overexpression in 10T1/2 cells (Fig. 2k). Moreover, direct binding of MyoD was detected on these promoters during trans-differentiation^28^ (Fig. 2l), indicating MyoD induction of seRNAs is a prominent phenomenon during MyoD directed cell reprogramming. Together, these data solidify the key role of MyoD in inducing eRNA production during myoblast differentiation.

### seRNA-1 and seRNA-2 regulate target gene expression

**Fig. 3:**
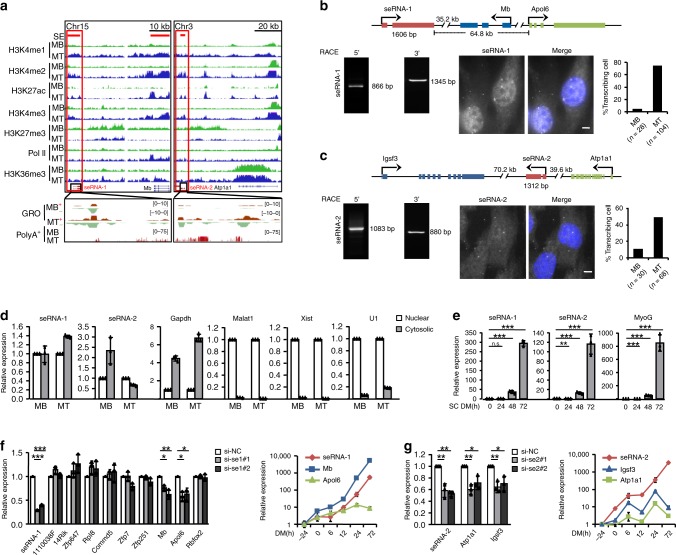
seRNA-1 and seRNA-2 regulate target gene expression. **a** Genomic snapshots of mouse seRNA-1 and seRNA-2 genes showing Pol II and histone marks ChIP-seq profiles, Poly A^+^ RNA-seq and GRO-seq in MB and MT cells. The red bar highlights the SE region and the red box indicates the seRNA locus. GRO-seq signals are displayed in “+” (red) and “−” (light green) strands separately. **b** Top: schematic illustration of genomic structure of mouse seRNA-1 relative to the neighboring genes Mb and Apol6. Bottom: Left: Product of RACE cloning (5′ and 3′) of seRNA-1; Right: Detection of seRNA-1 molecules (red) in MT by single molecule RNA FISH. Scale bar, 5 μm. **c** Top: schematic illustration of genomic structure of mouse seRNA-2 relative to the neighboring genes Atp1a1 and Igsf3. Bottom: Left: Product of RACE (5′ and 3′) cloning of seRNA-2; Right: FISH detection of seRNA-2 in MT. Scale bar, 5 μm. Quantification of FISH signals in **b**, **c** corresponding to seRNA transcripts in MB and MT cells. Cells with at least one spot in the nucleus were regarded as “transcribing”. DNA (blue) was stained with DAPI. A representative image was shown. **d** qRT-PCR analysis of RNAs purified from nuclear and cytosolic fractions of C2C12 cells. **e** qRT-PCR detection of seRNA-1 and seRNA-2 in the differentiating SCs isolated from muscles of Tg: Pax7-nGFP mice. **f** Left: qRT-PCR detection of seRNA-1 and neighboring genes from 48-h-differentiated C2C12 cells transfected with either control or seRNA-1 siRNA (si-se1#1 or si-se1#2). Right: qRT-PCR measurement of expression kinetics of seRNA-1 and the Mb and Apol6 during C2C12 differentiation. **g** The above experiments were performed for seRNA-2 and its neighboring Atp1a1 and Igsf3 genes. Data represent the average of three independent experiments ± s.d. Statistical analysis was performed by one-way ANOVA with Tukey’s post-hoc test (**e**), or two-tailed unpaired Student’s *t*-test (**f**, **g**) n.s., not significant, *P *<* 0.05, **P *<* 0.01 and ***P < 0.001. Source data are provided as a Source Data file.

Given that many MT seRNAs were markedly induced during C2C12 myoblast differentiation (Fig. 1j), we speculated that they may play active roles in driving the differentiation as essential components of SEs. Two seRNAs, *seRNA-1* and *seRNA-2* were among the highest induced thus selected for in depth functional characterization (Supplementary Fig. 5a). As shown in Fig. 3a, they were originated from two SEs on mouse chromosomes 15 and 3 and bi-directionally transcribed as shown by GRO-seq. PolyA^+^ RNA-seq however only captured uni-directional transcripts in the SEs respectively; they were barely detectable in MB but highly expressed in MT; no human counterparts of *seRNA-1* and *-2* appeared to exist despite 8.44% eRNAs in fact displayed evolutionary conservation between human and mouse genomes (Supplementary Fig. 5b). As expected, up-regulation in their expression was in concert with the changes in active histone modifications, H3K4me1, H3K4me2, H3K27ac, H3K4me3, and H3K36me3 and Pol II binding, but anti-correlated with change in repressive histone mark H3K27me3 (Fig. 3a). Moreover, integrative transcriptomic analysis of publicly available RNA-seq data from various tissues suggested that *seRNA-1* and *-2* were expressed in multiple tissues (Supplementary Data 2). To further characterize their functions, we cloned them using rapid amplification of complementary DNA ends (RACE), which revealed *seRNA-1* was a 1606 nt transcript with two exons and *seRNA-2* 1384 nt with two exons (Fig. 3b, c). Both transcripts possessed a polyadenylation site. RNA fluorescence in situ hybridization (FISH) uncovered the two seRNAs resided in both the cytoplasm and nucleus (Fig. 3b, c, Supplementary Fig. 5c, d). *seRNA-1* signal was detected in both nucleus and cytoplasm but appeared to be higher in the nucleus; several intensive focal signals were found in nucleus presumably representing its transcription or action site^29^. *seRNA-2* signal was more dispersed in the cell with similar focal signals detected. Consistent with their induction in MT vs MB, the percentage of *seRNA-1*-positive cells increased from 4% to 74% in MT vs MB, and from 10% to 48% for *seRNA-2*. As control, *Malat1* was exclusively detected in the nucleus (Supplementary Fig. 5c). The above findings were further confirmed by cellular fractionation assay. As positive controls, *U1*, *Malat1*, and *Xist* RNAs were mainly found in the nuclear portion while *Gapdh* in the cytoplasm of both MB and MT; *seRNA-1* showed a comparable level in both nuclear and cytoplasm lysates in MT while *seRNA-2* was slightly enriched in nuclear fractions (Fig. 3d). Moreover, *seRNA-1* was present at ~0.12 copy/cell in MB, and ~7.97 copies/cell in MT (DM day 2); *seRNA-2* was around 2.44 copies/cell in MB and 10.04 copies/cell in MT (Supplementary Fig. 5e, f). In addition, these two seRNAs were predicted as non-coding with no micropeptides produced by our in-house iSeeRNA software^30^ (Supplementary Fig. 5g). In fact, most seRNAs identified were predicted to be non-coding (Supplementary Fig. 5g). To gain insights into their functions in muscle cells, we next examined their expression dynamics in various myogenesis settings in vitro and in vivo. First, as shown earlier in Fig. 1m, during C2C12 differentiation, *seRNA-1* expression was lowly expressed in proliferating MBs at 50% confluence (−24 h); an evident increase was detected when the confluence reached 70–80% (0 h) and the sharp elevation was sustained to late differentiation stage (120 h). Concordantly, its expression was dramatically increased during the differentiation of SCs (Fig. 3e). The temporal kinetics of *seRNA-2* followed the same profile with *seRNA-1* during C2C12 and SCs differentiation (Figs. 1j, 3e).

The production of eRNAs has been shown to activate the transcription of neighboring genes^9–13^. To test this functional scenario on seRNAs in myogenesis, we used siRNA to deplete *seRNA-1* and *seRNA-2* and interrogated the effect on the neighboring genes within ±150 kb window^14^. Efficient knock-down was achieved on total or nuclear level of *seRNA-1* or *seRNA-2* (Fig. 3f, g, Supplementary Fig. 6a). Among the nine genes surrounding *seRNA-1*, knock-down of *seRNA-1* resulted in a decrease in levels of two nearest mRNAs, myoglobin (*Mb*) and apolipoprotein L6 (*Apol6*) in both siRNA groups, but no effect on others (Fig. 3b, f). Similarly, silencing of *seRNA-2* led to reduction of the neighboring genes, ATPase Na+/K+ transporting subunit alpha 1(*Atp1a1*) and immunoglobulin superfamily member 3 (*Igsf3*) (Fig. 3c, g). Meanwhile, *seRNA-1* depletion had no effect on *Atp1a1* and *Igsf3* and *seRNA-2* loss did not decrease *Mb* or *Apol6* either, attesting to the specificity of the seRNA knockdown effect (Supplementary Fig. 6b). When overexpressed in myotubes ectopically *in trans*, however, we did not observe drastic changes in their targets (Supplementary Fig. 6c, d) presumably due to the inability of ectopically expressed RNAs to access the target loci^23,31^. The above results implied that *seRNA-1* and *seRNA-2* probably exerted their impact on the nearest target genes *in cis* within the proximity of their transcription site, which is analogous to the action mechanism of many known eRNAs/lncRNAs^10,23,31^. Lastly, when examining the expression kinetics of the target genes during muscle differentiation and regeneration, we observed that the induction of *seRNA-1* coincided with the upregulation in *Mb* and *Apol6* mRNAs during C2C12 early differentiation (Fig. 3f) and SC differentiation (Supplementary Fig. 6e). Similarly, for *seRNA-2* locus, induction in *Atp1a1* and *Igsf3* was coincident with change in *seRNA-2* during C2C12 early differentiation (Fig. 3g); similar result was observed for *Atp1a1* during SC differentiation but not for *Igsf3* (Supplementary Fig. 6e). When evaluating the expression dynamics in vivo during injury induced muscle regeneration, we found *Mb* and *Apol6* shared identical kinetics; they were highly expressed in homeostatic muscles, sharply lost upon muscle injury and continually elevated during regeneration (Supplementary Fig. 6f). This pattern was distinct from *seRNA-1* (Fig. 1k), implying *seRNA-1* may have a temporal role in their activation but may not be necessary for maintaining their expression when muscle injury is restored. For *seRNA-2* locus, the neighboring genes exhibited similar trend with *seRNA-2* (Fig. 1k, Supplementary Fig. 6f), with expression peaking around day 4. Taken together, the above results confirm seRNAs influence the expression of their neighboring target genes in cis, warranting further exploration of the underlying mechanisms of action (Supplementary Fig. 7 and Supplementary Note 2).

### seRNA-1 and seRNA-2 interact with hnRNPL

**Fig. 4:**
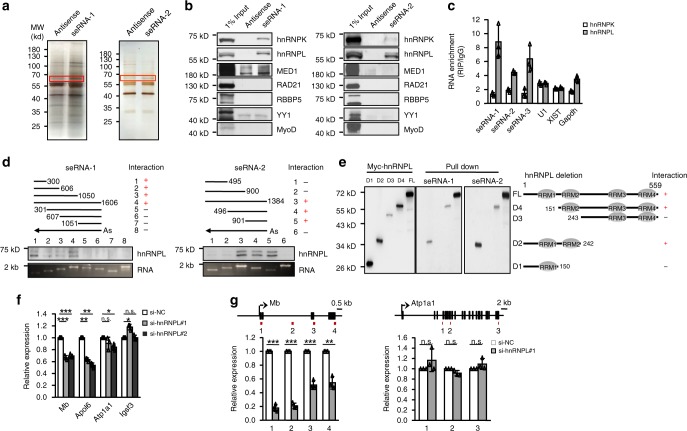
seRNA-1 and seRNA-2 interact with hnRNPL. **a** RNA pull-down assay was performed using biotinylated seRNA or antisense control RNAs in nuclear lysates of MT cells and the purified proteins were run on SDS-PAGE. The highlighted bands were extracted and subjected to mass spectrometry (MS) analysis. **b** Western blot (WB) analysis confirmed the specific association of seRNA-1 or -2 with hnRNPK and hnRNPL. No association with MED1, RAD21, RBBP5, YY1, and MyoD proteins was detected. **c** RNA immunoprecipitation (RIP) was performed with antibodies against hnRNPK or hnRNPL in non-crosslinked differentiating cells and followed by qRT-PCR analysis of retrieved RNAs. Enrichment was determined as RNAs associated to hnRNPK or hnRNPL IP relative to IgG control. **d** The indicated deletion fragments of seRNA-1 or -2 were in vitro generated and used for RNA pull-down assay to map the interacting region with hnRNPL. **e** The indicated Myc-tagged hnRNPL variants (D1, D2, D3, and D4) were transfected into 293 T cells and the whole cell lysates were used for RNA pull-down assay with biotinylated seRNA to map the hnRNPL domain interacting with seRNA-1 or -2. Left: Western blot showing the overexpression of the above variants. D2 and D4 interacted with seRNA-1 or seRNA-2; Right: schematic of structures of the above variants. RRM, RNA recognition motif. **f** C2C12 cells were transfected with siRNAs targeting hnRNPL or scrambled negative control (si-NC). At 24 h post transfection, the cells were switched to DM for 48 h. The expression of seRNA target genes was measured by qRT-PCR. **g** Nuclear run-on assay was performed to measure nascent transcription of Mb or Atp1a1 in the above cells transfected with si-hnRNPL#1. Data represent the average of three independent experiments ± s.d. Statistical analysis was done by two-tailed unpaired Student’s *t*-test (**f**, **g**), n.s., not significant, *P < 0.05, **P < 0.01 and ***P < 0.001. Source data are provided as a Source Data file.

To search for undefined molecular mechanisms underlying seRNAs mediated transcriptional activation of target genes, we sought to identify protein partners of seRNAs through RNA pull-down assay. In vitro transcribed biotinylated *seRNA-1* or *seRNA-2* RNAs were incubated with nuclear extracts from MT cells and co-precipitated proteins were isolated. Compared to the respective antisense control, a band around 55–70 kD was unique in the seRNA pull-down and subject to mass spectrometry (MS) analysis (Fig. 4a). This approach uncovered nine and eleven proteins that were potentially associated with *seRNA-1* and *seRNA-2*, respectively (Supplementary Fig. 8a, b). PLEC, VIM and CKAP4 were among the top ranked probably because these were highly abundant cytoskeletal associated proteins in muscle cells. Intriguingly, both *seRNA-1* and *seRNA-2* strongly retrieved heterogeneous nuclear ribonucleoprotein family members, hnRNPK and hnRNPL (Supplementary Fig. 8b). We therefore decided to further explore the seRNA/hnRNP association attempting to uncover mechanistic insights. First, the interactions with seRNAs were further confirmed by Western blotting (WB); the association between seRNAs with hnRNPL was much stronger than that with hnRNPK (Fig. 4b). The known eRNA binding partners, Mediator Complex Subunit 1 (MED1), RAD21 cohesin complex component (RAD21) were however not found to bind with the seRNAs; no interaction was detected with several other known lncRNA binding proteins including retinoblastoma binding protein 5, histone lysine methyltransferase complex subunit (RBBP5)^32^, YY1^18,21^, and MyoD^33,34^ either (Fig. 4b), suggesting the specificity of seRNA/hnRNPL association. Furthermore, by native RNA immunoprecipitation (RIP) assay using cell extracts of MT cells, a high level of *seRNA-1*, *-2*, and *-3* was retrieved by hnRNPL but interestingly not hnRNPK antibody (Fig. 4c, Supplementary Fig. 8c), suggesting in vivo hnRNPK interaction with seRNA was probably at low affinity thus could not be detected by relatively more stringent RIP conditions. The above results triggered us to believe hnRNPL was a bona fide protein partner of seRNAs. To further solidify their binding nature, next, we used a series of truncated fragments of *seRNA-1* or *seRNA-2* to map hnRNPL-interacting regions. Our results suggested a 300 nt region at the 5′ of *seRNA-1* (1–300 nt) and a 484 nt region at the 3′ of *seRNA-2* (901–1384 nt) were essential in mediating the interaction with hnRNPL (Fig. 4d); of note, the interaction of hnRNPL with 5’ *seRNA-1* was comparable or stronger than the full length (FL). Consistently, these domains exhibited high thermostability predicted by RNAfold (Supplementary Fig. 8d). HnRNPL protein consists of four RNA recognition motifs (RRM1–4) (Fig. 4e). By pull-down assay, we found the second RRM (RRM2) was pivotal for binding with both seRNAs and the presence of RRM1 seemed to augment the interaction, especially with *seRNA-2* (Fig. 4e).

**Fig. 5:**
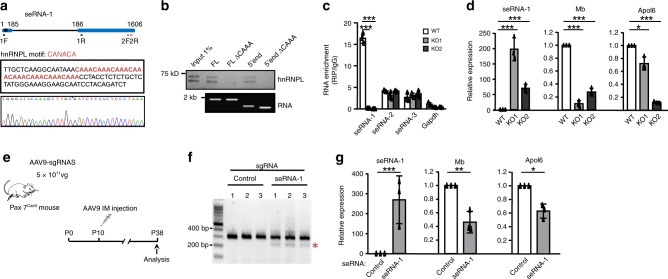
CAAA tract is indispensable for seRNA-1 function. **a** Schematic illustration of CAAA deletion medicated by CRISPR-Cas9 editing in C2C12 cells. Top: black box depicts the deletion region. Primer set 1 (1F and 1R) was used for cDNA genotyping. Primer set 2 (2F and 2R) was used for qRT-PCR analysis. Middle: CAAA tract sequence is highlighted in red. sgRNAs were designed to delete the underlined sequence encompassing the CAAA tract. Bottom: Result from Sanger sequencing confirmed the CAAA deletion. **b** Biotinylated seRNA-1 transcripts of full length (FL) or 5′ end fragment with or without CAAA deletion (ΔCAAA) were used in RNA pull-down assay to reveal seRNA-1 binds hnRNPL through the CAAA tract embedded in its 5′ region. **c** hnRNPL RIP was performed in WT or the generated CAAA deletion (KO) cells to show hnRNPL/seRNA-1 binding was abolished in the KO cells as compared to WT control cells. Enrichment was determined as RNA associated to hnRNPL IP relative to IgG control. **d** Expression of the associated target genes, Mb and Apol6, was decreased but seRNA-1 was increased in the KO cells as measured by qRT-PCR. **e** Schematic illustration of the CRISPR-Cas9 mediated in vivo deletion of CAAA tract. Pax7^Cas9^ mouse at postnatal 10 (P10) age was intramuscularly (IM) injected with 5 × 10^11^ vg AAV9-sgRNAs viruses and the infected muscles were recovered for analysis at 4 weeks later (P38). *n* = 3 per group. **f** Detection of CAAA excision by genomic PCR in muscle tissues injected with AAV9-sg-seRNA-1, compared to AAV-sg-Control. Un-edited product, 304 bp; deletion product (red asterisk), 220 bp. **g** qRT-PCR was performed to measure the levels of seRNA-1, Mb and Apol6 in the above injected muscles. *n* = 3 per group. Data represent the average of three independent experiments ± s.d. Statistical analysis was done by two-tailed unpaired Student’s *t*-test (**c, d, g**), *P < 0.05, **P < 0.01, and ***P < 0.001. Source data are provided as a Source Data file.

We next sought to dissect the functional relevance of the interaction between seRNAs and hnRNPL. When hnRNPL was knocked down (Supplementary Fig. 8e), *seRNA-1* target genes, *Mb* and *Apol6*, were evidently reduced (Fig. 4f) while *seRNA-1* itself was up-regulated (Supplementary Fig. 8f), suggesting a functional synergism of hnRNPL with *seRNA-1* in activating target gene expression. However, target genes associated with *seRNA-2*, *Atp1a1*, and *Igsf3*, were unaffected upon hnRNPL loss (Fig. 4f), indicating hnRNPL/seRNA co-action may not apply to this seRNA. This was further strengthened by nuclear run-on assay, which demonstrated that reduced hnRNPL dampened the transcription of *Mb*, but not *Atp1a1* (Fig. 4g). We reasoned that this was probably due to a relatively weaker interaction between *seRNA-2* and hnRNPL as *seRNA-2* contains only one copy of CACACA tract at 3′end that resembles hnRNPL binding CA-rich motif^35,36^ while *seRNA-1* possesses eight tandem-repeats of CAAA tracts at its 5′end. To further test if the 8 CAAA repeats (Fig. 5a) of *seRNA-1* mediated the interaction between *seRNA-1* and hnRNPL, the CAAA tracts were removed from the FL or the 5′ fragment (Fig. 4d, ∆CAAA) and used for in vitro RNA pull-down experiments. Expectedly, the deletion completely abolished the strong interaction between FL or the 5′ fragment with hnRNPL (Fig. 5b). Furthermore, we employed CRISPR-Cas9 approach to introduce a small deletion in *seRNA-1* locus encompassing the CAAA repeats in C2C12 cells, generating two knockout (KO) clones (Fig. 5a, Supplementary Fig. 8g). RIP assay in the KO cells with hnRNPL antibody failed to retrieve *seRNA-1* transcripts as compared to WT cells (Fig. 5c), suggesting the essential role of this CAAA tract in mediating *seRNA-1*/hnRNPL interaction. Nevertheless, the expression of hnRNPL was unaltered by the CAAA deletion (Supplementary Fig. 8h), ruling out the possibility that the loss of interaction was due to reduction in hnRNPL level. Knowing RNA alternative splicing is a major function of hnRNPL-RNA interaction, we examined whether *seRNA-1* was alternatively spliced in the KO cells and found loss of CAAA tract did not affect its splicing, leaving junction sites intact (Fig. 5a, Supplementary Fig. 8g). Nevertheless, removal of CAAA tract appeared to have altered the secondary structure of *seRNA-1* (Supplementary Fig. 8i). To test the functional importance of CAAA tract, we found *Mb* and *Apol6* expression was largely attenuated in the KO cells (Fig. 5d), underscoring the importance of the interaction between CAAA tract and hnRNPL. Surprisingly, *seRNA-1* itself was markedly increased in KO cells (Fig. 5d). We reasoned this region may mediate a transcriptional repression on *seRNA-1* locus itself, for example, the region may harbor repressor TF binding; indeed in silico TF prediction revealed that the binding sites of multiple TFs, including ETS2, FoxO4, and FoxP1 may be eliminated when CAAA tract was removed; alternatively, the hnRNPL/*seRNA-1* interaction may exert a feedback regulation on *seRNA-1* transcription.

To expand the above findings, we next determined the significance of CAAA tract in regulating *seRNA-1* neighboring genes in vivo. To this end, we deleted the CAAA tract taking advantage of CRISPR/Cas9 mediated in vivo genome editing^37^. Briefly, Cre-dependent Pax7^Cas9^ mouse was generated through crossing Pax7^Cre^ mouse^38^ with homozygous Rosa^Cas9-eGFP^ mouse^37^, resulting in the labeling of all Pax7 derived cells (muscle lineage) with eGFP. In vitro validated sgRNA pairs targeting the CAAA tract were generated from a U6-driven AAV backbone using muscle-tropic AAV9 as the delivery vector. The Pax7^Cas9^ mice were injected intramuscularly with a single dose (5 × 10^11^ vg per mouse) of AAV9-sgseRNA-1 at postnatal day 10 (P10) and were analyzed 4 weeks later at P38^39^ (Fig. 5e). Compared to AAV9-sgControl group, we observed an expected 86 bp deletion across the genomic locus in mice injected with AAV-sgseRNA-1 (Fig. 5f). In addition, RNA analysis showed a fraction of transcripts with CAAA deletion, confirming the success of in vivo editing (Supplementary Fig. 8j). Importantly, consistent with in vitro results from using KO cells, loss of CAAA tract significantly decreased the expression of *Mb* and *Apol6*, but strongly induced *seRNA-1* level (Fig. 5g), suggesting the existence of regulatory axis involving CAAA tract-hnRNPL interaction in vivo. Altogether the above results indicate that *seRNA-1* interacts with hnRNPL via a CAAA tract in its 5′–300 nt region and this motif is critical for hnRNPL binding and activation of neighboring genes both in vitro and in vivo.

### Mapping of transcriptome-wide binding of hnRNPL with eRNAs

Although the above investigation was mainly performed on *seRNA-1*, we were curious if eRNAs/hnRNPL interaction could be a general mechanism mediating enhancer function in modulating target gene expression. To test this notion, we conducted CLIP (crosslinking immunoprecipitation)-seq^40^ in differentiating C2C12 cells to map transcriptome-wide RNA binding events of hnRNPL. RNAs were isolated from hnRNPL protein-RNA complexes (Supplementary Fig. 9a–d) while no protein–RNA complexes were retrieved from nonspecific IgG or HA tag immunoprecipitation (IP) controls, confirming the high specificity of the used hnRNPL antibody (Supplementary Fig. 9b). Through crosslinking induced mutation site (CIMS) analysis pipeline^40^, a set of hnRNPL-bound RNA regions were identified, consisting of 29,224 clusters with more than 5 reads (Supplementary Fig. 9c, Supplementary Data 4). No positive correlation was seen between CLIP-seq signals and transcript expression levels (Supplementary Fig. 9e), suggesting the specificity of CLIP allowing the detection of not only relatively stable and abundant transcripts. Consistently, motif analysis demonstrated that the canonical hnRNPL binding CACACA motif was indeed enriched within the center of the CLIP tags in the transcriptome (Supplementary Fig. 9f, g). The majority of hnRNPL-bound RNAs were localized in genic region with a significant portion arising from introns (70.70%) (Supplementary Fig. 9h), consistent with its well-known roles in regulating mRNA splicing^41,42^. A significant portion of CLIP tags (16.15%) on the other hand fell into non-genic category including noncoding genes and intergenic regions (Supplementary Fig. 9h). When analyzing the non-genic hnRNPL CLIP signals in depth, we observed ~23% of these RNA tags overlapped with enhancer repertoire (15.85% with SE and 7.10% with TE, respectively) (Supplementary Fig. 9h, Supplementary Data 4) even though only 5.49% of eRNAs were bound by hnRNPL; moreover, these hnRNPL CLIP positive sites in enhancers displayed much higher GRO-seq reads than those with no CLIP binding (Supplementary Fig. 9i), testifying hnRNPL binding with eRNAs could be a transcriptome-wide event. In addition, SEs displayed much higher hnRNPL CLIP signals compared to TEs and the RNA tags generated from SEs were prone to be stable eRNAs (Supplementary Fig. 9j–l), suggesting a positive relationship between hnRNPL-CLIP signal and enhancer transcription (Supplementary Figs. 9, 10 and Supplementary Note 3).

We then tested whether hnRNPL-eRNA binding was associated with the expression of target genes. Through integrating hnRNPL CLIP-seq and RNA-seq in MT, we noticed the increase in expression of neighboring mRNA genes was positively correlated with the number of hnRNPL clusters at the corresponding eRNAs (Supplementary Fig. 9o), suggesting a potential activating role of hnRNPL-eRNA binding in the expression of neighboring genes transcriptome-wide. To test this, we performed RNA-seq in *hnRNPL* knockdown cells. Compared to si-Control, *hnRNPL* depletion resulted in 153 genes up-regulated and 103 down-regulated, respectively (Supplementary Fig. 9p, Supplementary Data 5). Expression of genes associated with hnRNPL-bound eRNAs tended to be altered upon hnRNPL knockdown relative to those associated with non-hnRNPL binding eRNAs (Supplementary Fig. 9q). Moreover, these neighboring genes themselves were not enriched for hnRNPL CLIP binding (Supplementary Fig. 9r), ruling out these mRNAs were regulated through direct interaction with hnRNPL. Overall, our results suggest a possibility that hnRNPL-eRNA interaction could play a general role in regulating target genes expression.

### seRNA-1 modulates hnRNPL, Pol II, and H3K36me3 binding at Mb

**Fig. 6:**
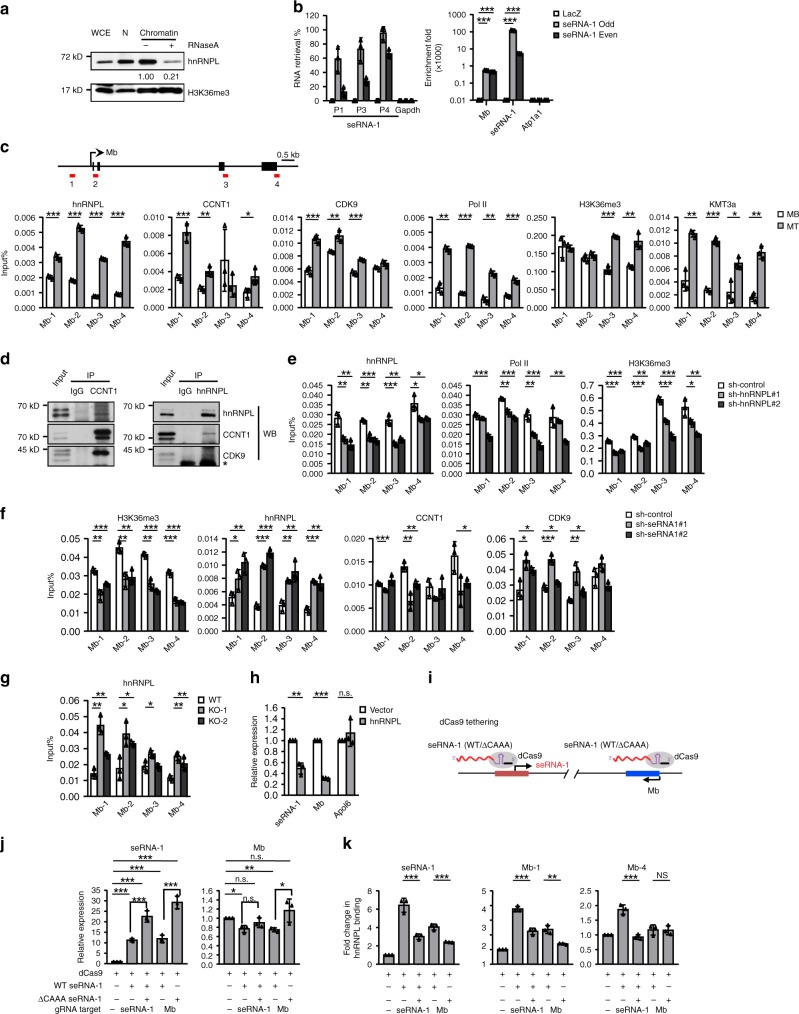
seRNA-1 modulates hnRNPL, RNA Pol II and H3K36me3 at Mb locus. **a** Western blot analysis of hnRNPL cellular distribution in differentiating C2C12 untreated or treated with RNaseA. Whole cell extracts (WCE), nuclei (N). Relative levels of hnRNPL were normalized with H3K36me3 and measured by Image J. **b** qPCR quantification of RNA (left) and DNA (right) recovered after lacZ ChIRP or seRNA-1 ChIRP with two different biotinylated probe sets (even and odd) in C2C12 MT. P1, P3 and P4 are three different primer pairs for detecting seRNA-1 RNA. Mb, seRNA-1 and Atp1a1 indicate the primers corresponding to the promoter regions. **c** ChIP-PCR of hnRNPL, CCNT1, CDK9, Pol II, H3K36me3, and KMT3a at regions (1, 2, 3, and 4) across Mb locus in MT vs MB. **d** Co-IP assay was performed using antibodies against hnRNPL or CCNT1 in C2C12 MT and the interaction between endogenous hnRNPL and CCNT1 or CDK9 was detected. * IgG light chain. **e** ChIP-PCR of hnRNPL, Pol II, and H3K36me3 at the above Mb loci in control or hnRNPL knockdown cells (#1 or #2). **f** ChIP-PCR of hnRNPL, CDK9, CCNT1 and H3K36me3 at the above Mb loci in control or seRNA-1 knockdown cells (#1 or #2). **g** ChIP-PCR of hnRNPL at the above Mb loci in the two CAAA KO cell lines compared to WT control. **h** Overexpression of hnRNPL in C2C12 cells decreased the expression of seRNA-1 and Mb but not Apol6. **i** Schematic illustration of dCas9 mediated tethering of seRNA-1 wild type or the CAAA KO mutant (ΔCAAA) to the seRNA-1 TSS or Mb promoter. **j** qRT-PCR detection of seRNA-1 and Mb in the above cells. **k** ChIP-PCR of hnRNPL binding at the indicated seRNA-1 or Mb promoter following the above tethering. Data represent the average of three independent experiments ± s.d. Statistical analysis was done by two-tailed unpaired Student’s *t*-test (**b**, **c**, **e**–**h**, **j**, **k**). n.s., not significant, *P < 0.05, **P < 0.01, and ***P < 0.001. Source data are provided as a Source Data file.

After establishing the role of hnRNPL-eRNAs interaction in modulating the expression of neighboring genes, we sought to gain more insights into the underlying mechanisms. Although hnRNPL is well known for modulating RNA splicing, its direct involvement in transcription has been revealed. Of note, it can associate with KMT3A to regulate H3K36me3 enrichment at exons^43^ or impact on transcription elongation through interacting with P-TEFB members, CDK9 and CCNT1^44^. We thus speculated that eRNAs may bind and tether hnRNPL to the target promoter to enhance transcription by increasing local concentration of Pol II or H3K36me3. To test this notion, we found that indeed a high enrichment of hnRNPL was detected on chromatins isolated from C2C12 MTs and it was markedly reduced by the treatment of RNase (Fig. 6a), supporting the idea that hnRNPL association with chromatins was RNA dependent. The functional regulation of seRNA-hnRNPL association was further dissected at the *seRNA-1* locus. First, we performed chromatin isolation by RNA purification (ChIRP) assay^45^ and confirmed *seRNA-1* bound directly to *Mb* promoter but not on the promoters of *Atp1a1*, *MyoG* or *MyHC* (Fig. 6b, Supplementary Fig. 11a). Second, ChIP-PCR results suggested hnRNPL was associated with *Mb* locus (promoter and genic regions) and its binding was elevated in MTs compared to MBs (Fig. 6c), accompanied by the concomitant increase of Pol II, CDK9, CCNT1, KMT3a, and H3K36me3 binding (Fig. 6c), in line with the induction of *Mb* RNA expression. In contrast, the concordant increase of their binding was not seen across *Apol6* locus; the levels of hnRNPL, CDK9 and KMT3a binding were increased in MTs vs MBs (Supplementary Fig. 11b). In addition, we demonstrated that hnRNPL physically interacted with CCNT1 and CDK9 by co-immunoprecipitation (Co-IP) assays (Fig. 6d). The above results together supported the notion *seRNA-1*/hnRNPL could bind with Mb locus and regulate its transcription. Consistently, knockdown of hnRNPL decreased the enrichment of Pol II and H3K36me3 at the *Mb* region (Fig. 6e). *seRNA-1* knockdown, also led to a reduction of H3K36me3 across *Mb* locus (Fig. 6f). Surprisingly, an unexpected increase of hnRNPL binding was observed in the cells (Fig. 6f). Consistently, deletion of CAAA tract also led to the increased deposition of hnRNPL on *Mb* locus (Fig. 6g) despite unaltered hnRNPL level (Supplementary Fig. 8h). Interestingly, hnRNPL binding was unchanged in another *seRNA-1* mutant cell in which a short sequence of second exon was removed (Supplementary Fig. 11c-e). The paradox raised one interesting possibility that instead of guiding hnRNPL binding to chromatins, *seRNA-1* more likely functions to prevent overloading of hnRNPL, which could be detrimental for transcription. Indeed, when hnRNPL was overexpressed, the expression of *Mb* and *seRNA-1*, but not *Apol6*, was reduced (Fig. 6h). Consistently, *Mb* promoter activity was repressed upon hnRNPL ectopic overexpression (Supplementary Fig. 11f), suggesting the amount of hnRNPL was critical for *Mb* transcription. To further test this notion, we found tethering *seRNA-1* RNA to the *seRNA-1* or *Mb* promoter (Fig. 6i) through CRISPR-mediated genome editing^46^ both led to increased hnRNPL deposition on the *Mb* locus thus the reduced level of *Mb* transcription (Fig. 6j, k). These effects were however alleviated if the mutant with CAAA deletion was tethered to *seRNA-1* or *Mb* promoter, strengthening the importance of CAAA tract in regulating hnRNPL binding and *Mb* expression (Fig. 6j, k). In addition, we found that the increased recruitment of hnRNPL upon *seRNA-1* knockdown interestingly led to a reduction of CCNT1 but an increase in CDK9 binding across *Mb* locus (Fig. 6f), while the overall binding among hnRNPL, CCNT1 and CDK9 was not affected (Supplementary Fig. 11g), implying complex and unknown aspects of interactions among hnRNPL, CCNT1 and CDK9.

## Discussion

Findings from this study uncover a mechanism of eRNA action in transcriptional regulation through association with hnRNPL. Using myoblast differentiation as a paradigm and *seRNA-1* as an example, we dissected how it activated the transcription of target gene *Mb* through interacting with hnRNPL and modulating hnRNPL, Pol II, H3K36me3 at *Mb* locus (Supplementary Fig. 12 and Supplementary Note 3). Our findings suggest eRNAs binding to hnRNPL conveys a localized activity profile to the target chromatin, which is in agreement with several recent findings^18,19,47^. For instance, CBP interacts with RNAs generated proximal to CBP chromatin binding and RNA binding in turn stimulates core acetyltransferase activity of CBP at both enhancers and target promoters, followed by gene activation^19^. Nevertheless, different from CBP/eRNAs association, which does not rely on a particular RNA sequence, hnRNPL represents a paradigm where the interaction is mediated by a preferred RNA binding motif. Our results identified eight tandem-repeats of CAAA tract in the 5′–300 nt region that was essential for both hnRNPL binding and *seRNA-1*-dependent activation of target genes in vitro and in vivo (Fig. 5). Thus, the locus specificity of hnRNPL may largely depend on RNA sequence, conferring a robust and accurate local regulation in gene transcription.

HnRNPL is well known as a regulator of RNA alternative splicing^41,42,48^, our findings, however, demonstrate its involvement in transcriptional control. This is in accordance with a few existing reports. In a separate study, Li et al. showed that in THP-1 macrophage, lncRNA *THRIL* interacted with hnRNPL to activate TNFα expression^49^ while *lincRNA-EPS* interacted with hnRNPL to repress the expression of immune genes in macrophages^50^, suggesting the diverse functional modes of hnRNPL/lncRNA interactions. It appeared that hnRNPL/eRNA interaction may generally occur in many cells since transcriptome-wide CLIP-seq analyses in myotube and HeLa suggested hnRNPL bound to eRNAs globally (Supplementary Figs. 9, 10); and the binding may in turn modulate the transcriptional output of target gene. Indeed, emerging studies of RNA binding proteins (RBPs) have revealed that traditional splicing factors may possess a wide range of functions. For example, Lubelsky et al. recently demonstrated that hnRNPK binds with lncRNAs to facilitate their nuclear retention^51^, which was further reinforced by a report showing *U1* snRNP spliceosome was also important for the chromatin tethering of lncRNAs^52,53^.

In addition to defining an eRNA mechanism, we also provided a categorization of eRNAs by comparing GRO-seq, total RNA-seq and PolyA^+^ RNA-seq datasets. Through analyzing features associated with these types of eRNAs, we found Bi-stable pairs were linked to highest expression level, TFs occupancy and active histone marks. Nevertheless, we must point out that our definition of stable/unstable was simply based on the presence of an eRNA in GRO-seq vs. steady state RNA-seq. In the future, it will be interesting to continue to explore eRNA turnovers and to investigate the determinants of their biogenesis and subcellular localizations. For instance, it will be interesting to study whether intrinsic features on the eRNA sequences such as the presence of early polyadenylation sites (PASs) and *U1* splicing signals also determine eRNA stability^49^ and to find out whether transcriptional activation is correlated with stability of eRNAs (Supplementary Fig. 2).

Our results uncovered the key role of MyoD in regulating enhancer transcription thus reinforcing MyoD function in enhancer assembly and activation^6,27^. In this study, our analyses in both *MyoD*^−/−^ and WT C2C12 cells revealed that MyoD accounted for eRNAs induction in MTs, which agreed with its established roles in enhancer activation (Fig. 2). Surprisingly, ablation of *MyoD* induced eRNAs expression in MBs, which appeard to imply a repressive role of MyoD in enhancer transcription at this stage. Among the genes enriched upon *MyoD* loss, we found some related to nervous system development which is in line with a report showing MyoD expression prevented the neuronal differentiation^54^. Therefore, it is tempting to speculate that MyoD not only primes enhancers important for myogenic lineage but also suppresses neuronal genes in MBs. However, we cannot rule out the possibility that some other repressive factors bind with MyoD to inhibit the gene expression.

In summary, our findings highlight a key role for hnRNPL in *seRNA-1* mediated transcriptional regulation via CAAA binding, providing insights into the mechanism for eRNAs regulation of target transcription.

## Methods

### Animal studies

All animal experiments were performed following the guidelines for experimentation with laboratory animals set in the Chinese University of Hong Kong and approved by the Animal Experimentation Ethics Committee of the Chinese University of Hong Kong. The mice were maintained in animal room with 12 h light/12 h dark cycles at Animal Facility in CUHK. For Cardiotoxin (CTX) injection, approximately seven-week-old male C57BL/6 mice were injected with 50 μl of CTX at 10 μg ml^−1^ into the tibialis anterior muscles (TA). Mice were sacrificed and TA muscles were harvested at designated days for analysis. For satellite cell sorting, approximately eight-week-old homozygous male Tg:Pax7-nGFP mice were used to isolate MuSCs. For siRNA injection, ~8-week-old male C57BL/6 mice were first injected with 50 μl of 1.2% BaCl_2_ solution into the TA muscles. Oligos were prepared by pre-incubating 2 μM of siRNA oligos (Shanghai GenePharma Corp., China) with Lipofectamine 2000 (Invitrogen) for 15 mins and injections were made in a final volume of 50 μl in OPTI-MEM (Life Technologies, Inc.)^21,23^. The siRNAs were administrated at indicated time points. Mice were killed and TA muscles were harvested at day 7 after BaCl_2_ injection, and total RNAs were extracted for qRT–PCR analyses. For AAV9 injection, homozygous Pax7^Cas9^ mice were injected with AAV9-sgRNA at P10 at a dose of 5 × 10^11^ viral genomes (vg) per animal by intramuscular (i.m) injection. Animals were sacrificed and quadriceps muscles were isolated 4 weeks post AAV injection. Genomic DNA and RNA were then extracted and subjected to genotyping and qRT-PCR analysis respectively. A total of three mice were used per group.

### Cell culture

Mouse C2C12 myoblast cells (CRL-1772) were obtained from ATCC and maintained in DMEM supplemented with 10% FBS, 100 U ml^−1^ penicillin and 100 μg of streptomycin (growth medium, GM) in a 5% CO_2_ humidified incubator at 37 °C. For myogenic differentiation, cells cultured in 60 mm or 100 mm plates were shifted to DMEM containing 2% horse serum (differentiation medium, DM) when the confluence reached 80–90%. C3H/10T1/2 fibroblast cells (ATCC, CCL-226) were cultured in DMEM supplemented with 10% FBS, 2 mM l-glutamine, 100 U ml^−1^ penicillin and 100 μg of streptomycin in a 5% CO_2_ humidified incubator at 37 °C. The cells were induced to differentiation after transfection with MyoD expressing vector or empty vector control by switching to DMEM containing 2% horse serum. 293T cells (ATCC, CRL-3216) were cultured in DMEM supplemented with 10% FBS, 2 mM L-glutamine, 100 U ml^−1^ penicillin and 100 μg of streptomycin in a 5% CO2 humidified incubator at 37 °C. Muscle satellite cells (SCs) were sorted by FACS based on established methods^55^. Briefly, hindlimb muscles from Tg:Pax7-nGFP mice were collected and digested with collagenase II (800 U ml^−1^, Worthington) for 90 min at 37 °C, and then the digested muscles were triturated and washed in washing medium (Hams F-10 media (Sigma), 10% HIHS (Gibco), Penicillin/streptomycin (1x, Gibco)) before SCs were liberated by treating with Collagenase II (800 U ml^−1^) and Dispase (11 U ml^−1^) for 30 min at 37 °C. Mononuclear cells were filtered with a 70-µm cell strainer and GFP^+^ SCs were sorted out by BD FACSAria Fusion cell sorter (BD Biosciences) following the manufacturer’s instructions. SCs were cultured in F10 medium (Sigma) with 20% FBS, penicillin/streptomycin (1x) and 2.5 ng/ml basic fibroblast growth factor (bFGF, 13256, Life Technologies). SCs were harvested at designated days, and total RNAs were extracted for qRT-PCR analysis.

### Cell fractionation

C2C12 cells at MB or MT stage were collected in cold PBS, washed twice and then incubated in buffer A (HEPES-KOH 50 mM pH 7.5, 10 mM KCl, 350 mM sucrose, 1 mM EDTA, 1 mM DTT, 0.1% Triton X-100) for 10 min on ice with occasional shaking. The nuclei were harvested by brief centrifugation (2000 g, 5 min). The supernatant was collected as the cytoplasmic fraction. The nuclei were further washed twice more with buffer A without Triton X-100. RNA was extracted using TRIzol reagent and cDNAs were prepared as usual. We used 1 μg of RNA for qRT-PCR analysis of eRNAs, U1, Xist, Malat1 and Gapdh. Based on the total recovery amount of each potion (cytoplasmic or nuclear), we calculated the enrichment of nuclear and cytoplasmic RNA. Sequences of primers used are listed in Supplementary Data 6.

### Oligonucleotides and transfections

siRNA against seRNAs, hnRNPL or control oligos were obtained from Shanghai GenePharma Corp., China. In each case, the concentration used for transient transfections was 100 nM. Transient transfection of cells with siRNA oligos or DNA constructs was performed on 60 or 100 mm dishes or six-well plate with Lipofectamine 2000 reagent (Invitrogen) according to the manufacturer’s protocol. Sequences of siRNA oligos are listed in Supplementary Data 6.

### Plasmid

The MyoD expression vector was a kind gift from Prof. Zhenguo Wu (Hong Kong University of Science and Technology, HKUST). Myc-tagged hnRNP L deletion mutants plasmids were kind gifts from Prof. Gang Wang (Shanghai Institute of Biochemistry and Cell Biology, Chinese Academy of Science)^56^. AAV9 serotype plasmid and pDF6 were kind gifts from Prof. Bin Zhou (Shanghai Institute of Biochemistry and Cell Biology, Chinese Academy of Science). For overexpression of seRNAs, full-length sequence of seRNA-1 or seRNA-2 was PCR-amplified and cloned into the BamHI and XhoI sites of pcDNA3.1 (Invitrogen). For RNA pull-down assays, full-length or truncated fragments of seRNAs were PCR-amplified and cloned into the BamHI and XhoI sites of pBluescript KS (+) vector^21^. For seRNA promoter luciferase reporter construct, a 313 bp sequence flanking seRNA-1 TSS site or a 332 bp sequence flanking seRNA-2 TSS site that encompasses the MyoD binding site was amplified from C2C12 genomic DNA and cloned into KpnI and NheI sites of pGL3-Basic Vector (Promega). Sequences of primers used are listed in Supplementary Data 6.

### Luciferase reporter assay

Cells were transfected with indicated luciferase reporter plasmids (200 ng) and 10 ng of Renilla plasmid using Lipofectamine 2000 (Life Technologies) in a 24-well format. At 24 h post-transfection, cells were differentiated for 48 h and the luciferase activity was measured using the Dual-Glo Luciferase Assay system (Promega) according to the manufacturer’s guidelines. The Luciferase/Renilla ratio was calculated for all samples. Measurement was performed in triplicate biological samples.

### Generation of stable knock-down cell lines

Small hairpin RNAs (shRNAs) for seRNA-1, seRNA-2, and hnRNPL knockdown were designed, synthesized and cloned into pSIREN vectors (Clontech). To generate C2C12 cells with stable knocking down of seRNAs or hnRNPL, 2.5 µg empty pSIREN-RetroQ retroviral vector, pSIREN/seRNA or pSIREN/hnRNPL along with 2.5 µg packaging plasmid were co-transfected into HEK293T cells in a 6 cm dish, respectively. Forty-eight hours after transfection, culture supernatants were collected, filtered and used for infecting C2C12 cells, followed by puromycin selection (2.5 μg ml^−1^) for three days. The cells were then cultured in medium without puromycin for another 3 days. The remaining cells were collected for analysis. All shRNA sequences are provided in Supplementary Data 6.

### Genomic editing by CRISPR-Cas9 in cells

MyoD^−/−^ C2C12 cells were generated previously^6^. To delete CAAA tract or a short sequence in exon 2 in seRNA-1 locus in C2C12 cell, target-specific guide RNAs (gRNAs) were selected using the CRISPR design tool (http://crispr.mit.edu/) and then cloned into pX330 plasmid (Addgene, 42230)^57^. C2C12 cells were transfected with two constructed plasmids (2.5 μg each) using Lipofectamine 2000 (Life Technologies). An empty pSIREN-RetroQ plasmid (Clontech) was co-transfected for screening. Forty-eight hours after transfection, the cells were selected with 2.5 μg ml^−1^ puromycin for 3 days and cultured in medium for another 3 days without puromycin. Cells were then diluted into 96-well plates to get single-cell clones. Individual colonies were picked, PCR validated and subjected to Sanger DNA sequencing. Sequences of all the gRNAs and genotyping PCR primers are provided in Supplementary Data 6.

### seRNA-1 tethering

The full-length of seRNA-1 RNA or CAAA tract deletion mutant was fused to sgRNAs targeting to the seRNA-1 TSS or Mb promoter at its 5′ end and a U1 3′box at its 3′end. The RNA-sgRNA fusion constructs were then transfected with dCas9 into C2C12 cells. At 24 h post-transfection, C2C12 cells were differentiated for 48 h before harvesting for RT-qPCR or ChIP-PCR analysis. Sequences for sgRNAs are provided in Supplementary Data 6.

### ChIP-PCR

Approximately 1 × 10^7^ C2C12 cells were crosslinked with 1% formaldehyde for 10 minutes at room temperature and quenched by 125 mM Glycine. Cells were washed and collected by centrifugation at 700 g for 5 min at 4 °C, flash-frozen in liquid nitrogen and stored at −80 °C. Cells were lysed in 10 ml of LB1 (50 mM HEPES-KOH pH 7.5, 140 mM NaCl, 1 mM EDTA, 0.25% Triton X-100, 0.5% NP-40, 10% glycerol, supplemented with cOmplete protease inhibitors (Roche)) and incubated on a rotator at 4 °C for 10 min. Following centrifugation at 1,350 g for 5 min at 4 °C, the pellets were washed with 10 ml of LB2 (10 mM Tris-HCl pH 8.0, 200 mM NaCl, 1 mM EDTA pH 8.0, 1 mM EGTA pH 8.0, supplemented with cOmplete proteinase inhibitors) by incubating them on a rotator at 4 °C for 10 min. Following centrifugation at 1,350 g for 5 min at 4 °C, nuclei were rinsed twice with 2 ml of sonication buffer (10 mM Tris-HCl pH 8.0, 0.1% SDS, 1 mM EDTA, supplemented with cOmplete proteinase inhibitors). Then, the nuclei were resuspended in sonication buffer and sonicated with Covaris S220 (Intensity 140 W, Duty Cycle 5%, Cycles per Burst 200, Time 7 mins). The resulting lysate was supplied with NaCl and Triton X100 to reach a final concentration at 150 mM NaCl and 1% Triton X100 and then cleared by centrifugation for 20 min at 20,000 g and then incubated with washed Dynabeads Protein G (Invitrogen, 10004D) for 2 h at 4 °C. The cleared lysate was incubated with 2 μg MyoD (sc-304x, Santa Cruz), hnRNPL (sc-28726, Santa Cruz), Pol II (sc-899, Santa Cruz), H3K36me3 (ab9050, abcam), CDK9 (sc-484, Santa Cruz), CCNT1 (sc-10750, Santa Cruz) or IgG control (sc-2027, Santa Cruz) for overnight at 4 °C. On the next day, 30 μl Dynabeads Protein G was washed with IP buffer (10 mM Tris-HCl pH 8.0, 150 mM NaCl, 0.1% SDS, 1 mM EDTA, 1% Triton X100, supplemented with cOmplete proteinase inhibitors) and then applied to each IP reaction followed by incubation at 4 °C for another 2 h. Beads were then collected and washed twice with IP buffer, two times with high-salt wash buffer (10 mM Tris-HCl pH 8.0, 500 mM NaCl, 0.1% SDS, 1 mM EDTA, 1% Triton X100, supplemented with cOmplete proteinase inhibitors), two times with LiCl wash buffer (10 mM Tris-HCl pH 8, 250 mM LiCl, 1 mM EDTA, 0.5% NP-40, 0.5% deoxycholate, supplemented with cOmplete proteinase inhibitors) and one time with cold TE buffer (10 mM Tris-HCl pH8.0, 1 mM EDTA, 50 mM NaCl). Immunocomplexes were eluted in ChIP elution buffer (50 mM Tris-HCl pH8.0, 10 mM EDTA, 1% SDS) with incubation at 65 °C for 30 min and eluents were reverse crosslinked by incubating at 65 °C for 16 h. Immunoprecipitated DNA was treated with RNase A (0.2 mg ml^−1^) at 37 °C for 2 h, followed by Proteinase K treatment (0.2 mg ml^−1^) at 55 °C for 3 h. Immunoprecipitated DNA was then subjected to phenol:chloroform extraction and ethanol precipitation and finally resuspended in 1x TE buffer. qPCR was done with Power SYBR (Applied Biosystems) using primers for target genomic locus. ChIP-PCR primers are listed in Supplementary Data 6. Percentage of input recovery was calculated.

### Chromatin isolation by RNA purification

Biotin-labeled antisense oligos (20 nucleotides long) were designed targeting seRNA-1 and divided into odd and even pools. Differentiating C2C12 cells (DM D3) were harvested and crosslinked with 1% glutaraldehyde and an amount of 100 mg of cell pellet were used for one pull down. Cells were lysed with lysis buffer (50 mM Tris-HCl pH 7.0, 10 mM EDTA, 1% SDS supplemented with cOmplete protease inhibitors and SUPERase In) and sonicated at 4 °C with Covaris sonicator S220 to shear the DNA to 100–500 bp. Pooled odd and even probes were hybridized with sonicated chromatins in hybridization buffer (750 mM NaCl, 1% SDS, 50 mM Tris-HCl pH 7.0, 1 mM EDTA, 15% formamide supplemented with cOmplete protease inhibitors and SUPERase In) and then pulled down by Dynabeads MyOne Streptavidin C1 (65001, Invitrogen). Retrieved RNA and DNA were isolated respectively. Isolated RNA was reverse-transcribed and analyzed by qRT-PCR to evaluate the pull-down efficiency. qRT-PCR was conducted with retrieved DNA using the primers to check the enrichment of seRNA-1 at certain genomic locus. Sequences of all Chromatin isolation by RNA purification (ChIRP) probes and ChIRP-PCR primers are listed in Supplementary Data 6.

### RT-PCR and Real-time RT-PCR

Total RNAs from tissues or cells were extracted using TRIzol reagent (Invitogen). cDNAs were prepared using M-MLV or Superscript III (Thermo Fisher Scientific) reverse transcriptase and Oligo(dT) 20 primers or random hexamer primers (Thermo Fisher Scientific). Expression of mRNA was determined with SYBR Green Master Mix (4309155, Thermo Fisher Scientific) in a LightCycler® 480 Instrument II (Roche Life Science). Gapdh and 18 s were used for normalization. Sequences of all primers used are listed in Supplementary Data 6.

### Rapid amplification of cDNA ends

SMARTer^TM^ Rapid amplification of cDNA Ends (RACE) cDNA Amplification Kit (Clontech) was used following the manufacturer’s instructions. In brief, to prepare 5′ RACE-Ready cDNAs, 1 μg of total RNAs extracted from C2C12 cells at DM D3 were reverse transcribed using 5′-CDS Primer A, SMARTer IIA oligo and SMARTScribe Reverse Transcriptase provided in the kit. Subsequently, PCR amplification step was performed using a gene-specific reverse primer (RP) designed to target the seRNA candidates and a Universal Primer Mixture (UPM) from the kit. Similarly, 3′ RACE-Ready cDNAs were generated by using 3′-CDS Primer A. The following PCR amplification was done using gene-specific primer together with a Universal Primer Mixture (UPM) from the kit. The sequences of gene-specific primers are listed in Supplementary Data 6.

### RNA pull-down assay

In brief, the DNA constructs were first linearized by single enzyme digestion (BamHI for antisense transcript or XhoI for sense transcript and fragmented transcripts). The resulting linearized constructs were utilized to generate the biotinylated RNAs through in vitro transcription using MAXIscript T7/T3 In vitro transcription kit (Ambion) and Biotin RNA labeling Mix (Roche). The above RNAs were denatured at 90 °C for 2 min, immediately transferred on the ice for 3 min and then supplemented with RNA structure buffer (Ambion) followed by the renaturation step performed at room temperature (RT) for 20 min. Nuclear proteins or total proteins were collected for the RNA pull-down assay. For nuclear protein extraction, 2 × 10^7^ MT C2C12 cells (DM D3) were harvested and incubated with nuclear isolation buffer (1.28 M sucrose; 40 mM Tris-HCl pH 7.5; 20 mM MgCl_2_; 4% Triton X-100) supplemented with cOmplete protease inhibitors (Roche). Nuclei were collected by centrifuge at 2,500 g at 4 °C for 15 min. Nuclear pellet was resuspended in 1 ml RIP buffer (150 mM KCl, 25 mM Tris-HCl pH 7.4, 0.5 mM DTT, 0.5% NP40, 1 mM PMSF, supplemented with cOmplete protease inhibitors and 100 U ml^−1^ RNaseOUT) and homogenized for eight cycles using an Ika homogenizer (Ika-Werk Instruments, Cincinnati). After centrifugation at 16,200 g for 10 min to remove nuclear membrane and debris, 1 mg of C2C12 nuclear extracts were then incubated with 3 μg of renatured RNA at RT for 1 hr. Then, 30 μl pre-washed Dynabeads M-280 Streptavidin (11205D, Invitrogen) were added to each reaction and incubated at RT for an extra 1 hr. Beads were collected using a magnetic rack followed by washes for 5 times using RIP buffer. The resulting beads were boiled for 5 min in western blotting loading buffer to retrieve the proteins and then detected by standard western blotting. For total protein extraction, 4 × 10^6^ C2C12 cells (DM D3) were harvested and directly incubated with RIPA buffer (50 mM Tris-HCl, pH 7.4, 150 mM NaCl, 5 mM EDTA, 1% NP-40, 1% sodium deoxycholate, 0.1% SDS) supplemented with cOmplete protease inhibitors and RNaseOUT (100 U ml^−1^) for 30 min on ice with occasional shaking. The total proteins were recovered by centrifuge at 16,200 g for 10 min at 4 ^o^C. Then the following pull down procedure was performed as mentioned above except replacing the RIP wash buffer with RIPA buffer.

### Native RNA immunoprecipitation (RIP) assay

Briefly, 8 × 10^6^ C2C12 cells (DM D3) were harvested and resuspended in 100 μl ice-cold Polysomal Lysis Buffer (100 mM KCl, 5 mM MgCl_2_, 10 mM HEPES-KOH pH 7.0, 0.5% NP-40, 1 mM DTT, 100 U ml^−1^ RNaseOUT, 400 μM Vanadyl ribonucleoside complexes (VRC) and cOmplete protease inhibitors). The lysate were incubated on ice for 5 min followed by centrifugation at 15,000 g for 15 min to clear lysate of large debris. Fifty microliter of the Dynabeads Protein G (Invitrogen, 10004D) for each reaction were prepared through three-times washes with ice-cold NT2 buffer (50 mM Tris-HCl pH 7.4, 150 mM NaCl, 1 mM MgCl_2_ and 0.05% NP40). Then, 5 μg of each antibody were added into the prepared Dynabeads Protein G in NT2 buffer and incubated at room temperature for 40 min. Immediately before use, wash with 500 μl of ice-cold NT2 buffer for five times. After the final wash, the antibody-coated beads were resuspended in 850 μl of ice-cold NT2 buffer supplemented with 200 units RNaseOut, 400 μM VRC, 10 μl of 100 mM DTT and EDTA to 20 mM. Following this, 100 μl of cleared cell lysate were added to the antibody/Dynabeads Protein G mixture and incubated for overnight. Meanwhile, 10 μl of the cell lysate was saved as “10% Input” for subsequent RNA analysis. On the next day, the protein/antibody/Dynabeads Protein G complexes were washed intensely with 1 ml of ice-cold NT2 buffer for five times and resuspended in 100 μl of ice-cold NT2 buffer and saved 10 μl for protein analysis to test the immunoprecipitation efficiency. Then, the complexes were treated with proteinase K at 55 ^o^C for 30 min with interval shaking. After incubation, 1 ml Trizol reagent was added and the RNA was extracted according to the manufacturer’s instructions. The cDNAs were prepared using Superscript III reverse transcriptase (Life Technologies) and random hexamer primers (SO142, Thermo Fisher scientific). Antibodies against hnRNPL (sc-28726), hnRNPK (sc-25373) and normal IgG (sc-2027) were from Santa Cruz Biotechnology.

### GRO-seq

Five million cells (WT C2C12 cells or MyoD KO cells at MB and MT stages) were resuspended in cold swelling buffer (10 mM Tris-HCl pH 7.5, 2 mM MgCl_2_, 3 mM CaCl_2_) on ice for 5 min and then lysed in lysis buffer (swelling buffer + 0.5% IGEPAL + 10% glycerol + 2 U ml^−1^ SUPERase In and cOmplete protease inhibitors) followed by gently pipetting up and down for 20 times. After centrifuge, nuclei were sequentially washed with lysis buffer and freezing buffer (50 mM Tris-HCl pH 8.3, 40% glycerol, 5 mM MgCl_2_, 0.1 mM EDTA) and finally resuspended in 100 µl of freezing buffer and stored at −80 °C. Nuclear run-on (NRO) assays were performed with biotin-11-UTP as previously reported. In brief, 2 × NRO master mix (10 mM Tris-HCl pH 8.0, 5 mM MgCl_2_, 1 mM DTT, 300 mM KCl, 1% Sarkosyl, 250 µM ATP, GTP, CTP, 50 µM biotin-11-UTP, and 0.8 U µl^−1^ SUPERase In) was pre-equilibrated at 37 °C for 10 min. Then, 5 × 10^6^ cells in 100 µl nuclei were added to the same volume 100 µl of 2 × NRO master mix and incubated at 37 °C for 5 minutes. The run-on RNA (NRO-RNA) was extracted with 2 ml of TRIzol reagent (Invitogen) following the manufacturer’s instructions and then fragmented with 5 μl of ice-cold 1 N NaOH for 10 minutes on ice. The reaction was neutralized by mixing with 25 μl of 1 M Tris-HCl pH 6.8 and fragmented biotinylated RNA was purified through incubation with 30 µl of Dynabeads™ M-280 Streptavidin beads (Thermo Fisher Scientific) in binding buffer (10 mM Tris-HCl pH 7.4, 300 mM NaCl and 0.1% Triton X-100) for 20 min at room temperature while rotating. Then, the beads were sequentially washed two times with high-salt wash buffer (50 mM Tris-HCl pH 7.4, 2 M NaCl and 0.5% Triton X-100), two times with binding buffer, and one timer with low-salt wash buffer (5 mM Tris-HCl pH 7.4, 0.1% Triton X-100). After elution from beads, biotinylated RNA was subject to 3′ RNA adaptor ligation and incubated in a 10 µl reaction volume containing 50 pmol of 3′ RNA adaptor (5’p-rGrArUrCrGrUrCrGrGrArCrUrGrUrArGrArArCrUrCrUrGrArArC-/3′InvdT/) (Integrated DNA Technologies, IDT), 10 nmol of ATP, 10 unites of T4 RNA ligase I (NEB), 40 unites SUPERase In and 10% PEG 8000 at 20 °C for 6 h. Ligated RNA was enriched by Streptavidin beads and RNA extraction with TRIzol. The 5′ ends of biotinylated RNA were then repaired with RNA 5′ Pyrophosphohydrolase (RppH) (NEB) and T4 polynucleotide kinase (PNK) (NEB) followed by Trizol extraction. Subsequently, the purified RNA was ligated to 5′ RNA adaptor (5′-rCrCrUrUrGrGrCrArCrCrCrGrArGrArArUrUrCrCrA-3′) (IDT) in a 10 µl reaction volume containing 50 pmol of 5′ RNA adaptor, 10 nmol of ATP, 10 unites of T4 RNA ligase I (NEB), 40 unites SUPERase In and 10% PEG 8000 at 20 °C for 6 h. Following the ligation, ligated RNA was purified by the third round Streptavidin bead enrichment and TRIzol extraction. The resultant RNA was reverse transcribed with RP1 primer (5′-AATGATACGGCGACCACCGAGATCTACACGTTCAGAGTTCTACAGTCCGA-3′) (IDT) using Superscript III RT enzyme (Thermo Fisher Scientific). A small portion of cDNA was serial diluted and subjected to test PCR amplification to determine optical PCR cycle. The full-scale PCR amplification was performed using Q5^®^ High-Fidelity 2 × Master Mix (NEB) with 12.5 pmol of RP1 primer and RPI-index primers (5′ -CAAGCAGAAGACGGCATACGAGATNNNNNNGTGACTGGAGTTCCTTGGCACCCGAGAATTCCA-3′, in which “NNNNNN” is an index sequence) (IDT). The optimal PCR cycle for full-scale amplification is 14. PCR products were PAGE separated and DNA from 140 to 350 bp was eluted from PAGE gel. Then, the library was quantified and sequenced in the Illumina HiSeq 1500.

### RNA-seq

Total RNAs were extracted from C2C12 cells and subjected to poly(A) selection (Ambion, 61006) followed by library preparation using NEBNext® Ultra™ II RNA Library Preparation Kit (NEB). Libraries with barcodes were pooled at equal concentrations and sequenced on the Illumina HiSeq 1500 platform.

### hnRNPL chromatin binding assay

C2C12 cells were washed with cold PBS and 1/10 was resuspended in RIPA buffer (150 mM Tris-HCl, pH 8.0, 150 mM NaCl, 0.5% DOC, 0.1% SDS, 1% NP-40 and cOmplete protease inhibitors (Roche)) and left for 30 min on ice. The remaining was lysed for 15 min on ice in cold CSKI buffer (10 mM PIPES, pH 6.8, 100 mM NaCl, 1 mM EDTA, 300 mM sucrose, 1 mM MgCl_2_, 1 mM DTT, 0.5% Triton X-100, and cOmplete protease inhibitors (Roche)). The cell lysate was centrifuged at 500 g at 4 °C for 3 min and the pellets was washed twice in CSKI buffer and then resuspended in CSKII buffer (10 mM PIPES, pH 6.8, 50 mM NaCl, 300 mM sucrose, 6 mM MgCl_2_, 1 mM DTT, and cOmplete protease inhibitors (Roche)). Half of the suspension was treated with 1: 100 dilution of RNase A (20 mg ml^−1^, Thermo Fisher Scientific) for 10 min at 37 °C whereas the other was left untreated (control). After washing, both RNase A-treated and untreated nuclei were treated with DNase for 30 min followed by extraction with 250 mM NH_2_SO_4_ for 10 min at 25 °C. The supernatants were collected for western blotting analysis.

### Immunoblotting and immunofluorescence

The total proteins were extracted using RIPA lysis buffer. The following dilutions of antibodies were used for each antibody: anti-MyoG (1:2000, sc-576, Santa Cruz), anti-MyHC (1:2000, M4276, Sigma), anti-hnRNPL (1:5000, sc-28726, Santa Cruz), anti-hnRNPK (1:5000, 4675, Cell Signaling), anti-MED1 (1:5000, A300–793A, Bethyl Laboratories), anti-RAD21 (1:5000, A300–080A, Bethyl Laboratories), anti-RBBP5 (1:5000, A300–109A, Bethyl Laboratories), anti-YY1 (1:2000, sc-1703, Santa Cruz), anti-MyoD (1:2000, sc-760, Santa Cruz), anti-α-Tubulin (1:5000, sc-23948, Santa Cruz), and anti-H3K36me3 (1:5000, ab9050, Abcam). For Immunofluorescence staining of cultured C2C12 cells, the following dilutions were used: anti-MyHC (1:350, M4276, Sigma). All fluorescent images were captured with a Nikon fluorescence microscope.

### Co-immunoprecipitation assay

C2C12 cells (DM D2) were lysed in hypotonic lysis buffer (10 mM HEPES, pH 7.9, 10 mM KCl, 0.1 mM EDTA, 0.1 mM EGTA, and cOmplete protease inhibitors (Roche)) and incubated on ice for 15 min. The lysates were centrifuged for 10 min at 1,530 g and the liquid portion was discarded. The pelleted nuclei were washed once with hypotonic lysis buffer and then resuspended in hypertonic buffer (20 mM HEPES, pH 7.9, 0.4 M NaCl, 1 mM EDTA, 1 mM EGTA, 0.6% NP-40 and cOmplete protease inhibitors (Roche)), digested with the DNase I (AM2238, Thermo Fisher Scientific) for 45 min at 4 °C, and spun down at 13,800 g for 10 min at 4 °C. The nuclear lysates were diluted 2 fold with IP buffer (20 mM HEPES, pH 7.9, 0.2 M NaCl, cOmplete protease inhibitors (Roche) and then precleared by the Protein-G magnetic beads for 1 h with rotation at 4 °C. Then the supernatant were incubated with IgG (sc-2027, Santa Cruz) or specific antibodies (hnRNPL (ARP40368_P050, Aviva Systems Biology), CCNT1 (sc-10750, Santa Cruz)) for overnight with rotation at 4 °C, followed by incubation with Protein-G magnetic beads for 2 h with rotation at 4 °C. The immune-complex were then washed with IP buffer (20 mM HEPES, pH 7.9, 0.2 M NaCl, 0.3% NP-40, cOmplete protease inhibitors (Roche)) for five times. Bound proteins were then eluted in sample buffer (62.5 mM Tris, pH 6.8, 10% Glycerol, 2% SDS, 5% beta-mercaptoethanol, and bromophenol blue) and subjected to western analyses.

### GRO-seq analysis

Low-quality reads were filtered out and adaptor sequences were trimmed from raw reads using Trimmomatic-0.36^58^. The remaining reads were then aligned to the mouse genome (mm9) using Bowtie2 (version 2.2.4). If multiple reads aligned to the same genomic position, only one read per position was kept for downstream analyses. Primary transcripts were de novo identified throughout the genome using HOMER (version v4.4). To define putative eRNAs in MB and MT, according to Zhao et al.^59^, transcripts overlapping with protein-coding genes, antisense transcripts, divergent transcripts and the other genic regions (rRNA, snRNA, miRNA, snoRNA, etc) were filtered and the remaining transcripts were defined as putative eRNAs if their de novo identified transcriptional start site (TSS) was located in super-enhancer or typical-enhancer regions. To determine differentially expressed transcripts, the normalized strand-specific read counts was calculated for each transcript in each GRO-seq experiment using HOMER; EdgeR was then used to determine differential expression (>two-fold changes, FDR < 0.05).

### RNA-seq analysis

The adapter and low-quality sequences were trimmed from 3′ to 5′ ends. Reads shorter than 36 bp were discarded and the remaining reads were mapped to the mouse genome (mm9) using TopHat (v2.0.13). Cufflinks (v2.1.1) was then applied to estimate transcript abundance. Abundance was reported in Fragments Per Kilobase Million (FPKM). Differentially expressed genes were identified if the change of expression level exceeds a fold change threshold (>1.5). As described before^21^, ab initio assembly of the transcriptome using rRNA-depleted RNA-seq data was performed to obtain the novel lncRNAs in MB and MT. To define seRNAs and teRNAs during myogenesis, Genomic coordinates of super-enhancers (SEs) and typical-enhancers (TEs) in MB and MT were retrieved from our previous report^6^. seRNA or teRNA was defined if a lncRNA is originated from SE or TE, respectively.

### ChIP-seq analysis

Briefly, raw reads downloaded from ENCODE and GEO were aligned to the mouse genome (mm9) using Bowtie2 with default parameters. ChIP-seq density (intensity or signal) was calculated as aggregated read counts normalized to the length of the regions and the total mappable read counts, resulting in RPKM (reads per kilo base per million mapped reads) as the measurement unit.

### Data visualization

Visualization of the data for ChIP-seq, GRO-seq and CLIP-seq was performed by organizing custom tracks onto the UCSC genome browser using HOMER software package. The total mappable reads for each experiment were normalized to Reads Per Million (RPM) to facilitate the comparison between different tracks. Meanwhile, seRNAs, super-enhancers, typical-enhancers were also uploaded as tracks on UCSC genome browser for visualization. All heat maps and read density plots were generated by ngsplot^60^ and R package.

### Prediction of RNA secondary structure

Minimum free energy (MFE) structure analysis was carried out using Vienna RNAfold server (http://rna.tbi.univie.ac.at/cgi-bin/RNAfold.cgi).

### Statistical analysis

Data were analyzed using GraphPad Prism (version 8; GraphPad Software, San Diego, CA). Data were represented as mean ± standard deviation (S.D.). All tests were two sided, and *P* *<* 0.05 was considered statistically significant.

### Reporting summary

Further information on research design is available in the Nature Research Reporting Summary linked to this article.

## Supplementary information

Supplementary Information

Peer Review File

Reporting Summary

Description of Additional Supplementary Files

Supplementary Data 1

Supplementary Data 2

Supplementary Data 3

Supplementary Data 4

Supplementary Data 5

Supplementary Data 6

Supplementary Data 7

## Data Availability

A reporting summary for this Article is available as a Supplementary Information file. GRO-seq, hnRNPL CLIP-seq and RNA-seq data reported in this paper were deposited in the Gene Expression Omnibus database under accession GSE114659. All used datasets from other publications and ENCODE project are summarized in Supplementary Data 7. All other data supporting the findings of this study are available from the corresponding author on reasonable request. The source data underlying Figs. 1j, k, 2f–k, 3b–g, 5b–d, f, g and 6a–h, j, k and Supplementary Figs. 3a, c, d, f, 4d, h, i, 6a–f, 7a–g, 7i–n, 8a, c, f–h, j, 9a, b, n, 10g, h and 11a–g, i, j are provided as a Source Data file.

## Source data

Source Data
