# Supplementary material for: MyoD induced enhancer RNA interacts with hnRNPL to activate target gene transcription during myogenic differentiation
Source: Nat Commun. 2019 Dec 19;10:5787. doi: 10.1038/s41467-019-13598-0 (PMC6923398; doi:10.1038/s41467-019-13598-0)
Supplement: Supplementary file 1 — Supplementary Information [file 41467_2019_13598_MOESM1_ESM.pdf]

Supplementary Information

MyoD induced enhancer RNA interacts with hnRNPL to activate target gene transcription during myogenic differentiation

Zhao *et al.* 2019

Supplementary Figures

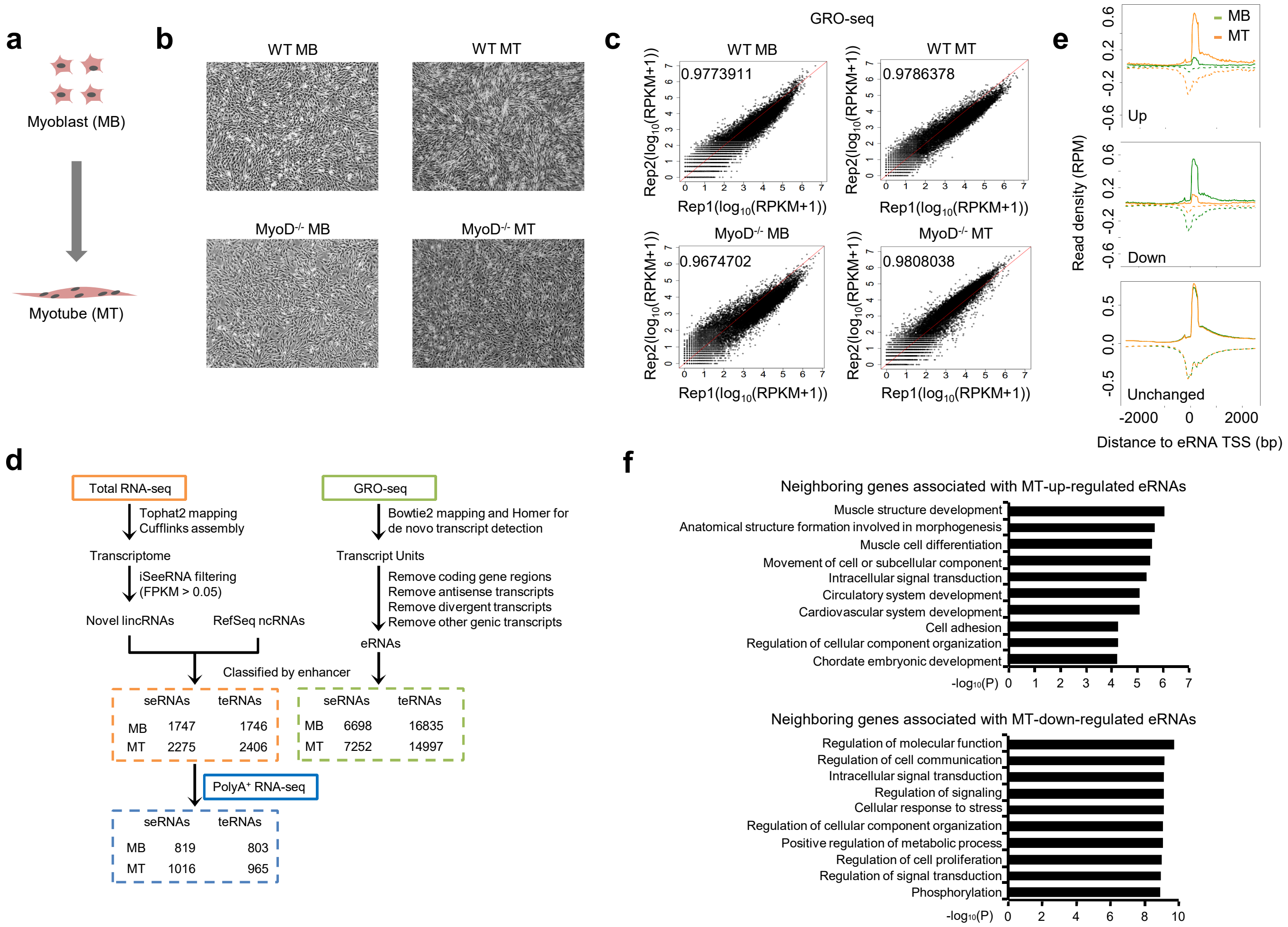

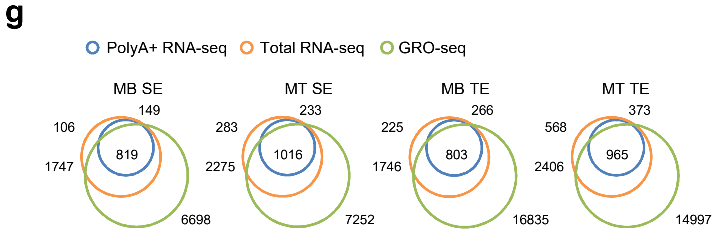

h

	Bi-stable	Bi-stable(%)	Uni-stable	Uni-state(%)	Unstable	Unstable(%)	Total
MB SE	419	6.26	1733	25.87	4546	67.87	6698
MB TE	233	1.38	1803	10.71	14795	87.90	16831
MT SE	431	5.94	2110	29.10	4711	64.96	7252
MT TE	205	1.37	1999	13.33	12790	85.30	14994

Supplementary Figure 1. Elucidation of enhancer transcription in muscle cells. a Schematic illustration of the system used for the study. C2C12 myoblast (MB) cells are differentiated into myotube (MT) cells by culturing in differentiation medium (DM) for 2 days. RNAs from MB or MT cells were collected for GRO-seq analyses. **b** Representative photos of wild type (WT) or MyoD knockout ($MyoD^{-/-}$) C2C12 cells in proliferating MB or differentiating MT (DM D2) state. **c** GRO-seq was performed on above cells. Comparison between two biological replicates is shown. **d** Schematic pipeline for identifying eRNAs in MB and MT cells. **e** GRO-seq detected eRNAs that were up-, down-regulated or unchanged in MT vs MB cells. **f** Gene ontology (GO) analysis of neighboring genes (within ± 150 kb) associated with up- or down-regulated eRNAs in MT vs MB. **g** Comparison of eRNA transcript units identified in super enhancers (SEs) or typical enhancers (TEs) through GRO-seq, total RNA-seq and $PolyA^{+}$ RNA-seq indicates the majority of eRNAs are unstable and can only be detected by GRO-seq. **h** The number and percentage of each type of divergent eRNA pairs, Bi-stable, Uni-stable and Unstable in SEs and TEs.

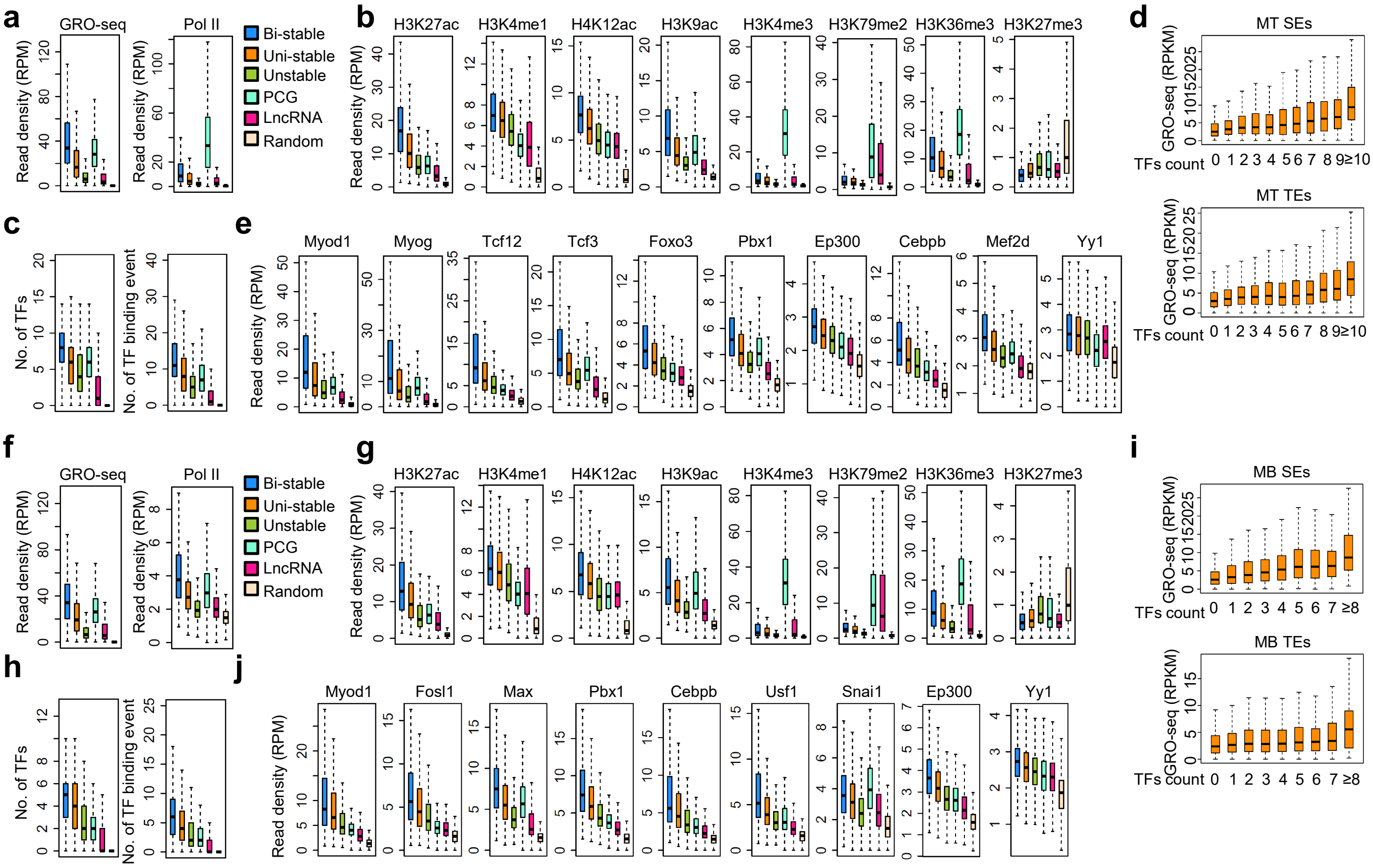

Supplementary Figure 2. Characterization of eRNAs in muscle cells. a-c Box plots comparing the levels of GRO-seq signal and Pol II binding (ChIP-seq) **(a)**, active enhancer marks (e.g. H3K27ac, H3K4me1 and H4K12ac), active transcription marks (e.g. H3K4me3, H3K79me2 and H3K36me3) and repressive mark H3K27me3 **(b)**, and number of TF binding **(c)** associated with MT enhancers generating stable (Bi- or Uni-stable) eRNAs, unstable eRNAs, protein coding gene (PCG), long non-coding RNAs (LncRNA), and random genome background regions. **d** GRO-seq read density on SEs or TEs in MT is positively correlated with the number of binding TFs. **e** Box plots comparing binding density of each TF, associated with MT enhancers generating stable (Bi- or Uni-stable) eRNAs, unstable eRNAs, protein coding gene (PCG), long non-coding RNAs (LncRNA), and random genome background regions. **f-h** Box plots comparing the levels of GRO-seq signal and Pol II binding (ChIP-seq) **(f)**, active enhancer marks (e.g. H3K27ac, H3K4me1 and H4K12ac), active transcription marks (e.g. H3K4me3, H3K79me2 and H3K36me3) and repressive mark H3K27me3 **(g)**, and number of TF binding **(h)** associated with MB enhancers generating stable (Bi- or Uni-stable) eRNAs, unstable eRNAs, protein coding gene (PCG), long non-coding RNAs (LncRNA), and random genome background regions. **i** GRO-seq read density on SEs or TEs is positively correlated with the number of binding TFs in MB. **j** Box plots comparing binding density of each TF, associated with MB enhancers generating stable (Bi- or Uni-stable) eRNAs, unstable eRNAs, protein coding gene (PCG), long non-coding RNAs (LncRNA), and random genome background regions. Data in **a-j** are presented in boxplot. Center line, median; box limits, upper and lower quartiles; whiskers, 1.5× interquartile range.

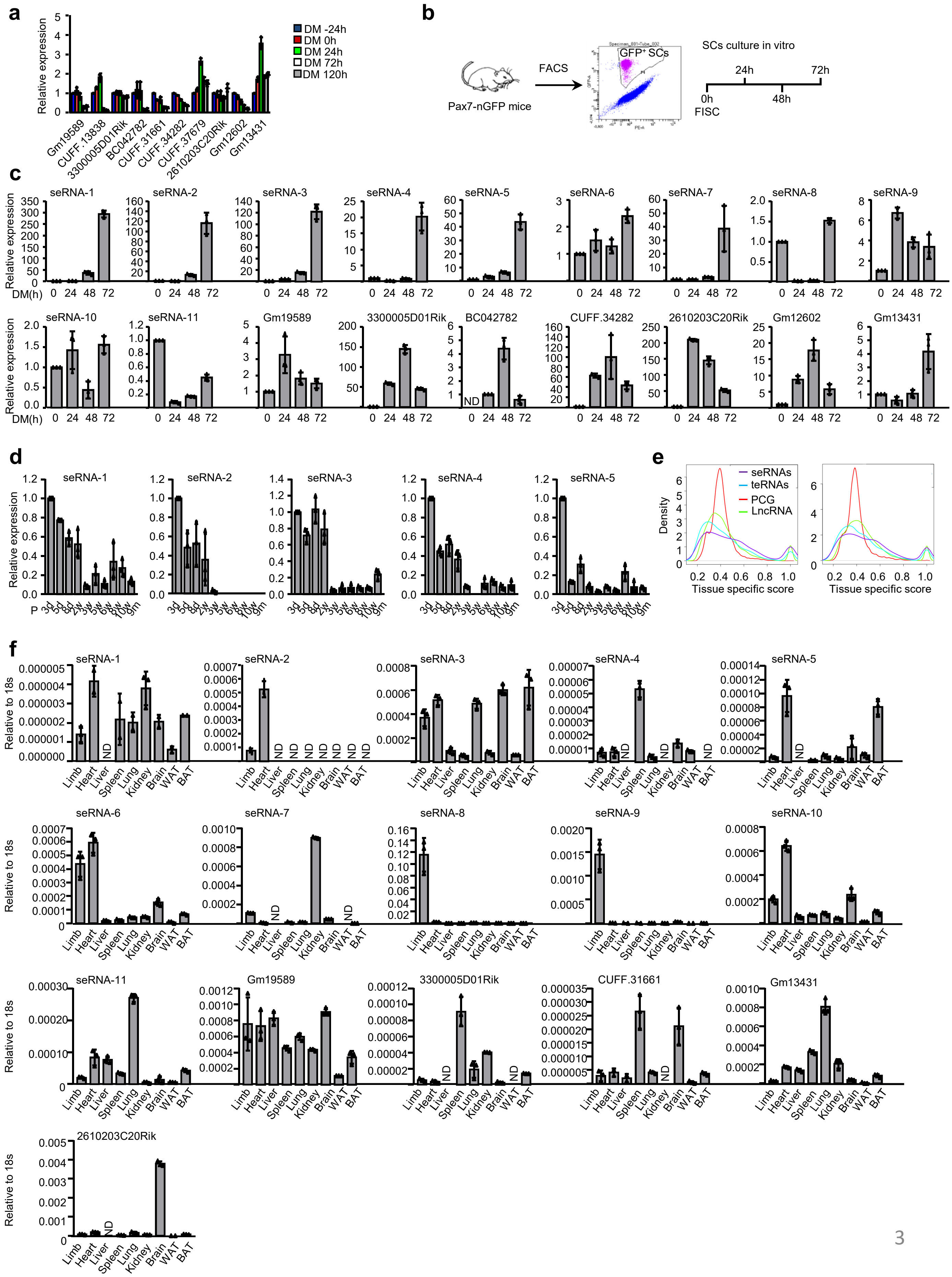
a
Relative expression
DM -24h
DM 0h
DM 24h
DM 72h
DM 120h
Gm19589
CUFF.13838
3300005D01Rik
BC042782
CUFF.31661
CUFF.34282
CUFF.37679
2610203C20Rik
Gm12602
Gm13431
b
Pax7-nGFP mice
FACS
GFP+ SCs
SCs culture in vitro
24h
72h
0h
48h
FISC
c
seRNA-1
seRNA-2
seRNA-3
seRNA-4
seRNA-5
seRNA-6
seRNA-7
seRNA-8
seRNA-9
seRNA-10
seRNA-11
Gm19589
3300005D01Rik
BC042782
CUFF.34282
2610203C20Rik
Gm12602
Gm13431
DM(h)
ND
d
Relative expression
e
seRNAs
teRNAs
PCG
LncRNA
Density
Tissue specific score
f
Relative to 18s
Limb
Heart
Liver
Spleen
Lung
Kidney
Brain
WAT
BAT
CUFF.31661

Supplementary Figure 3. Expression of seRNAs in muscle cells and mouse tissues. a qRT-PCR measurement of expression dynamics of several MB seRNAs during 120 hr differentiation course of C2C12 myoblast in DM. **b.** Schematic outline shows satellite cells (SCs) were isolated by FACS from muscles of Tg: Pax7-nGFP mice. GFP+ cells (purple population) were collected as SC fraction and cultured in vitro in growth medium for 72 hr. Cells were then collected at the indicated time points and qRT-PCR was performed to measure the expression of seRNAs and their target genes. **c** qRT-PCR was performed to measure the expression of seRNAs in the differentiating SCs. **d** RNAs were extracted from postnatal mice at the indicated ages and qRT-PCR analysis was performed to examine the expression of several seRNAs. **e** The distribution of maximal tissue specificity scores calculated for each type of transcripts across 18 different mouse tissues and cells (MB, MT, BAT, bone marrow, BMDM, cerebellum, cortex, heart, kidney, liver, lung, MEF, olfactory bulb, placenta, small intestine, spleen, testis, thymus). Left: MB teRNAs, MB seRNAs, protein coding gene (PCG), long non-coding RNAs (LncRNA); Right: MT teRNAs, MT seRNAs, PCG, LncRNA. **f** qRT-PCR was performed to measure the expression of seRNAs in nine mouse tissues. WAT, white adipose tissue; BAT, brown adipose tissue. Data represent the average of three independent experiments $\pm$ s.d. ND, not detected. Source data are provided as a Source Data file.

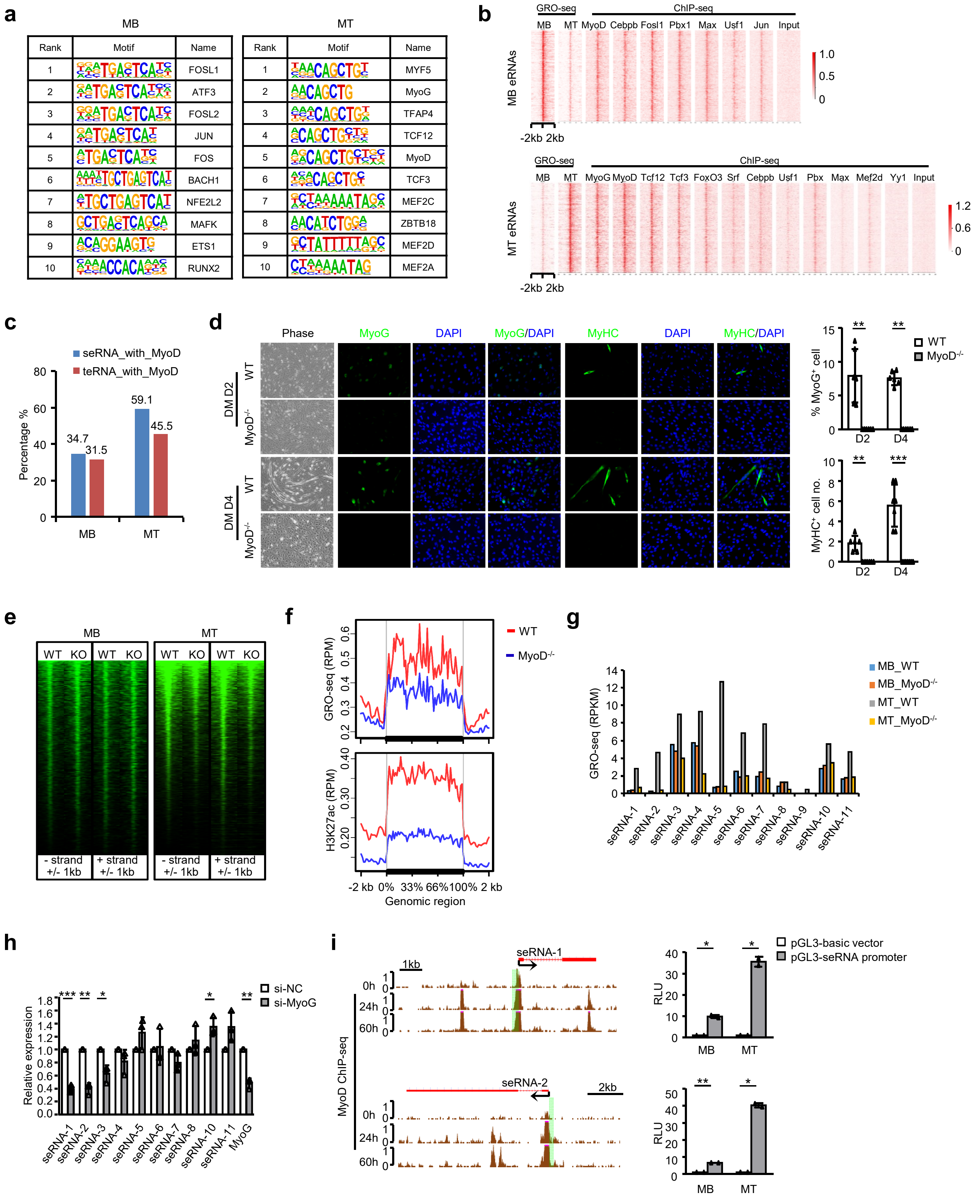

a
MB
Rank
Motif
Name
1 FOSL1
2 ATF3
3 FOSL2
4 JUN
5 FOS
6 BACH1
7 NFE2L2
8 MAFK
9 ETS1
10 RUNX2
MT
Rank
Motif
Name
1 MYF5
2 MyoG
3 TFAP4
4 TCF12
5 MyoD
6 TCF3
7 MEF2C
8 ZBTB18
9 MEF2D
10 MEF2A
b
GRO-seq
ChIP-seq
MB MT MyoD Cebpb Fosl1 Pbx1 Max Usf1 Jun Input
MB eRNAs
1.0
0.5
0
-2kb 2kb
GRO-seq
ChIP-seq
MB MT MyoG MyoD Tcf12 Tcf3 FoxO3 Srf Cebpb Usf1 Pbx Max Mef2d Yy1 Input
MT eRNAs
1.2
0.6
0
-2kb 2kb
c
seRNA_with_MyoD
teRNA_with_MyoD
Percentage %
80
60
40
20
0
34.7
31.5
59.1
45.5
MB
MT
d
Phase
MyoG
DAPI
MyoG/DAPI
MyHC
DAPI
MyHC/DAPI
DM D2
WT
MyoD-/-
DM D4
WT
MyoD-/-
WT
MyoD-/-
% MyoG+ cell
15
10
5
0
**
**
D2
D4
MyHC+ cell no.
10
8
6
4
2
0
**
***
D2
D4
e
MB
MT
WT KO
WT KO
WT KO
WT KO
- strand +/- 1kb
+ strand +/- 1kb
- strand +/- 1kb
+ strand +/- 1kb
f
WT
MyoD-/-
GRO-seq (RPM)
0.6
0.5
0.4
0.3
0.2
H3K27ac (RPM)
0.40
0.30
0.20
-2 kb 0% 33% 66% 100% 2 kb
Genomic region
g
MB_WT
MB_MyoD-/-
MT_WT
MT_MyoD-/-
GRO-seq (RPKM)
14
12
10
8
6
4
2
0
seRNA-1
seRNA-2
seRNA-3
seRNA-4
seRNA-5
seRNA-6
seRNA-7
seRNA-8
seRNA-9
seRNA-10
seRNA-11
h
si-NC
si-MyoG
Relative expression
1.8
1.6
1.4
1.2
1.0
0.8
0.6
0.4
0.2
0.0
***
**
*
*
**
seRNA-1
seRNA-2
seRNA-3
seRNA-4
seRNA-5
seRNA-6
seRNA-7
seRNA-8
seRNA-10
seRNA-11
MyoG
i
1kb
seRNA-1
0h
24h
60h
MyoD ChIP-seq
seRNA-2
2kb
0h
24h
60h
pGL3-basic vector
pGL3-seRNA promoter
RLU
40
30
20
10
0
*
*
MB
MT
RLU
50
40
30
20
10
0
**
*
MB
MT

Supplementary Figure 4. MyoD plays a crucial role in inducing MT eRNAs. a In silico prediction of TF motifs within a ± 1 kb window centered on TSSs of eRNAs in MBs or MTs. **b** ChIP-seq for TFs were collected and analyzed. Heatmap showing TF binding patterns centered at the TSSs of eRNAs in MBs or MTs. The focal red lines at the center demonstrate the co-localization of the indicated TFs. **c** Compared with teRNAs, a larger percentage of seRNAs are occupied by MyoD binding at their TSSs. **d** The degree of differentiation of wild type (WT) or MyoD knockout (MyoD$^{-/-}$) C2C12 cells was measured by IF staining for MyoG and MyHC during a 4-day differentiation course. Myotube formation was visualized in the above cells on day 4 (phase images). IF staining for MyoG and MyHC were performed and the number of positively stained cells per field was quantified by counting > 6 fields per group. **e** Heatmap of GRO-seq showing bi-directional eRNA transcription profiles in the WT or MyoD$^{-/-}$ cells. **f** GRO-seq and H3K27ac ChIP-seq signals within SEs in MyoD$^{-/-}$ and WT cells at MT stage. **g** GRO-seq tag counts of seRNAs were quantified in WT or MyoD$^{-/-}$ cells. **h** qRT-PCR detection of seRNAs from 48-hr-differentiated C2C12 cells transfected with either control or MyoG siRNA. **i** Genomic snapshots of MyoD ChIP-seq signals surrounding seRNA-1 (top) and seRNA-2 (bottom) loci show the increased MyoD binding in MT vs MB. MyoD ChIP-seq data were obtained from ENCODE. The promoter of seRNA-1 (left) or -2 (right) was fused to a luciferase reporter in a pGL3-basic vector and transfected into C2C12 cells. The activities of the reporters were increased in MT vs MB. Data in **h** represent the average of three independent experiments ± s.d and data in **i** represent the average of two independent experiments ± s.d. Statistical analysis was performed by Mann–Whitney non-parametric test (**d**) or two-tailed unpaired Student's t-test (**h**, **i**),, *P < 0.05, **P < 0.01 and ***P < 0.001. Source data are provided as a Source Data file.

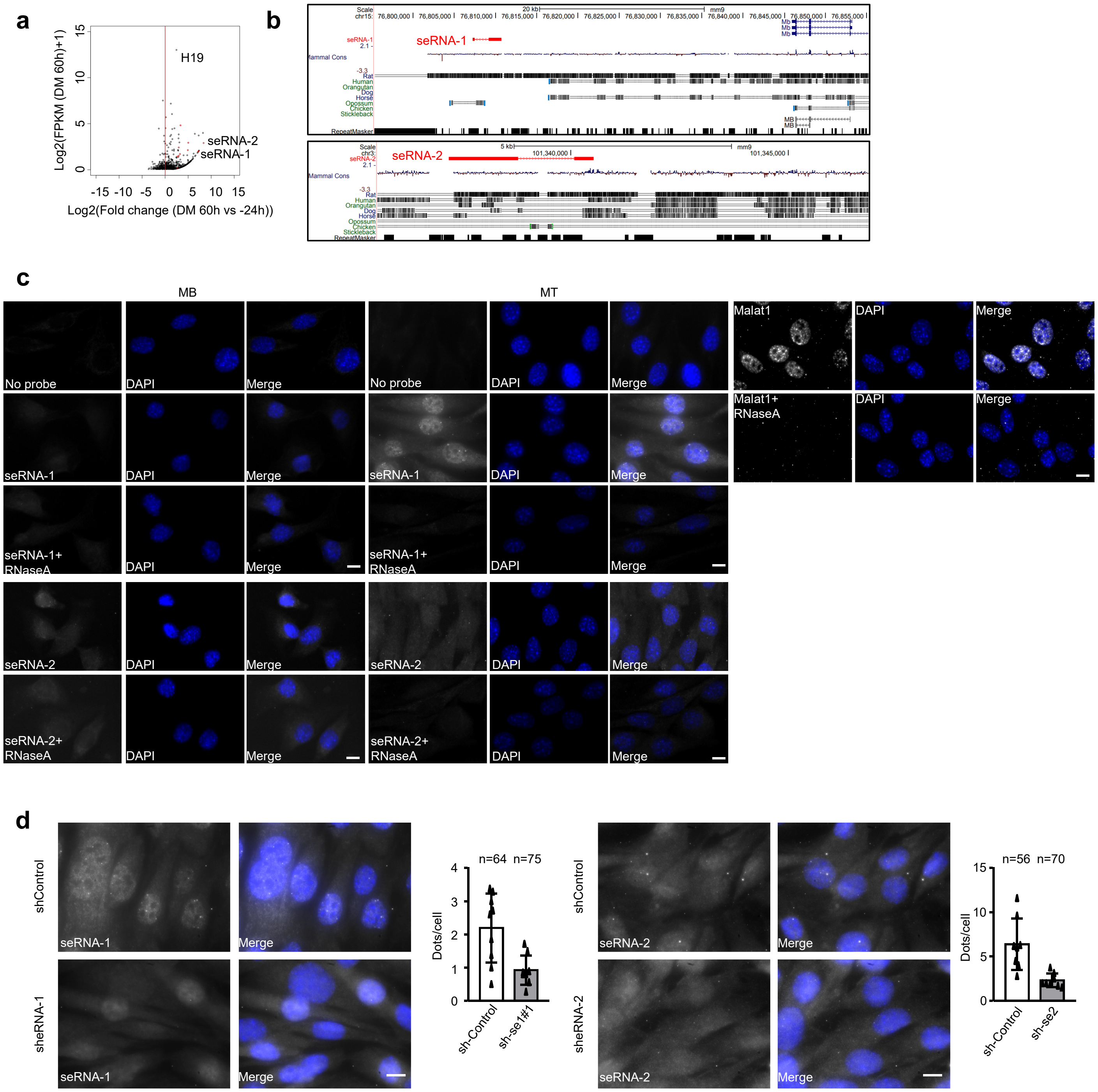

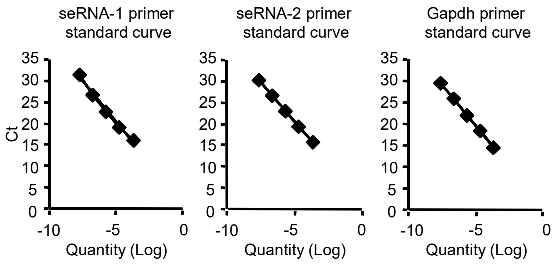

f

	Copies/Cell	
	GM	DM D2
seRNA-1	0.12	7.97
seRNA-2	2.44	10.04
Gapdh	30619.82	6404.72

g

Transcript ID	Prediction	Noncoding Score
seRNA-1	noncoding	0.9978
seRNA-2	noncoding	0.7841
seRNA-3	noncoding	0.9288
seRNA-4	noncoding	0.9496
seRNA-5	noncoding	0.9793
seRNA-6	noncoding	0.9659
seRNA-7	noncoding	0.9933
seRNA-8	noncoding	0.9868
seRNA-9	noncoding	0.9794
seRNA-10	noncoding	0.9867
seRNA-11	noncoding	0.9864

Supplementary Figure 5. seRNA-1 and seRNA-2 copy number and cellular localization. a Selection of MT SE-associated seRNAs. The x axis shows seRNAs expression in MT (DM 60h) versus MB (-24h) (log2 fold change) quantified from RNA-seq data; The y axis shows expression in MT (DM 60h) (log2 FPKM+1) quantified from RNA-seq data. Red dots indicate the selected seRNAs for validation. seRNA-1 and seRNA-2 are highly induced during MB differentiation. **b** Snapshots of PhastCons conservation track showing mouse seRNA-1 (top) and seRNA-2 (bottom) lack human orthologues. **c** Single-molecule RNA FISH (smFISH) detection of endogenous seRNA-1 and seRNA-2 in MB and MT (DM 48hr) cells. Representative images were shown. Nuclear retaining of Malat1 served as a positive control. Two negative controls were included: No probe (without adding probes) and RNase A (the slides were pre-treated RNase A to remove RNAs). Scale bar, 10 µm. **d** smFISH detection of seRNA-1 and seRNA-2 in sh-Control and sh-seRNA (DM 36hr) cells. Representative images are shown. Scale bar, 10 µm. **e** The titration curve of primers used for seRNA-1, seRNA-2 and Gapdh qRT-PCR experiments. **f** The absolute copy numbers of seRNA-1, seRNA-2 and Gapdh in C2C12 MB and MT cells (DM 48hr) were measured by calculation using the titration curve generated in **d** (refer to Methods for details). **g** Prediction of coding potential of seRNAs by iSeeRNA (http://www.myogenesisdb.org/iSeeRNA) indicates their non-coding nature.

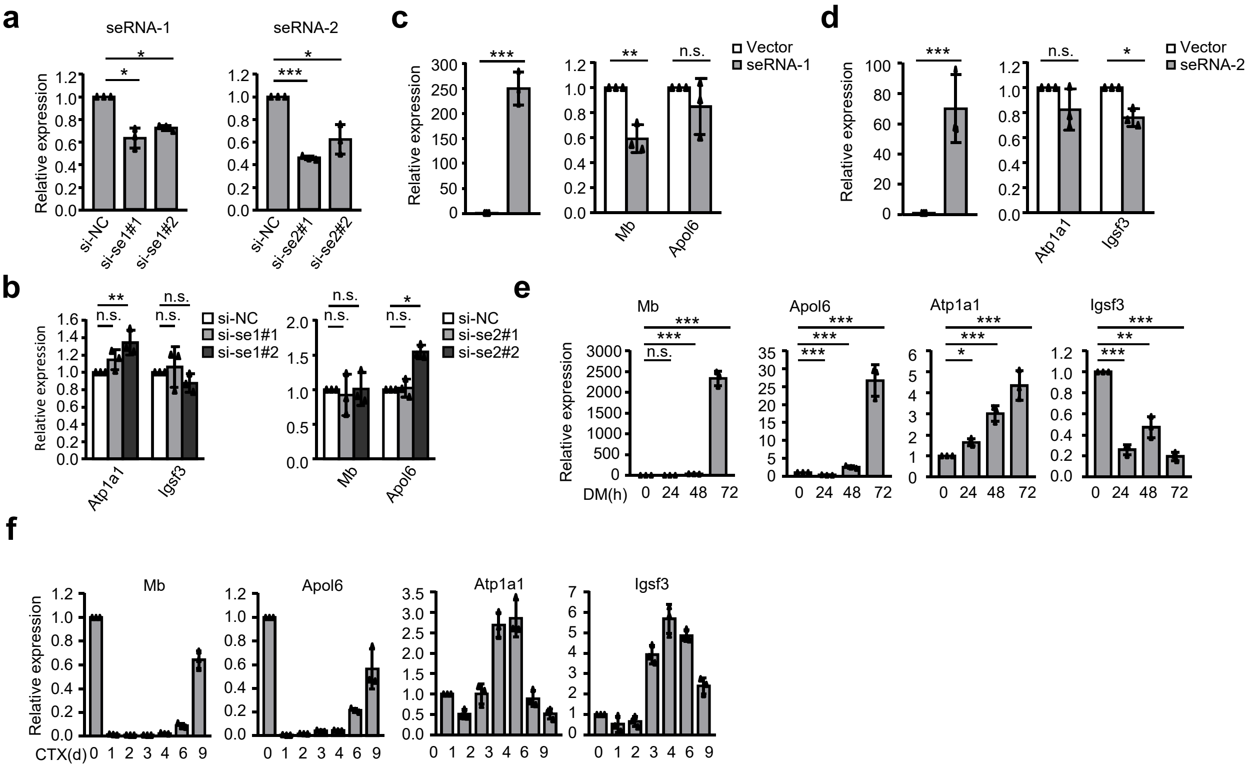

Supplementary Figure 6. seRNA-1 and seRNA-2 regulate target gene expression. a siRNA oligos against seRNA-1 (si-se1#1 or si-se1#2), seRNA-2 (si-se2#1 or si-se2#2) or scramble negative control oligos (si-NC) were transfected into C2C12 cells. At 24 h post transfection, the cells were switched to DM for 48 h. qRT-PCR analysis of target genes in RNAs purified from nuclear fractions of C2C12 cells. **b** qRT-PCR analysis of target genes in total RNAs purified from the above transfected cells. **c** Overexpression of seRNA-1 by transfecting pcDNA-seRNA-1 full-length plasmid did not strongly change the expression of its target genes, Mb and Apol6. **d** Overexpression of seRNA-2 by transfecting pcDNA-seRNA-2 full-length plasmid did not change the expression of its target genes, Atp1a1 and Igsf3. **e** Satellite cells (SCs) were FACS sorted from Tg:Pax7-nGFP mice and cultured in vitro in growth medium for 72 hr. qRT-PCR was performed to examine the expression of the target genes. **f** Two-month old C57B/L mice were injected with Cardiotoxin (CTX) in TA muscle to induce muscle injury and regeneration. qRT-PCR was performed to examine the expression of the target genes of seRNA-1 or seRNA-2 at the designated days post-injection. Data represent the average of three independent experiments ± s.d. Statistical analysis was performed by two-tailed unpaired Student's t-test (**a**, **b**, **c**, **d**, **e**), n.s., not significant, **P* < 0.05, ***P* < 0.01 and ****P* < 0.001. Source data are provided as a Source Data file.

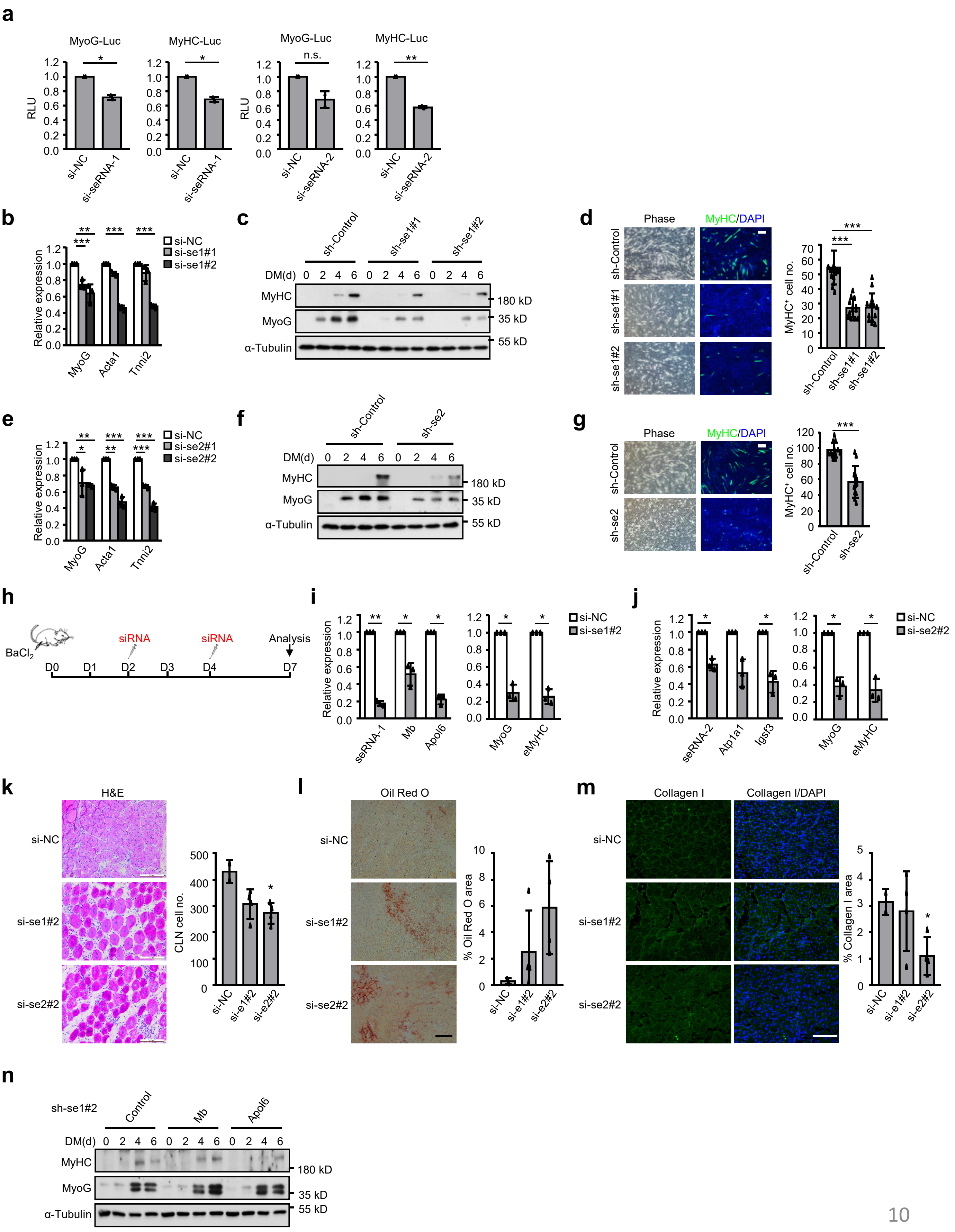
a
MyoG-Luc
MyHC-Luc
RLU
si-NC
si-seRNA-1
si-seRNA-2
n.s.
b
c
d
e
f
g
h
i
j
k
l
m
n
si-NC
si-se1#1
si-se1#2
Relative expression
MyoG
Acta1
Tnni2
sh-Control
sh-se1#1
sh-se1#2
DM(d)
MyHC
MyoG
α-Tubulin
180 kD
35 kD
55 kD
Phase
MyHC/DAPI
MyHC+ cell no.
si-se2#1
si-se2#2
sh-se2
BaCl2
siRNA
Analysis
D0
D1
D2
D3
D4
D7
seRNA-1
Mb
Apol6
eMyHC
seRNA-2
Atp1a1
Igsf3
H&E
CLN cell no.
si-e1#2
si-e2#2
Oil Red O
% Oil Red O area
Collagen I
Collagen I/DAPI
% Collagen I area
Control

Supplementary Figure. 7. seRNA-1 and seRNA-2 promote myoblast differentiation. a Knockdown of seRNA-1 or -2 by siRNA oligos decreased the activities of myogenic luciferase reporters, MyoG-Luc and MyHC-Luc, in C2C12 cells. **b** C2C12 cells were transfected with the siRNAs targeting seRNA-1 and the expression of MyoG, Acta1 and Tnni2 were measured. **c** Western blot analysis of MyHC or MyoG protein levels in control or seRNA-1 stable knockdown cells (sh-se1#1 and sh-se2#2) during a 6-day differentiation course. **d** Myotube formation was visualized in the above cells on day 6 (phase images). IF staining for MyHC was performed and the number of positively stained cells per field was quantified by counting > 15 fields per group. Scale bar, 200 µm. **e-g** The above experiments were repeated on seRNA-2. **h** Injection scheme of siRNA oligos against seRNA-1 or seRNA-2 into $BaCl_2$ injured muscles. **i** qRT-PCR detection of seRNA-1, Mb, Apol6, MyoG and eMyHC from muscles injected with either control or seRNA-1 siRNA. n = 3 per group. **j** The above experiments were performed on seRNA-2. n = 3 per group. qPCR values were normalized to 18s and Gapdh mRNA. **k** Haematoxylin and eosin (H&E) staining was performed on the above injected muscles at day 7 and centrally localized nuclei (CLN) were quantified. Scale bar, 100 µm. **l** Oil red O staining was performed on the above injected muscles at day 7. The areas positive for Oil red O staining was quantified. Scale bar, 100 µm. **m** IF staining for Collagen I was performed on the above injected muscles at day 7. The areas positive for Collagen I was quantified. Scale bar, 100 µm. **n** Mb or Apol6 was overexpressed in cells stably expressing shRNAs against seRNA-1 (sh-se1#2) and Western blot analysis of MyHC or MyoG protein levels during a 6-day differentiation course. Data represent the average of three independent experiments ± s.d. Statistical analysis was performed by two-tailed unpaired Student's t-test **(a, b, e, k, l, m)**, Mann–Whitney non-parametric test (**d, g**), or two-tailed paired Student's t-test (**i**, **j**), n.s., not significant, *$P < 0.05$, **$P < 0.01$ and ***$P < 0.001$. Source data are provided as a Source Data file.

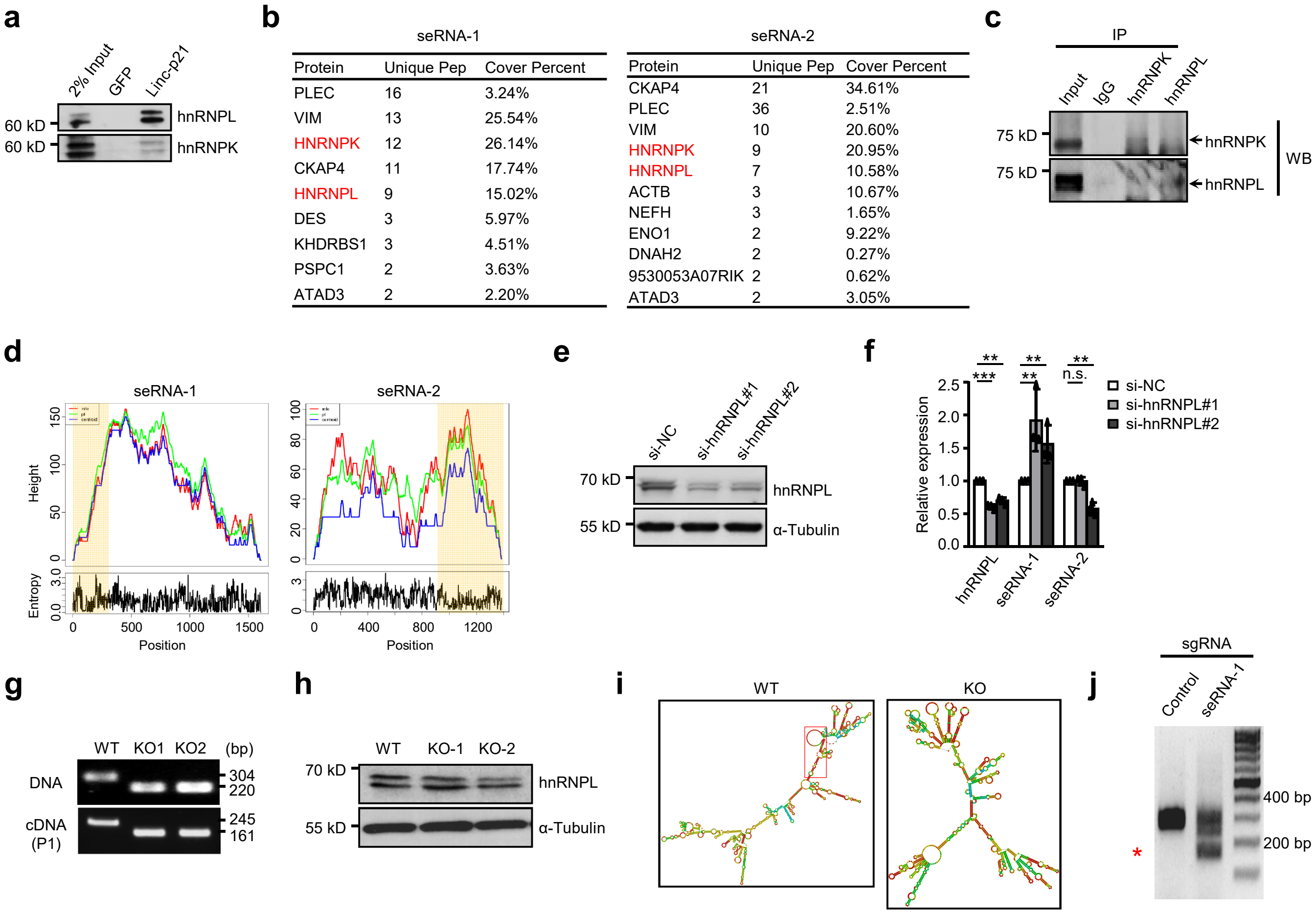

seRNA-1

Protein	Unique Pep	Cover Percent
PLEC	16	3.24%
VIM	13	25.54%
HNRNPK	12	26.14%
CKAP4	11	17.74%
HNRNPL	9	15.02%
DES	3	5.97%
KHDRBS1	3	4.51%
PSPC1	2	3.63%
ATAD3	2	2.20%

seRNA-2

Protein	Unique Pep	Cover Percent
CKAP4	21	34.61%
PLEC	36	2.51%
VIM	10	20.60%
HNRNPK	9	20.95%
HNRNPL	7	10.58%
ACTB	3	10.67%
NEFH	3	1.65%
ENO1	2	9.22%
DNAH2	2	0.27%
9530053A07RIK	2	0.62%
ATAD3	2	3.05%

Supplementary Figure 8. seRNA-1 and seRNA-2 interact with hnRNPL. a RNA pull-down assay was performed using biotinylated Linc-p21 or GFP control RNAs in cell lysates of MT cells. Western blot (WB) analysis confirms the association of Linc-p21 with hnRNPK and hnRNPL. **b** The list of the top ranked interacting proteins of seRNA-1 (left) and seRNA-2 (right) is shown with unique peptide counts and coverage percentage. **c** Immunoprecipitation of hnRNPK and hnRNPL was asserted by Western blot. Black arrows indicate hnRNPK and hnRNPL bands in the blot. **d** Mountain plot representation of the MFE structure, the thermodynamic ensemble of seRNA structures, and the centroid structure. The positional entropy for each position is presented at the bottom. **e** C2C12 cells were transfected with two different siRNAs targeting hnRNPL (si-hnRNPL) vs scrambled negative control (si-NC). At 24 h post transfection, the cells were switched to DM for 48 h. Western blot was then performed to examine the knockdown of hnRNPL. **f** Expressions of seRNA-1 and seRNA-2 were analyzed in the above si-hnRNPL vs si-NC cells by qRT-PCR. **g** CRISPR-Cas9 mediated excision of CAAA tract was performed in C2C12 cells and the deletion in two KO clones (KO1 and KO2) was confirmed in DNA by genomic PCR (top) and in mRNA by RT-PCR (bottom) using WT as control. **h** Western blot was performed to show hnRNPL protein was not significantly changed in the above KO vs WT cells. **i** The secondary structure of WT or KO transcript of seRNA-1 was predicted by RNAfold (http://rna.tbi.univie.ac.at/cgi-bin/RNAfold.cgi) with default parameters. The red box shows the location of CAAA tract in WT seRNA-1. **j** In vivo genome editing was performed in Pax7^{Cas9} mice by injecting with AAV9-sgseRNA-1. The excision of CAAA tract was detected by RT-PCR in muscle tissues injected with AAV9-sgseRNA-1, compared to AAV9-sgControl. Unedited product, 245 bp; deletion product (red asterisk), 161 bp. Data represent the average of three independent experiments ± s.d. Statistical analysis was performed by two-tailed unpaired Student's t-test (**f**), n.s., not significant, $^{**}P < 0.01$, $^{***}P < 0.001$. Source data are provided as a Source Data file.

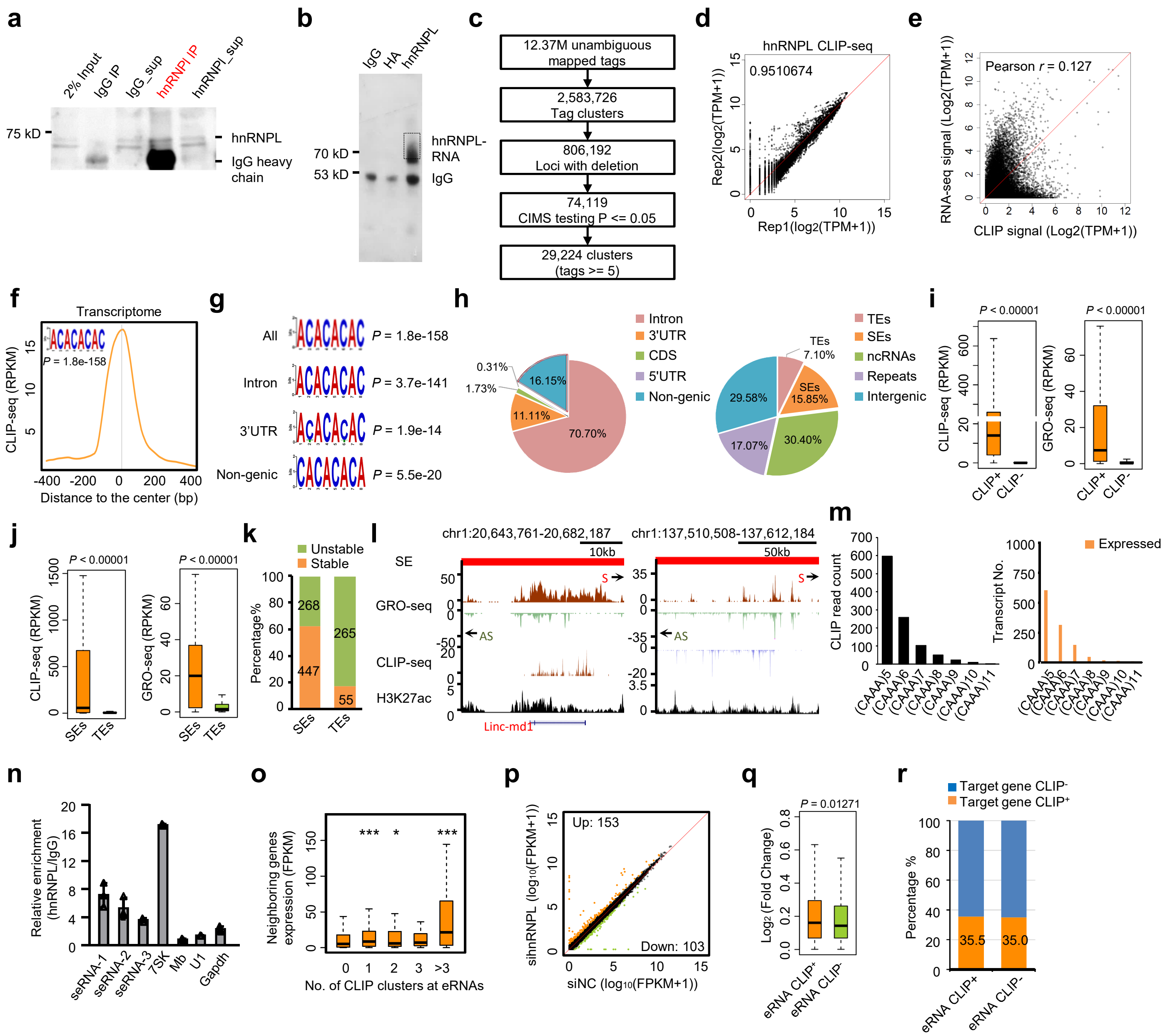
a
2% Input
IgG IP
IgG_sup
hnRNPl IP
hnRNPl_sup
75 kD
hnRNPL
IgG heavy chain
b
IgG
HA
hnRNPL
70 kD
53 kD
hnRNPL-RNA
IgG
c
12.37M unambiguous mapped tags
2,583,726 Tag clusters
806,192 Loci with deletion
74,119 CIMS testing P <= 0.05
29,224 clusters (tags >= 5)
d
hnRNPL CLIP-seq
0.9510674
Rep2(log2(TPM+1))
Rep1(log2(TPM+1))
e
Pearson r = 0.127
RNA-seq signal (Log2(TPM+1))
CLIP signal (Log2(TPM+1))
f
Transcriptome
P = 1.8e-158
CLIP-seq (RPKM)
Distance to the center (bp)
g
All
P = 1.8e-158
Intron
P = 3.7e-141
3'UTR
P = 1.9e-14
Non-genic
P = 5.5e-20
h
Intron
3'UTR
CDS
5'UTR
Non-genic
0.31%
1.73%
16.15%
11.11%
70.70%
TEs
SEs
ncRNAs
Repeats
Intergenic
TEs 7.10%
SEs 15.85%
29.58%
17.07%
30.40%
i
P < 0.00001
CLIP-seq (RPKM)
GRO-seq (RPKM)
CLIP+
CLIP-
j
P < 0.00001
CLIP-seq (RPKM)
GRO-seq (RPKM)
SEs
TEs
k
Unstable
Stable
Percentage%
268
447
265
55
SEs
TEs
l
chr1:20,643,761-20,682,187
chr1:137,510,508-137,612,184
10kb
50kb
SE
GRO-seq
CLIP-seq
H3K27ac
S
AS
Linc-md1
m
CLIP read count
Transcript No.
Expressed
(CAAA)5
(CAAA)6
(CAAA)7
(CAAA)8
(CAAA)9
(CAAA)10
(CAAA)11
n
Relative enrichment (hnRNPL/IgG)
seRNA-1
seRNA-2
seRNA-3
7SK
Mb
U1
Gapdh
o
Neighboring genes expression (FPKM)
***
*
***
No. of CLIP clusters at eRNAs
p
Up: 153
Down: 103
sihnRNPL (log10(FPKM+1))
siNC (log10(FPKM+1))
q
P = 0.01271
Log2 (Fold Change)
eRNA CLIP+
eRNA CLIP-
r
Target gene CLIP-
Target gene CLIP+
Percentage %
35.5
35.0
eRNA CLIP+
eRNA CLIP-

Supplementary Figure 9. hnRNPL binds to eRNAs transcriptome-wide in MT C2C12 cells. a Crosslinking immunoprecipitation (CLIP) was performed using an antibody against hnRNPL, HA tag, or IgG control in differentiating C2C12 cells. CLIP efficiency was determined by Western blotting. **b** 1/10 volume of the retrieved protein-RNA complexes were subjected to 3' end biotinylation and detected by streptavidin on nitrocellulose membrane. The remaining protein-RNA complexes corresponding to the size indicated by the black box were recovered for sequencing. **c** Schematic illustration of CLIP-seq analysis pipeline. **d** Comparison between two technical replicates of hnRNPL CLIP-seq data (correlation coefficient $r > 0.95$). **e** Comparison between hnRNPL CLIP-seq signals and transcript expression level (PolyA+ RNA-seq). **f** Positional distribution of the known hnRNPL binding motif CACACA within the hnRNPL CLIP clusters. **g** De novo motif search by DREME on ± 20 nt flanking sequence of the CLIP crosslinking induced mutation site (CIMS) sites. Associated *P* values are shown. **h** Genomic distribution of the above identified hnRNPL CLIP-seq clusters (left) and those uniquely mapped to the non-genic regions in (right). **i** GRO-seq read density at hnRNPL CLIP clusters in enhancer regions (CLIP+) is higher compared to non-CLIP enhancer regions (CLIP-), testifying hnRNPL binds to eRNAs transcriptome-wide. **j** SEs are associated with a higher level of hnRNPL CLIP signals compared to TEs. **k** A larger percentage of hnRNPL CLIP clusters in SEs correspond to stable eRNAs, compared to TEs. **l** Genomic snapshots of hnRNPL binding on two seRNAs. **m** Left: Long CAAA tracts identified in hnRNPL CLIP-seq raw reads fail to uniquely map to transcriptome and were eliminated from downstream analyses. Right: The number of expressed transcripts harboring long CAAA tracts. **n** hnRNPL CLIP was performed and followed by qRT-PCR analysis of retrieved RNAs. **o** Box plot shows the number of hnRNPL CLIP clusters at eRNAs is positively correlated with the expression of neighboring genes in MT cells. **p** Knockdown of hnRNPL by siRNAs led to transcriptomic changes in MT cells as determined by RNA-seq. 153 up- (red dots) and 103 down-regulated (green dots) genes were identified (≥ 1.58-FC over WT). **q** The neighboring genes with hnRNPL CLIP binding at their eRNAs showed a higher fold change (in absolute values) in si-hnRNPL vs si-NC cells compared to those with no CLIP binding at their eRNAs. **r** When comparing target genes with or without hnRNPL binding on eRNAs in **q**, the percent with hnRNPL binding on the mRNAs is comparable. *P* values in (**i**, **j**, **o**) were calculated using the Mann–Whitney non-parametric test. *P* value in (**q**) was calculated using the Fisher's Exact Test. Data in **n** represent the average of three independent experiments ± s.d. Data in **i, j, o, q** are presented in boxplot. Center line, median; box limits, upper and lower quartiles; whiskers, 1.5× interquartile range. $^{*}P < 0.05$ and $^{***}P < 0.001$. Source data are provided as a Source Data file.

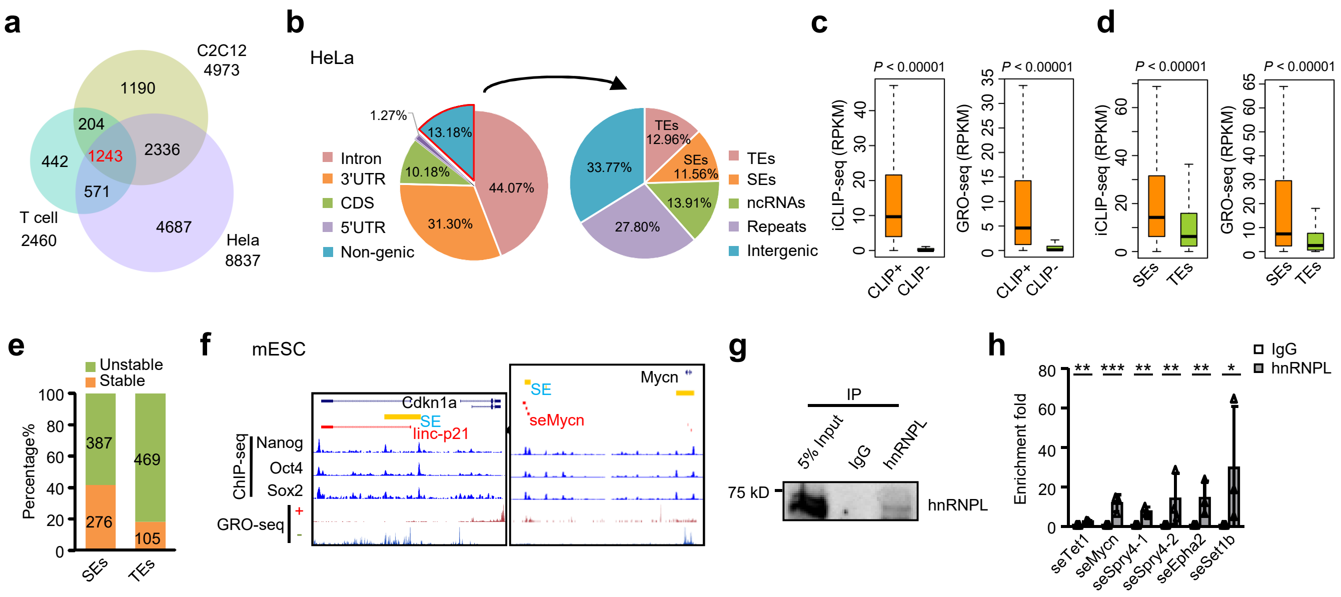

Supplementary Figure 10. hnRNPL binds to eRNAs in multiple cells. a CLIP-seq data from human HeLa cells and T cells were analyzed. The identified mRNA targets in the cells are largely overlapped with those in mouse C2C12 cells. (**b**-**e**) Analysis of hnRNPL CLIP-seq in HeLa cells revealed (**b)** the binding of hnRNPL in enhancer regions. **c** GRO-seq read density at CLIP+ enhancer regions is higher compared to CLIP- enhancer regions. **d** SEs display a higher level of hnRNPL CLIP signals compared to TEs. **e** A larger percentage of hnRNPL CLIP clusters in SEs correspond to stable eRNAs, compared to TEs. **f** Genomic snapshots of two representative seRNAs, linc-p21 and seMycn identified in mESC cells. **g** hnRNPL RIP was performed in mESC cells. Immunoprecipitation of hnRNPL was assessed by Western blot. **h** RIP was performed with antibody against hnRNPL in mESC cells and followed by qRT-PCR analysis of the indicated seRNAs. Enrichment is determined as RNAs associated to hnRNPL IP relative to IgG control. *P* values in (**c**, **d**) were calculated using the Mann–Whitney non-parametric test. Data in **h** represent the average of three independent experiments ± s.d. Data in **c**, **d** are presented in boxplot. Center line, median; box limits, upper and lower quartiles; whiskers, 1.5× interquartile range. Statistical analysis was performed by two-tailed unpaired Student's t-test (**h**). Source data are provided as a Source Data file.

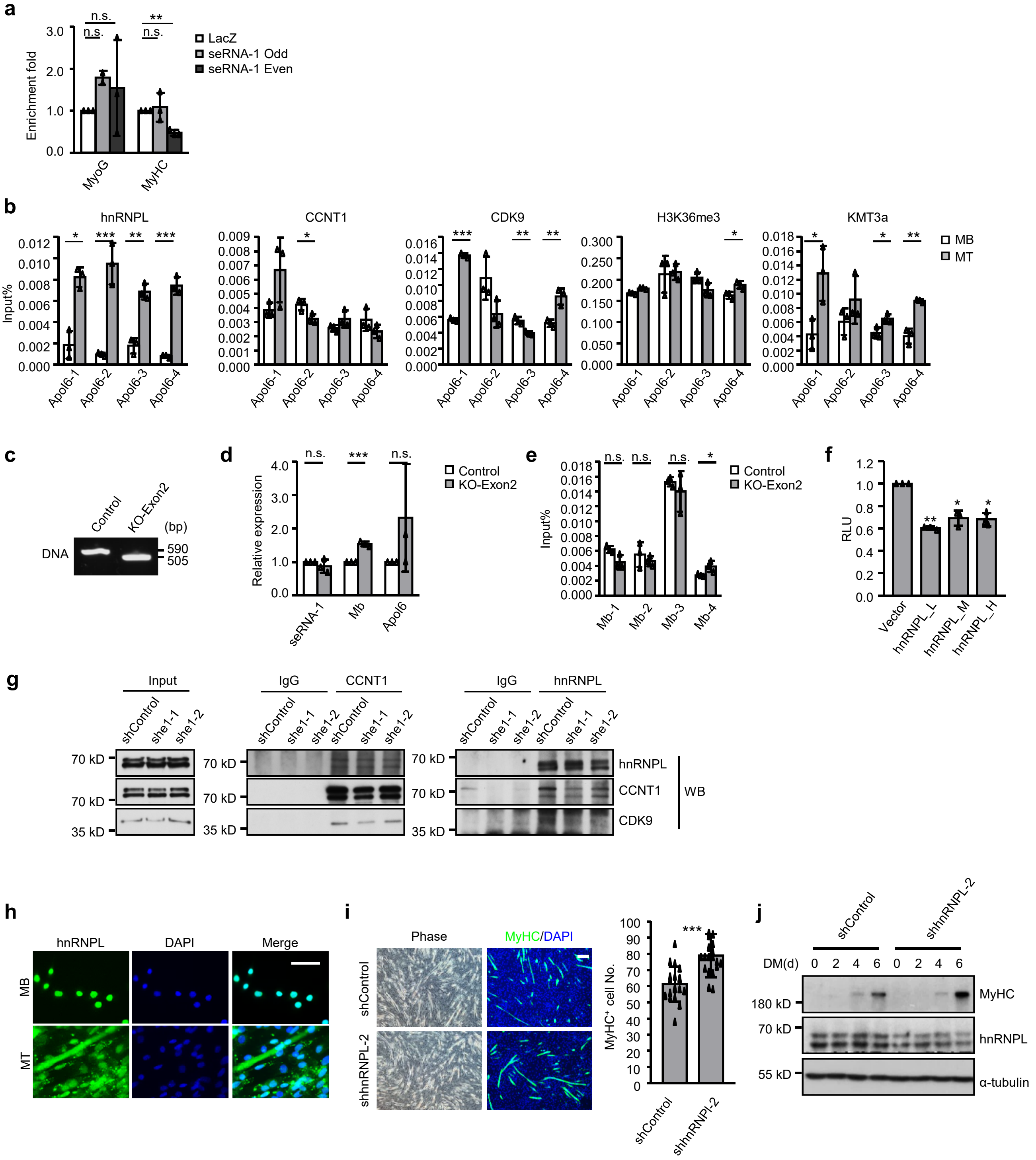

a
n.s.
n.s.
**
n.s.
LacZ
seRNA-1 Odd
seRNA-1 Even
Enrichment fold
MyoG
MyHC
b
hnRNPL
CCNT1
CDK9
H3K36me3
KMT3a
MB
MT
Input%
Apol6-1
Apol6-2
Apol6-3
Apol6-4
c
Control
KO-Exon2
(bp)
DNA
590
505
d
Control
KO-Exon2
Relative expression
seRNA-1
Mb
Apol6
e
Input%
Mb-1
Mb-2
Mb-3
Mb-4
f
RLU
Vector
hnRNPL_L
hnRNPL_M
hnRNPL_H
g
Input
IgG
CCNT1
IgG
hnRNPL
shControl
she1-1
she1-2
70 kD
35 kD
hnRNPL
CCNT1
CDK9
WB
h
hnRNPL
DAPI
Merge
MB
MT
i
Phase
MyHC/DAPI
shControl
shhnRNPL-2
MyHC⁺ cell No.
shhnRNPl-2
j
shControl
shhnRNPL-2
DM(d)
0
2
4
6
180 kD
70 kD
55 kD
MyHC
hnRNPL
α-tubulin

Supplementary Figure 11. seRNA-1 modulates hnRNPL, RNA Pol II and H3K36me3 at Mb locus. a seRNA-1 ChIRP did not retrieve DNAs corresponding to MyoG or MyHC promoter. **b** ChIP-PCR analysis of hnRNPL, CCNT1, CDK9, H3K36me3 and KMT3a at regions (1, 2, 3 and 4) across Apol6 locus in MT vs MB. **c** CRISPR-Cas9 mediated excision of a short sequence in exon 2 of seRNA-1 and the deletion in one clone (KO-Exon2) was analyzed by genomic PCR. **d** qRT-PCR detection of seRNA-1, Mb and Apol6 in the KO-Exon2 cells. **e** ChIP-PCR analysis of hnRNPL binding at Mb locus in the KO-Exon2 cells. **f** Luciferase reporter activity of Mb promoter was detected in 24-hr-differentiated C2C12 cells transfected with either control or hnRNPL expression plasmid. hnRNPL_L, low amount (30 ng); hnRNPL_M, middle amount (150 ng); hnRNPL_H, high amount (750 ng). **g** Co-IP assay for hnRNPL or CCNT1 in nuclear lysates from shControl cells or seRNA-1 knockdown cells (sh-se1#1 and sh-se2#2) at MT state. The interaction between endogenous hnRNPL and CCNT1 or CDK9 was detected. **h** IF staining for MyHC was performed in undifferentiated C2C12 myoblasts (MB) and C2C12 cells differentiated for 48 hr (MT). **i** Myotube formation was visualized in the hnRNPL knockdown cells on day 6 (phase images). IF staining for MyHC was performed and the number of positively stained cells per field was quantified by counting 19 fields per group. Scale bar, 200 µm. **j** Western blot analysis of MyHC level in the hnRNPL knockdown cells. Data represent the average of three independent experiments ± s.d. Statistical analysis was performed by Mann–Whitney non-parametric test (**i**) or two-tailed unpaired Student's t-test (**a**, **b**, **d**, **e**, **f**), n.s., not significant, *$P < 0.05$, **$P < 0.01$, ***$P < 0.001$. Source data are provided as a Source Data file.

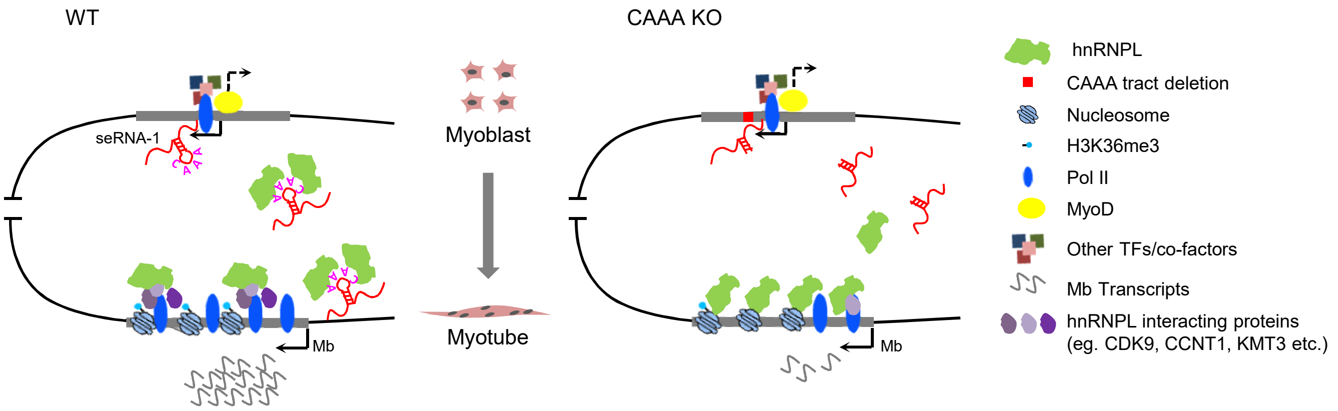

Supplementary Figure 12. A schematic model of seRNA-1 function in transcriptional activation of target gene Mb. Upon MB differentiation into MT, the master TF MyoD and co-activators activate MT enhancers and stimulate the transcription of MT eRNAs, among which seRNA-1 is highly induced. seRNA-1 plays an active role in promoting target Mb expression. Mechanistically, seRNA-1 interacts with hnRNPL via a CAAA tract to modulate hnRNPL, Pol II and H3K36me3 at Mb locus. Deletion of CAAA tract in the KO cells abolishes seRNA-1/hnRNPL interaction and leads to increase in hnRNPL but decrease in Pol II and H3K36me3 binding across Mb locus, which attenuates the expression of Mb.

Supplementary Note

Supplementary Note 1 (From Results: Elucidation of enhancer transcription in muscle cells)

Gene ontology (GO) analysis suggests the neighboring genes associated with up-regulated eRNAs are linked to muscle structure development, movement of cell or subcellular component, muscle cell differentiation and cell adhesion (Supplementary Figure 1f and Supplementary Data 1), while those associated with down-regulated are largely related to regulation of molecular function, regulation of cell proliferation, cellular response to stress and intracellular signal transduction (Supplementary Figure 1f and Supplementary Data 1).

To characterize distinct categories of eRNAs, we examined associated histone modifications, TF binding and Pol II deposition on the eRNA loci from MT. In particular, the Bi-stable and Uni-stable classes are enriched in chromatin signatures linked to enhancer activity (H3K27ac, monomethylation of histone H3 at lysine 4 (H3K4me1), acetylation of histone H3 at lysine 9 (H3K9ac) and acetylation of histone H4 at lysine 12 (H4K12ac)), active transcription initiation (trimethylation of histone H3 at lysine 4 (H3K4me3)) and active transcription elongation (dimethylation of histone H3 at lysine 79 (H3K79me2) and trimethylation of histone H3 at lysine 36 (H3K36me3)) (Supplementary Figure 2b). In contrast, unstable eRNAs bear repressive chromatin feature with a higher trimethylation of histone H3 at lysine 27 (H3K27me3) signal (Supplementary Figure 2b). In terms of TF binding, in general, stable eRNAs have a higher number of examined TFs compared with unstable ones (Supplementary Figure 2c). Consistently, increasing TF binding results in higher eRNAs expression (Supplementary Figure 2d). In addition, stable eRNAs are associated with higher local enrichment for MyoD, MyoG, TCF12 (Transcription factor 12), TCF3, PBX1 (Pre-B cell leukemia transcription factor) and FoxO3 (Forkhead box 3), all of which were previously shown to be key factors in enhancer assembly and activity (Supplementary Figure 2e). This pattern was also observed for EP300, C/EBP β and MEF2D but less significant for YY1 (Supplementary Figure 2e). When compared with other types of transcripts such as protein coding genes (PCGs) and lncRNAs, eRNAs in general were enriched for chromatin signatures linked to enhancer activity (H3K27ac, H3K4me1, H4K12ac) and associated with a higher number of TFs binding (Supplementary Figure 2b, c, e). In contrast, PCGs displayed comparable level of transcription with Bi-stable eRNAs (GRO-seq signal and Pol II binding) and markedly higher levels of chromatin marks (H3K4me3, H3K79me2, and H3k36me3) than eRNAs

and lncRNAs. In addition, lncRNAs were in general associated with comparable levels of GRO-seq signal, Pol II binding, H3K4me3, and H3K36me3 with unstable eRNAs but enriched for H3K79me2 (Supplementary Figure 2b, c, e). When analyzing eRNA loci from MB, the above described distinct features of Poll II binding, histone marks and TF binding were also found on the three subtypes of eRNAs (Supplementary Figure 2f-j).

To examine whether seRNAs show tissue/cell specificity, we calculated tissue specificity score using RNA-seq data from 18 tissues/cells [1, 2] and found the seRNAs exhibited higher tissue specificity in MB or MT compared with teRNAs, PCGs or lncRNAs (Supplementary Figure 3e). Nevertheless, none of the above selected seRNAs were skeletal muscle specifically expressed by RNA-seq analysis (Supplementary Data 2). Consistently, by RT-PCR examination of their expressions in 9 types of tissues including limb, heart, liver, spleen, lung, kidney, brain, white adipose tissue (WAT), and brown adipose tissue (BAT), only two of 17 are highly enriched in limb across a wide range of mouse tissues (Supplementary Figure 3f).

Supplementary Note 2 (From Results: seRNA-1 and seRNA-2 regulate target gene expression)

The early induction of seRNA-1 and seRNA-2 during C2C12 and SC differentiation suggested to us that they may be pro-myogenic factors during MB differentiation. To test this notion, we knocked down seRNA-1 or -2 and luciferase reporter assays using Myogenin and MyHC promoter reporters consistently revealed inhibited activities with seRNA-1 or -2 reduction (Supplementary Figure 7a). In addition, a delayed differentiation was observed with seRNA-1 or -2 knockdown as assessed by RNA expression of several myogenic markers, Myogenin, Tnni2 and α-Actin (Supplementary Figure 7b, e). Stable knockdown of seRNA-1 or seRNA-2 using short hairpin (sh) RNAs also delayed the myogenic program over a course of 6 days as assessed by protein levels of myogenic marker, MyHC (Supplementary Figure 7c, f) or immunofluroscence (IF) staining of MyHC-positive cells (Supplementary Figure 7d, g). These findings confirmed that seRNA-1 and seRNA-2 are pro-myogenic factors during C2C12 differentiation. Moreover, to extend the in vitro findings to in vivo, we dissected the functions of seRNA-1 and seRNA-2 in $BaCl_2$-induced muscle regeneration. Treatment of regenerating muscles with siRNA oligos against seRNA-1 or seRNA-2, following the scheme in Supplementary Figure 7h, reduced the expression of their neighboring genes as well as

myogenic markers, Myogenin and embryonic-MyHC (e-MyHC, a marker for regenerating fibres) (Supplementary Figure 7i, j). In addition, the treatment of seRNA-1 also slightly reduced the number of newly formed fibres (Supplementary Figure 7k), and marginally increased adipose tissue deposition (Oil Red O staining, Supplementary Figure 7l), although no obvious effect on Collagen I staining (Supplementary Figure 7m). Knockdown of seRNA-2, on the other hand, reduced the number of newly formed fibers and increased adipose tissue deposition (Supplementary Figure 7k-m). The above results demonstrated seRNA-1 and seRNA-2 may be required for muscle regeneration process. Nevertheless, since the above siRNA injection strategy cannot specifically target seRNAs expressed in satellite cells, more solid genetic evidence is needed to confirm seRNA functionality in muscle regeneration in vivo.

Interestingly, over-expressing Mb or Apol6 into the above seRNA-1 knockdown cells did not rescue the delayed myogenic differentiation (Supplementary Figure 7n) probably because these proteins are associated with myotube structure and function [3] which have no impact on early differentiation. These findings suggested that additional mechanism may exist to mediate seRNA-1 regulation of differentiation program such as Myogenin expression.

Supplementary Note 3 (From Results: Mapping of transcriptome-wide binding of hnRNPL with eRNAs)

Very intriguingly, seRNA-1 was not detected among the hnRNPL CLIP targets. We reason that the repeats eliminating step during data processing may have led to the elimination of seRNA-1 with 8 CAAA repeats. Indeed, ~ 1000 transcripts expressed in MT cells harboring the long CAAA repeats and a number of raw CLIP reads containing long CAAA repeats failed to uniquely align to the transcriptome (Supplementary Figure 9m). No CLIP tags were obtained for seRNA-2 either probably because the stringent condition used in CLIP procedure disrupted the relatively weaker interaction between seRNA-2 and hnRNPL. Nevertheless, hnRNPL CLIP-PCR revealed 7.13- and 5.23- fold enrichment for seRNA-1 and seRNA-2 respectively when compared to IgG control (Supplementary Figure 9n), further consolidating the interactions between hnRNPL and seRNA-1 or -2.

To further test whether the interaction between hnRNPL and eRNAs is a general paradigm beyond

myoblast cells, we analyzed the publically available hnRNPL CLIP-seq data from HeLa [4] and T cells [5]. Interestingly, there is a significant overlapping between the above identified hnRNAPL-bound RNA targets in MT cells with those identified in HeLa (3,579, ~ 72%) and T cells (1,447, ~ 30%) (Supplementary Figure 10a), suggesting hnRNPL-RNA interactome, to some degree, is conserved among distinct cell types. Similarly, the majority of hnRNPL-bound RNAs in HeLa cells are localized in genic region with a significant portion arising from enhancer regions (Supplementary Figure 10b). Again, hnRNPL CLIP positive sites in enhancers displayed much higher GRO-seq reads than those with no CLIP binding (Supplementary Figure 10c). Also, a higher enrichment of hnRNPL CLIP signals was detected in SEs compared to TEs (Supplementary Figure 10d) and the seRNA tags were more stable (Supplementary Figure 10e), all in agreement with the observations in the above MT cells. Lastly, in mESC cells, we were able to experimentally confirm hnRNPL binding to several seRNAs (Supplementary Figure 10f-h). Taken together, these data demonstrate that hnRNPL interaction with eRNAs may be a general phenomenon in cells.

Supplementary Note 4 (From subsection: Discussion)

Collectively our data led us to speculate that in MT the hnRNPL amount on the target promoter is critical to induce gene expression and seRNA-1 binding with hnRNPL functions to prevent the overloading of hnRNPL. Therefore, loss of seRNA-1 binding by knockdown or removing CAAA tract led to aberrant enrichment of hnRNPL on the target locus and inhibited the gene activation (Figs. 3f, 5d, 6f, 6g), which can be phenocopied by overexpressing hnRNPL in cells (Fig. 6g). Tethering a seRNA-1 also caused increased hnRNPL binding on the target locus thus similar transcriptional inhibition (Fig. 6i-k). The tight control is probably executed stochastically through the molecular ratio between seRNA-1 and hnRNPL binding. As determined in Supplementary Figure 5f, a relatively low number of seRNA-1 molecules were found in MT cells which can bind with a certain number of hnRNPL proteins on the target locus. More solid evidence on this can be obtained to support this in the future. Furthermore, we believe the transcriptional activating role of hnRNPL is mediated by its interaction with Pol II elongation machinery according to prior reports [6, 7, 8]. Indeed we showed that hnRNPL interacts with CDK9 and CCNT1 (Fig. 6d) and concordant enrichment of all three proteins were seen on the target promoter in myotube (Fig. 6c); interestingly, seRNA-1 knockdown led to increased binding of hnRNPL and CDK9 but decreased CCNT1 binding,

suggesting that the complex interaction of hnRNPL with transcriptional machinery needs to be further investigated. For example, it will be interesting to dissect whether hnRNPL, like other hnRNP proteins and RNA processing factors, may partake in releasing of core P-TEFb from the 7SK snRNP [6, 9, 10] and seRNA-1 transcript at Mb locus could interact with hnRNPL and modulate the release of hnRNPL and P-TEFb complex from 7SK sequestration [6, 9, 10, 11]. Lastly, it remains unclear how hnRNPL is recruited to the Mb target and how the selectivity is achieved. We believe this may not involve seRNA-1 as loss of seRNA-1 actually led to increased recruitment.

Some lines of evidence suggest that our model may very likely extend beyond seRNA-1. For example, we did observe higher CLIP-seq tags in eRNAs are associated with higher expression level of eRNAs neighboring genes (Supplementary Figure 9o) and neighboring genes with eRNAs binding are more vulnerable to hnRNPL knockdown (Supplementary Figure 9q). Nevertheless, it is intriguing that seRNA-2 binding with hnRNPL does not appear to imply regulatory function on its target genes. This could be due to a relative weaker interaction seRNA-2 with hnRNPL; indeed seRNA-2 has only one CACACA tract while seRNA-1 has eight repeats of CAAA tracts. Therefore, our proposed model needs to be validated on more examples of seRNAs in the future. Altogether our findings further underscore the function of hnRNPL in transcriptional regulation, expanding its traditional framework in RNA alternative splicing.

Lastly, our findings demonstrated a clear role for seRNA-1 and -2 in regulating myogenic differentiation. Knockdown of seRNA-1 or -2 decreased the levels of both MyoG and MyHC; the results from using MyoG or MyHC reporter (Supplementary Figure 7a) suggested that seRNA-1 and -2 appeared to work in trans to affect the activity of their promoters. Still, ChIRP-PCR showed no binding of seRNA-1 on MyoG and MyHC promoter was found (Supplementary Figure 11a). Therefore, it is currently unclear how seRNA-1 regulates MyoG or MyHC. Despite the solid evidence showing the activating role of seRNA-1 and -2 on their target loci, over-expressing Mb or Apol6 into C2C12 cells did not rescue the delayed myogenesis caused by seRNA-1 knockdown (Supplementary Figure 7n). We reason that these proteins are structural and functional proteins in myotubes thus may not have impact on myogenic differentiation. These data seems to suggest that additional mechanism may exist to mediate seRNA-1 regulation of myogenesis, which may not even

involve hnRNPL. Furthermore, our results revealed that hnRNPL represses myoblast differentiation, which is opposite to seRNA-1's pro-myogenic function (Supplementary Figure 11i, j). We reason that this may be caused by the pleiotropic roles of hnRNPL plays in gene regulation. In addition to regulating seRNA targets at a transcriptional level, it is widely known as a splicing regulator impacting many mRNAs. We thus believe the functional synergism between seRNA and hnRNPL should be dissected on their target genes using transcription as a read-out; this view is also consistent with prior reports on mechanistic studies of eRNA [12].

Supplementary Methods

AAV9 DNA vector and virus production. To generate the dual AAV9-sgRNA plasmids, the AAV:ITR-U6-sgRNA(backbone)-pCBh-Cre-WPRE-hGHpA-ITR (Addgene, 60229) was used as donor plasmid. Coding sequencing for DsRed was PCR-amplified and cloned into the donor plasmid through replacing the sequence encoding Cre using AgeI and EcoRI sites. To enhance DsRed expression, the pCBh promoter was substituted with CMV promoter. The first sgRNA of seRNA-1 was cloned into Sap1 site of the AAV9-sgRNA vector. For the second sgRNA, the U6-sgRNA(backbone) was PCR amplified from the plasmid with the inserted sgRNA and inserted into XbaI and KpnI sites of the AAV9-sgRNA vector. Sequences of all the gRNAs and genotyping PCR primers are provided in Table S6. AAV9 was produced following a previous protocol [13]. In brief, HEK293FT cells were transiently transfected with constructed AAV9-sgRNA vectors, AAV9 serotype plasmid, and pDF6 (adenovirus helper plasmid) at a ratio of 1:1:2 (5 μg of AAV9-sgRNA vectors, 5 μg of AAV9 serotype plasmid and 10 μg of pDF6) using polyethyleneimine (PEI) when the cells reached 80% ~ 90% confluent. At 72 hr post transfection, cells were harvested and then resuspended with 1.44 ml 100 mM sodium citrate (pH 8.05) and 2.25 ml sterile H_2O. The AAV viruses were released through three sequential freeze–thaw cycles (liquid nitrogen/37°C) with vortex between each freeze-thaw cycle. The supernatant was added with $MgCl_2$ and treated with Benzonase (Sigma) at 37°C for 0.5 ~ 1 hr followed by addition of 3.06 ml 100 mM citric acid and 2.25 ml sterile H_2O. Heavy flocculate was precipitated by centrifugation at 1,530 g for 10 minutes at room temperature and the supernatant was transferred to another 15 ml conical tube. Half volume of 1 M NaCl and 10% PEG8000 (w/v) were added to the supernatant and incubated at 4°C for overnight. After spinning the mixture at 120,000 g at 4°C for 30 minutes, the supernatant was discarded and the

pellet was resuspended with sterile PBS and then subjected to centrifugation at 3,000 g for 10 minutes. The supernatant was filtered by 0.22 μm sterile filter and passed through a 100 kDa MWCO (Millipore). The concentrated solution was washed with sterile PBS for three times. The tilter of the AAV9 virus was determined by quantitative PCR using primers targeted to the CMV promoter.

seRNA copy number quantification. To quantify each RNA transcript, we purified the PCR products using the same qPCR primers for this transcript and standard curves were then generated by qPCR using these PCR products with known concentration. For example: for GAPDH, the number of ssDNA molecules from 1 μl of 20 ng μl-1 PCR product of GAPDH fragment (228bp) with 70557.52 Da molecular weight is about 2 × (20 × 10−9 × 6.023 × 1023)/70,557.52 = 3.41 × 1011. The number of ssDNA molecules from 1 μl of 20 ng μl-1 PCR product of seRNA-1 fragment (148bp) with 45199.16 Da molecular weight is about 2 × (20 × 10−9 × 6.023 × 1023)/45,199.16 = 5.33 × 1011. The number of ssDNA molecules from 1 μl of 20 ng μl-1 PCR product of seRNA-2 fragment (233bp) with 71,937.55 Da molecular weight is about 2 × (20 × 10−9 × 6.023 × 1023)/71,937.55 = 3.35 × 1011.For cDNA samples, 1 μg of total RNA (~cells in MB stage, and ~cells in MT stage) were reverse transcribed into 20 μl cDNA. We then used the established standard curve for each RNA transcript to quantify seRNAs copies from cDNA samples. Served as control, the copy number of Gapdh mRNA is consistent with previous report.

RNA Fluorescence In Situ Hybridization (FISH). Tiling oligonucleotides targeting seRNAs were designed using online software Stellaris Probe Designer (LGC Biosearch Technologies, Petaluma, CA, USA). We ordered all Stellaris™-type oligonucleotides from BGI with a Biotin-TEG modification at the 3’ end. Cells were washed with PBS and then fixed in 3.7% formaldehyde in 1 × PBS for 10 min at room temperature. After fixation, cells were washed with 1 × PBS twice and then permeabilized in 70% ethanol overnight at 4°C. Prior to the hybridization, the cells were rehydrated with the wash buffer (10% formamide, 2 × sodium citrate buffer (SSC)) for 5 mines. Then, the cells were hybridized in hybridization buffer (2 × SSC, 10% formamide, 2 mM VRC) with probe at a final concentration of 40 μM, and incubated for 20 min in a humid chamber at 37°C. After hybridization, the cells were washed three times in wash buffer at 37°C for 5 minutes. The cells were then incubated with for Cy3-Streptavidin (diluted 1:1250 in 2 × SSC, 8% formamide, Jackson, 016160084) for 30 min at RT in the dark. After two times wash with 2 × SSC, 8% formamide for 15 min, Prolong Gold antifade reagent containing DAPI (P36935, Thermo Fisher Scientific) was

applied to mount the slides. Images were taken with a 100× NA 1.4 oil objective on a Leica inverted fluorescence microscope equipped with a cooled CCD camera. 26 optical slices were acquired at 0.25 μm intervals and images were processed to 2D data using maximum projection model.

Image processing. We modified the procedures described to analysis the RNA FISH images [14, 15]. Briefly, images for a specific RNA were parallelly converted to grayscale and conducted maximum projection operation. Later, the parameters of Laplacian of Gaussian (LoG) filter, i.e. hsize and sigma, were determined pursuing an equilibrium between genuine signal enhancement and noise removal. We used LoG with hsize equal to 10 and sigma equal to 3 for all images. Image opening operation using a disk structuring element of size 3 further helped to eliminate small noise and break narrow isthmuses. The normalized intensity thresholds for the distribution between image intensity and the number of spots identified were selected near a relatively plateau, also referring to the PCR quantification values. Considering signals in different layers can be projected into one enlarged but non-circular spot, we first identified circular spots with eccentricity less than 0.7 and area size larger than 1 on the filtered image, forming Circular Spot (CS) set with NCS spots. Area of every large (non-circular) spot was further divided by the mean area size of CS set and the rounded down value was added to NCS. After iterated all the non-circular spots, the final NCS values were recorded. For Malat1 control RNA, only spots falling into nucleus were quantified, while seRNA spots falling inside each nucleus or outside nucleus were quantified separately.

Nuclear run-on. About 5 × 10^6 C2C12 cells were harvested and resuspended in cold swelling buffer (10 mM Tris-HCl pH7.5, 2 mM $MgCl_2$, 3 mM $CaCl_2$) on ice for 5 minutes and then lysed in lysis buffer (swelling buffer + 0.5% IGEPAL + 10% glycerol + 2 U ml^{-1} SUPERase In, and cOmplete protease inhibitors) followed by gently pipetting up and down for 20 times. After centrifuge, nuclei were sequentially washed with lysis buffer and freezing buffer (50 mM Tris-HCl pH 8.3, 40% glycerol, 5 mM $MgCl_2$, 0.1 mM EDTA) and finally resuspended in 100 μl of freezing buffer and stored at -80 °C. Nuclear run-on (NRO) assays were performed with biotin-11-UTP as previously reported. In brief, 2 × NRO master mix (10 mM Tris-HCl pH 8.0, 5 mM $MgCl_2$, 1 mM DTT, 300 mM KCl, 1% Sarkosyl, 250 μM ATP, GTP, CTP, 50 μM biotin-11-UTP, and 0.8 U μl^{-1} SUPERase In) was pre-equilibrated at 37 °C for 10 minutes. Then, 5 × 10^6 cells / 100 μl nuclei were added to the same volume 100 μl of 2 × NRO master mix and incubated at 37 °C for 30 minutes. The run-on RNA (NRO-RNA) was extracted with 2 ml of TRIzol reagent (Invitogen) following the manufacturer's

instructions and incubated with 30 µl of Dynabeads™ M-280 Streptavidin beads (Thermo Fisher Scientific) in binding buffer (10 mM Tris-HCl pH 7.4, 300 mM NaCl and 0.1% Triton X-100) for 20 minutes at room temperature while rotating. Then, the beads were sequentially washed two times with high-salt wash buffer (50 mM Tris-HCl pH 7.4, 2 M NaCl and 0.5% Triton X-100), two times with binding buffer, and one timer with low-salt wash buffer (5 mM Tris-HCl pH 7.4, 0.1% Triton X-100). Then the run-on RNA (NRO-RNA) was extracted with 300 µl of TRIzol reagent (Invitogen) and finally resuspended in 20 µl RNase-free water. RT-qPCR primers used to detect premature RNA are provided in Supplementary Data 6.

HnRNPL CLIP-seq. Differentiating C2C12 cells (2×10^7) were washed with PBS and crosslinked by irradiation one time at 254 nm for 400 mJ/cm^2 in Stratalinker UV crosslinker (Stratagene). The cells were then collected and flash-frozen in liquid nitrogen and stored at - 80°C. Cells were lysed in 1ml Lysis Buffer (100 mM KCl, 5 mM $MgCl_2$, 10 mM HEPES-KOH pH 7.0, 0.5% NP-40) supplemented with 1 mM DTT, 40 U ml^{-1} SUPERase In and cOmplete protease inhibitors for 15 min on ice with pipetting up and down for several times at intervals. Then, 2 µl of Turbo DNase (AM2238, Thermo Fisher Scientific) was added and the reaction was incubated at 37°C for 5 min with rotating at 1000 rpm. Subsequently, 10 µl of diluted RNase A (5 µg ml^{-1}, 12091021, Thermo Fisher Scientific) was applied and the reaction was further incubated at 37°C for 5 min with rotating at 1000 rpm. Cell lysate was cleared by centrifugation at 15,000 g for 20 min. Save 6 µl of cleared samples as Input sample to later determine immunoprecipitation efficiency and divide the remaining samples into three aliquots. To prepare IP buffer, 2550 µl of ice-cold lysis buffer was supplemented with 600 units SUPERase In, 30 µl of 100 mM DTT and EDTA to 20 mM and 300 µl of cleared cell lysate were then added to the IP buffer. Immunopreciptations were then performed using 10 µg anti-hnRNPL antibody (sc-28726, Santa Cruz), normal IgG control (sc-2027, Santa Cruz) and anti-HA tag (sc-805, Santa Cruz) respectively for overnight at 4°C with rotation. On the next day, 125 µl washed Dynabeads Protein G was added to each reaction and incubated for another 4 hours with rotation. After incubation, 1/10 volume of beads were saved to determine immunoprecipitation efficiency. Beads were collected and washed twice with high salt wash buffer (300 mM KCl, 5 mM $MgCl_2$, 10 mM HEPES-KOH pH 7.0, 0.5% NP-40) then PNK buffer (50 mM Tris-HCl pH 7.4, 10 mM $MgCl_2$, 0.5% NP-40). Cross-linked RNA complexes were dephosphorylated by incubating with Alkaline Phosphatase (NEB) for 20 min at 37°C, followed by treatment with T4 PNK enzyme in acid

PNK buffer (70 mM Tris-HCl pH 6.5, 10 mM $MgCl_2$) for 20 min at 37°C. Beads were washed with high salt wash buffer and PNK buffer. Protein-RNA complexes were then divided into two portions. 1/10 volume was subjected to RNA 3' end biotinylation (20160, Thermo Fisher Scientific) following the provider's procedure. Remaining 9/10 volume was used for on-bead 3' RNA linker ligation and incubated in a 30 μl reaction volume containing 50 pmol of 3' RNA adaptor (5'p-rGrArUrCrGrUrCrGrGrArCrUrGrUrArGrArArCrUrCrUrGrArArC-/3'InvdT/) (IDT), 30 nmol of ATP, 30 unites of T4 RNA ligase I (NEB, Ipswich, MA), 20 unites RNase inhibitor and 10% PEG 8000 at 20℃ for overnight, shaking at 1200 rpm. After extensive wash, beads (3' RNA adaptor ligated) were 5' ends phosphorylated with T4 polynucleotide kinase (PNK) followed by wash steps. The beads (biotin labeled or 3' RNA adaptor ligated) were then denatured in loading buffer and resolved on a Novex NuPAGE 10% Bis-Tris gel and then transferred to 0.45 μm nitrocellulose membranes for 2h at 60V. The biotin reaction group was then subjected to biotin detection using chemiluminescent nucleic acid detection Module (89880, Thermo Fihser Scientific) to indicate labeled RNA complexes. For western blot analysis to evaluate the IP efficiency, CLIP membranes were probed with anti-hnRNPL antibody for 16 hr overnight. For sequencing, labeled hnRNPL-RNA bands were excised from the membrane (50 - 20 kDa above the MW of hnRNPL) and eluted by incubating with PK buffer (100 mM Tris-HCl pH 7.4, 50 mM NaCl, 10 mM EDTA) and Proteinase K at 4 mg ml^{-1} (Thermo Fisher Scientific) for 30 min at 37°C, then for another 30 min at 37°C with PK buffer, 3.5 M Urea and Proteinase K. RNA was subsequently purified using acidic phenol:chloroform (ThermoFisher) and ethanol precipitation. Then the RNA was ligated to 5' RNA adaptor (5'-rCrCrUrUrGrGrCrArCrCrCrGrArGrArArUrUrCrCrA-3') (IDT) in a 10 μl reaction volume containing 50 pmol of 5' RNA adaptor, 10 nmol of ATP, 10 unites of T4 RNA ligase I (NEB, Ipswich, MA), 40 unites RNase inhibitor and 10% PEG 8000 at 20℃ for 6 hours. Following the ligation, ligated RNA was purified by the acidic phenol:chloroform (ThermoFisher) and ethanol precipitation. The resultant RNA was reverse transcribed with RP1 primer (5'-AATGATACGGCGACCACCGAGATCTACACGTTCAGAGTTCTACAGTCCGA-3') using Superscript III RT enzyme (Thermo Fisher Scientific). A small portion of cDNA was serial diluted and subjected to test PCR amplification to determine optical PCR cycle. The full-scale PCR amplification was performed using Q5® High-Fidelity 2× Master Mix (NEB) with 12.5 pmol of RP1 primer and RPI-index primers (5'

-CAAGCAGAAGACGGCATACGAGATNNNNNNGTGACTGGAGTTCCTTGGCACCCGAGAATTCCA-3', in which 'NNNNNN' is an index sequence). The optimal PCR cycle for full-scale amplification is 17. PCR products were PAGE separated and DNA from 140 bp to 350 bp was eluted from PAGR gel. Then, the library was quantified and sequenced on the Illumina HiSeq 1500.

Oil Red O staining. In brief, the slides were fixed in 3.7% formaldehyde for 1h at room temperature. After brief washing with deionized water, the slides were then stained in Oil Red O working solution for 30 min at room temperature. Following that, the slides were rinsed in deionized water and then in running tape water for 5 min. The slides were then mounted and taken photos under microscope.

MASS spectrometry (MS). After RNA pull-down assays were performed, the retrieved proteins were then resolved on 10% SDS-PAGE gel followed by silver staining procedure according to the protocol compatible with MASS spectrometry analysis. The specific band present in the seRNA sense transcript lane was carefully cut out and sent out for Q exactive Mass Spectrometer analysis (Thermo Finnigan, San Jose, CA) provided by Shanghai Applied Protein Technology.

CLIP-seq analysis. Low-quality reads were filtered out and adaptor sequences were trimmed from raw reads using Trimmomatic-0.36 [16]. The remaining reads were then aligned to the mouse genome (mm9) using Bowtie2 (version 2.2.4) with adapted parameters (--very-sensitive --rdg 5,2 --score-min L,-0.6,-0.7). If multiple reads were aligned to the same genomic position, only one read per position was kept for downstream analyses. Crosslinking-Induced Mutation Sites (CIMS) were identified following the published procedure [17]. Briefly, the overlapping CLIP reads were grouped to define CLIP clusters and mutations were called among those clusters; hnRNPL potential binding sites were called based on a permutation strategy, which can distinguish clustered versus randomly distributed mutations. Finally, a confident hnRNPL binding site was identified using FDR < 0.05 and supporting read count no less than 5 as the cutoff. For de novo motif analysis, DREME was used using 40 bp sequences centered on hnRNPL binding sites, and TOMTOM was then used to compare identified motif against a database of known motifs. The hnRNPL binding sites were annotated with the annotatePeaks module in HOMER (v4.4) against the mm9 mouse genome.

Supplementary References

1. Cabili MN*, et al.* Integrative annotation of human large intergenic noncoding RNAs reveals global properties and specific subclasses. *Genes & development* **25**, 1915-1927 (2011).
2. Sun K, Wang H, Sun H. mTFkb: a knowledgebase for fundamental annotation of mouse transcription factors. *Scientific reports* **7**, 3022 (2017).
3. Kanatous SB*, et al.* Hypoxia reprograms calcium signaling and regulates myoglobin expression. *American journal of physiology Cell physiology* **296**, C393-402 (2009).
4. Rossbach O*, et al.* Crosslinking-immunoprecipitation (iCLIP) analysis reveals global regulatory roles of hnRNP L. *RNA biology* **11**, 146-155 (2014).
5. Shankarling G, Cole BS, Mallory MJ, Lynch KW. Transcriptome-wide RNA interaction profiling reveals physical and functional targets of hnRNP L in human T cells. *Molecular and cellular biology* **34**, 71-83 (2014).
6. Giraud M*, et al.* An RNAi screen for Aire cofactors reveals a role for Hnrnpl in polymerase release and Aire-activated ectopic transcription. *Proceedings of the National Academy of Sciences of the United States of America* **111**, 1491-1496 (2014).
7. Huang Y*, et al.* Mediator complex regulates alternative mRNA processing via the MED23 subunit. *Molecular cell* **45**, 459-469 (2012).
8. Yuan W*, et al.* Heterogeneous nuclear ribonucleoprotein L Is a subunit of human KMT3a/Set2 complex required for H3 Lys-36 trimethylation activity in vivo. *The Journal of biological chemistry* **284**, 15701-15707 (2009).
9. Barrandon C, Bonnet F, Nguyen VT, Labas V, Bensaude O. The transcription-dependent dissociation of P-TEFb-HEXIM1-7SK RNA relies upon formation of hnRNP-7SK RNA complexes. *Molecular and cellular biology* **27**, 6996-7006 (2007).
10. Van Herreweghe E*, et al.* Dynamic remodelling of human 7SK snRNP controls the nuclear level of active P-TEFb. *The EMBO journal* **26**, 3570-3580 (2007).
11. Ji X*, et al.* SR proteins collaborate with 7SK and promoter-associated nascent RNA to release paused polymerase. *Cell* **153**, 855-868 (2013).
12. Li W*, et al.* Functional roles of enhancer RNAs for oestrogen-dependent transcriptional activation. *Nature* **498**, 516-520 (2013).
13. Keller C, Hansen MS, Coffin CM, Capecchi MR. Pax3:Fkhr interferes with embryonic Pax3 and Pax7 function: implications for alveolar rhabdomyosarcoma cell of origin. *Genes & development* **18**, 2608-2613 (2004).
14. Raj A, van den Bogaard P, Rifkin SA, van Oudenaarden A, Tyagi S. Imaging individual mRNA molecules using multiple singly labeled probes. *Nature methods* **5**, 877-879 (2008).
15. Levesque MJ, Raj A. Single-chromosome transcriptional profiling reveals chromosomal gene expression regulation. *Nature methods* **10**, 246-248 (2013).
16. Bolger AM, Lohse M, Usadel B. Trimmomatic: a flexible trimmer for Illumina sequence data. *Bioinformatics* **30**, 2114-2120 (2014).
17. Moore MJ, Zhang C, Gantman EC, Mele A, Darnell JC, Darnell RB. Mapping Argonaute and conventional RNA-binding protein interactions with RNA at single-nucleotide resolution using HITS-CLIP and CIMS analysis. *Nature protocols* **9**, 263-293 (2014).
