# Supplementary material for: MyoD induced enhancer RNA interacts with hnRNPL to activate target gene transcription during myogenic differentiation
Source: Nat Commun. 2019 Dec 19;10:5787. doi: 10.1038/s41467-019-13598-0 (PMC6923398; doi:10.1038/s41467-019-13598-0)
Supplement: Supplementary file 3 — Reporting Summary [file 41467_2019_13598_MOESM3_ESM.pdf]

nature research

Corresponding author(s): Huating Wang

Reporting Summary

Nature Research wishes to improve the reproducibility of the work that we publish. This form provides structure for consistency and transparency in reporting. For further information on Nature Research policies, see Authors & Referees and the Editorial Policy Checklist.

Statistical parameters

When statistical analyses are reported, confirm that the following items are present in the relevant location (e.g. figure legend, table legend, main text, or Methods section).

n/a	Confirmed	
☐	☒	The exact sample size (n) for each experimental group/condition, given as a discrete number and unit of measurement
☐	☒	An indication of whether measurements were taken from distinct samples or whether the same sample was measured repeatedly
☐	☒	The statistical test(s) used AND whether they are one- or two-sided<br>*Only common tests should be described solely by name; describe more complex techniques in the Methods section.*
☒	☐	A description of all covariates tested
☒	☐	A description of any assumptions or corrections, such as tests of normality and adjustment for multiple comparisons
☐	☒	A full description of the statistics including central tendency (e.g. means) or other basic estimates (e.g. regression coefficient) AND variation (e.g. standard deviation) or associated estimates of uncertainty (e.g. confidence intervals)
☐	☒	For null hypothesis testing, the test statistic (e.g. F, t, r) with confidence intervals, effect sizes, degrees of freedom and P value noted<br>*Give P values as exact values whenever suitable.*
☒	☐	For Bayesian analysis, information on the choice of priors and Markov chain Monte Carlo settings
☒	☐	For hierarchical and complex designs, identification of the appropriate level for tests and full reporting of outcomes
☒	☐	Estimates of effect sizes (e.g. Cohen's d, Pearson's r), indicating how they were calculated
☐	☒	Clearly defined error bars<br>*State explicitly what error bars represent (e.g. SD, SE, CI)*

Our web collection on statistics for biologists may be useful.

Software and code

Policy information about availability of computer code

Data collection

Microscopy pictures were acquired with Leica microscope system DM 6000B; Victor X4 plate reader (Perkin Elmer) was used for luminescence assays; qPCR reactions were conducted using the Light-Cycler system (Roche Applied Science)

Data analysis

GraphPad Prism version 8.0
Leica LAS AF software (LAS AF2.6.3)
Microsoft Excel v14.0
Image Pro Plus 6.0
R v3.2.3
heatmap.2 in R package

RNA-seq:
Tophat version 2.0.13
Cufflinks version 2.1.1

CLIP-seq:
Trimmomatic-0.36
Bowtie2 version 2.2.4

(parameters: --very-sensitive --rdg 5,2 --score-min L,-0.6,-0.7)
DREME in MEME suite version 4.10.0
TOMTOM in MEME suite version 4.10.0
HOMER version 4.4
CIMS (Moore et al. 2014 Nat Protocols 9:263-293)

ChIP-seq/GRO-seq:
Trimmomatic-0.36
Bowtie2 version 2.2.4 (default parameters)
MACS2 version 2.1.2
HOMER version 4.4
EdgeR version 3.7

For manuscripts utilizing custom algorithms or software that are central to the research but not yet described in published literature, software must be made available to editors/reviewers upon request. We strongly encourage code deposition in a community repository (e.g. GitHub). See the Nature Research guidelines for submitting code & software for further information.

Data

Policy information about availability of data

All manuscripts must include a data availability statement. This statement should provide the following information, where applicable:

- Accession codes, unique identifiers, or web links for publicly available datasets
- A list of figures that have associated raw data
- A description of any restrictions on data availability

The authors declare that data supporting the findings of this study are available within the article and Supplementary Information. GRO-seq, hnRNPL CLIP-seq and RNA-seq data reported in this paper were deposited in the Gene Expression Omnibus database under accession GSE114659.

Field-specific reporting

Please select the best fit for your research. If you are not sure, read the appropriate sections before making your selection.

☒ Life sciences ☐ Behavioural & social sciences ☐ Ecological, evolutionary & environmental sciences

For a reference copy of the document with all sections, see nature.com/authors/policies/ReportingSummary-flat.pdf

Life sciences study design

All studies must disclose on these points even when the disclosure is negative.

Sample size

No statistical test was used to determine sample size. Three biological replicates per group (detailed n is indicated in the figure legends) were collected to perform statistical testing. For GRO-seq, two biological replicates per group were used; for hnRNPL CLIP-seq, total RNA-seq, and polyA+ RNA-seq, one biological replicate were used.

Data exclusions

No data were excluded. But during sequencing data analysis, standard quality control steps were applied to remove low-quality reads, reads aligning to the same coordinate. In the CLIP-seq analysis, a confident hnRNPL binding site was identified using FDR < 0.05 and supporting read count no less than 5 as the cutoff. In the GRO-seq analysis, to define putative eRNAs in MB and MT, transcripts overlapping with protein-coding genes, antisense transcripts, divergent transcripts and the other genic regions (rRNA, snRNA, miRNA, snoRNA, etc) were filtered and the remaining transcripts were defined as putative eRNAs.

Replication

All experimental data was repeated in multiple independent experiments as indicated in the figure legends and source data.

Randomization

For all the animal experiments (Fig. 1k, 5e-g, Supplementary Fig. 7h-m), mice were randomly allocated to distinct groups.

Blinding

For immunofluorescence data collection (Fig. 3b, c, and Supplementary Fig. 4d, 5c, d, 7d, g, k-m, 11h, i), we performed the experiments in a blinded way. We randomly counted multiple fields per group and calculated the number of positively stained cells per field. Exact value can be found in source data. For other assays, we were not blinded to group allocation.

Behavioural & social sciences study design

All studies must disclose on these points even when the disclosure is negative.

Study description

Briefly describe the study type including whether data are quantitative, qualitative, or mixed-methods (e.g. qualitative cross-sectional, quantitative experimental, mixed-methods case study).

Research sample

State the research sample (e.g. Harvard university undergraduates, villagers in rural India) and provide relevant demographic information (e.g. age, sex) and indicate whether the sample is representative. Provide a rationale for the study sample chosen. For studies involving

existing datasets, please describe the dataset and source.

Sampling strategy	*Describe the sampling procedure (e.g. random, snowball, stratified, convenience). Describe the statistical methods that were used to predetermine sample size OR if no sample-size calculation was performed, describe how sample sizes were chosen and provide a rationale for why these sample sizes are sufficient. For qualitative data, please indicate whether data saturation was considered, and what criteria were used to decide that no further sampling was needed.*
Data collection	*Provide details about the data collection procedure, including the instruments or devices used to record the data (e.g. pen and paper, computer, eye tracker, video or audio equipment) whether anyone was present besides the participant(s) and the researcher, and whether the researcher was blind to experimental condition and/or the study hypothesis during data collection.*
Timing	*Indicate the start and stop dates of data collection. If there is a gap between collection periods, state the dates for each sample cohort.*
Data exclusions	*If no data were excluded from the analyses, state so OR if data were excluded, provide the exact number of exclusions and the rationale behind them, indicating whether exclusion criteria were pre-established.*
Non-participation	*State how many participants dropped out/declined participation and the reason(s) given OR provide response rate OR state that no participants dropped out/declined participation.*
Randomization	*If participants were not allocated into experimental groups, state so OR describe how participants were allocated to groups, and if allocation was not random, describe how covariates were controlled.*

Ecological, evolutionary & environmental sciences study design

All studies must disclose on these points even when the disclosure is negative.

Study description	*Briefly describe the study. For quantitative data include treatment factors and interactions, design structure (e.g. factorial, nested, hierarchical), nature and number of experimental units and replicates.*
Research sample	*Describe the research sample (e.g. a group of tagged Passer domesticus, all Stenocereus thurberi within Organ Pipe Cactus National Monument), and provide a rationale for the sample choice. When relevant, describe the organism taxa, source, sex, age range and any manipulations. State what population the sample is meant to represent when applicable. For studies involving existing datasets, describe the data and its source.*
Sampling strategy	*Note the sampling procedure. Describe the statistical methods that were used to predetermine sample size OR if no sample-size calculation was performed, describe how sample sizes were chosen and provide a rationale for why these sample sizes are sufficient.*
Data collection	*Describe the data collection procedure, including who recorded the data and how.*
Timing and spatial scale	*Indicate the start and stop dates of data collection, noting the frequency and periodicity of sampling and providing a rationale for these choices. If there is a gap between collection periods, state the dates for each sample cohort. Specify the spatial scale from which the data are taken*
Data exclusions	*If no data were excluded from the analyses, state so OR if data were excluded, describe the exclusions and the rationale behind them, indicating whether exclusion criteria were pre-established.*
Reproducibility	*Describe the measures taken to verify the reproducibility of experimental findings. For each experiment, note whether any attempts to repeat the experiment failed OR state that all attempts to repeat the experiment were successful.*
Randomization	*Describe how samples/organisms/participants were allocated into groups. If allocation was not random, describe how covariates were controlled. If this is not relevant to your study, explain why.*
Blinding	*Describe the extent of blinding used during data acquisition and analysis. If blinding was not possible, describe why OR explain why blinding was not relevant to your study.*

Did the study involve field work? ☐ Yes ☐ No

Field work, collection and transport

Field conditions	*Describe the study conditions for field work, providing relevant parameters (e.g. temperature, rainfall).*
Location	*State the location of the sampling or experiment, providing relevant parameters (e.g. latitude and longitude, elevation, water depth).*
Access and import/export	*Describe the efforts you have made to access habitats and to collect and import/export your samples in a responsible manner and in compliance with local, national and international laws, noting any permits that were obtained (give the name of the issuing authority, the date of issue, and any identifying information).*
Disturbance	*Describe any disturbance caused by the study and how it was minimized.*

Reporting for specific materials, systems and methods

Materials & experimental systems

n/a	Involved in the study
☐	☒ Unique biological materials
☐	☒ Antibodies
☐	☒ Eukaryotic cell lines
☒	☐ Palaeontology
☐	☒ Animals and other organisms
☒	☐ Human research participants

Methods

n/a	Involved in the study
☒	☐ ChIP-seq
☐	☒ Flow cytometry
☒	☐ MRI-based neuroimaging

Unique biological materials

Policy information about availability of materials

Obtaining unique materials	All materials are available on reasonable request from HW (huating.wang@cuhk.edu.hk)

Antibodies

Antibodies used	Western blotting: anti-MyoG (1:2000, sc-576, Santa Cruz), anti-MyHC (1:2000, M4276, Sigma), anti-hnRNPL (1:5000, sc-28726, Santa Cruz), anti-hnRNPK (1:5000, 4675, Cell Signaling), anti-MED1 (1:5000, A300-793A, Bethyl Laboratories), anti-RAD21 (1:5000, A300-080A, Bethyl Laboratories), anti-RBBP5 (1:5000, A300-109A, Bethyl Laboratories), anti-YY1 (1:2000, sc-1703, Santa Cruz), anti-MyoD (1:2000, sc-760, Santa Cruz), anti-α-Tubulin (1:5000, sc-23948, Santa Cruz), and anti-H3K36me3 (1:5000, ab9050, Abcam); Immunofluorescence: anti-MyHC (1:350, M4276, Sigma); ChIP-PCR: MyoD (sc-304 X, Santa Cruz), hnRNPL (sc-28726, Santa Cruz), Pol II (sc-899, Santa Cruz), H3K36me3 (ab9050, abcam), CDK9 (sc-484, Santa Cruz), CCNT1 (sc-10750, Santa Cruz) or IgG control (sc-2027, Santa Cruz). For each IP reaction, 2 ug of primary antibody was used for incubation with precleared nuclear extract; RIP-PCR: hnRNPL (sc-28726, Santa Cruz), hnRNPK (sc-25373, Santa Cruz) or IgG control (sc-2027, Santa Cruz). For each RIP reaction, 5 µg of each antibody was used; CLIP-seq: anti-hnRNPL antibody (sc-28726, Santa Cruz), normal IgG control (sc-2027, Santa Cruz) or anti-HA tag (sc-805, Santa Cruz). For each CLIP-seq IP reaction, 10 µg of each antibody was used; Co-IP: normal IgG control (sc-2027, Santa Cruz), hnRNPL (ARP40368_P050, Aviva Systems Biology) or CCNT1 (sc-10750, Santa Cruz). For each IP reaction, 2 µg of each antibody was used.
Validation	All antibodies are commercially available and the applications have been tested by the manufacturer.

Eukaryotic cell lines

Policy information about cell lines

Cell line source(s)	Mouse C2C12 myoblast cells (ATCC, CRL-1772), C3H/10T1/2 fibroblast cells (ATCC, CCL-226), 293T cells (ATCC, CRL-3216) were obtained from ATCC.
Authentication	Gene expression by RT-qPCR was used to confirm the myogenic marker genes expression in differentiating C2C12 myoblast cells (Fig. 1j). As a control, none of these genes expressed in C3H/10T1/2 fibroblast cells (Fig. 2h). No other cell line authentication was performed.
Mycoplasma contamination	No mycoplasma contamination.
Commonly misidentified lines (See ICLAC register)	No commonly misidentified cell lines were involved in this study.

Palaeontology

Specimen provenance	*Provide provenance information for specimens and describe permits that were obtained for the work (including the name of the issuing authority, the date of issue, and any identifying information).*
Specimen deposition	*Indicate where the specimens have been deposited to permit free access by other researchers.*
Dating methods	*If new dates are provided, describe how they were obtained (e.g. collection, storage, sample pretreatment and measurement), where they were obtained (i.e. lab name), the calibration program and the protocol for quality assurance OR state that no new dates are provided.*

☐ Tick this box to confirm that the raw and calibrated dates are available in the paper or in Supplementary Information.

Animals and other organisms

Policy information about studies involving animals; ARRIVE guidelines recommended for reporting animal research

Laboratory animals	For Cardiotoxin (CTX) injection, approximately seven-week-old male C57BL/6 mice were injected (Fig. 1k). For satellite cell sorting, approximately eight-week-old homozygous male Tg:Pax7-nGFP mice were used to isolate MuSCs (Fig. 3e, Supplementary Fig. 3b, c, 6e). For siRNA injection, approximately eight-week-old male C57BL/6 mice were used (Supplementary Fig. 7h-m). For AAV9 injection, homozygous Pax7Cas9 mice were injected with AAV9-sgRNA at P10 at a dose of 5 x 10e11 viral genomes (vg) per animal by intramuscular (i.m) injection (Fig. 5e-g). Animals were sacrificed and quadriceps muscles were isolated 4 weeks post AAV injection.
Wild animals	The study did not involve wild animals.
Field-collected samples	The study did not involve samples collected from the field.

Human research participants

Policy information about studies involving human research participants

Population characteristics	*Describe the covariate-relevant population characteristics of the human research participants (e.g. age, gender, genotypic information, past and current diagnosis and treatment categories). If you filled out the behavioural & social sciences study design questions and have nothing to add here, write "See above."*
Recruitment	*Describe how participants were recruited. Outline any potential self-selection bias or other biases that may be present and how these are likely to impact results.*

ChIP-seq

Data deposition

☐ Confirm that both raw and final processed data have been deposited in a public database such as GEO.

☐ Confirm that you have deposited or provided access to graph files (e.g. BED files) for the called peaks.

Data access links<br>*May remain private before publication.*	*For "Initial submission" or "Revised version" documents, provide reviewer access links. For your "Final submission" document, provide a link to the deposited data.*
Files in database submission	*Provide a list of all files available in the database submission.*
Genome browser session<br>(e.g. UCSC)	*Provide a link to an anonymized genome browser session for "Initial submission" and "Revised version" documents only, to enable peer review. Write "no longer applicable" for "Final submission" documents.*

Methodology

Replicates	*Describe the experimental replicates, specifying number, type and replicate agreement.*
Sequencing depth	*Describe the sequencing depth for each experiment, providing the total number of reads, uniquely mapped reads, length of reads and whether they were paired- or single-end.*
Antibodies	*Describe the antibodies used for the ChIP-seq experiments; as applicable, provide supplier name, catalog number, clone name, and lot number.*
Peak calling parameters	*Specify the command line program and parameters used for read mapping and peak calling, including the ChIP, control and index files used.*
Data quality	*Describe the methods used to ensure data quality in full detail, including how many peaks are at FDR 5% and above 5-fold enrichment.*
Software	*Describe the software used to collect and analyze the ChIP-seq data. For custom code that has been deposited into a community repository, provide accession details.*

Flow Cytometry

Plots

Confirm that:

- [x] The axis labels state the marker and fluorochrome used (e.g. CD4-FITC).
- [x] The axis scales are clearly visible. Include numbers along axes only for bottom left plot of group (a 'group' is an analysis of identical markers).
- [x] All plots are contour plots with outliers or pseudocolor plots.
- [x] A numerical value for number of cells or percentage (with statistics) is provided.

Methodology

Sample preparation	Hindlimb muscles from Tg:Pax7-nGFP mice were collected and digested with collagenase II (800 U/ml, Worthington) for 90 min at 37°C, and then the digested muscles were triturated and washed in washing medium (Hams F-10 media (Sigma), 10% HIHS (Gibco)) before SCs were liberated by treating with Collagenase II (800 U/ml)/Dispase (11 U/ml) for 30 min at 37°C. Mononuclear cells were filtered with a 70-μm cell strainer and GFP+ SCs were sorted out by BD FACSAria Fusion cell sorter (BD Biosciences) following the manufacturer's instructions.
Instrument	BD FACSAria Fusion
Software	BD FACSDiva 8.0
Cell population abundance	Muscle satellite cells (SCs) usually represents about 4% of the total population of mononucleated cells.
Gating strategy	FACS was performed following the gating strategy described in Liu et al. Nature Protocol, 2015. Mononucleated cells were first gated for live cells (FSC/SSC) and gated to exclude doublets. Cells were then separated based on GFP signal with two distinct populations observed in the sorting profiles. GFP+ cells were collected as SCs.

- [ ] Tick this box to confirm that a figure exemplifying the gating strategy is provided in the Supplementary Information.

Magnetic resonance imaging

Experimental design

Design type	*Indicate task or resting state; event-related or block design.*
Design specifications	*Specify the number of blocks, trials or experimental units per session and/or subject, and specify the length of each trial or block (if trials are blocked) and interval between trials.*
Behavioral performance measures	*State number and/or type of variables recorded (e.g. correct button press, response time) and what statistics were used to establish that the subjects were performing the task as expected (e.g. mean, range, and/or standard deviation across subjects).*

Acquisition

Imaging type(s)	*Specify: functional, structural, diffusion, perfusion.*
Field strength	*Specify in Tesla*
Sequence & imaging parameters	*Specify the pulse sequence type (gradient echo, spin echo, etc.), imaging type (EPI, spiral, etc.), field of view, matrix size, slice thickness, orientation and TE/TR/flip angle.*
Area of acquisition	*State whether a whole brain scan was used OR define the area of acquisition, describing how the region was determined.*

Diffusion MRI ☐ Used ☐ Not used

Preprocessing

Preprocessing software	*Provide detail on software version and revision number and on specific parameters (model/functions, brain extraction, segmentation, smoothing kernel size, etc.).*
Normalization	*If data were normalized/standardized, describe the approach(es): specify linear or non-linear and define image types used for transformation OR indicate that data were not normalized and explain rationale for lack of normalization.*
Normalization template	*Describe the template used for normalization/transformation, specifying subject space or group standardized space (e.g. original Talairach, MNI305, ICBM152) OR indicate that the data were not normalized.*
Noise and artifact removal	*Describe your procedure(s) for artifact and structured noise removal, specifying motion parameters, tissue signals and physiological signals (heart rate, respiration).*

Volume censoring | *Define your software and/or method and criteria for volume censoring, and state the extent of such censoring.*

Statistical modeling & inference

Model type and settings | *Specify type (mass univariate, multivariate, RSA, predictive, etc.) and describe essential details of the model at the first and second levels (e.g. fixed, random or mixed effects; drift or auto-correlation).*

Effect(s) tested | *Define precise effect in terms of the task or stimulus conditions instead of psychological concepts and indicate whether ANOVA or factorial designs were used.*

Specify type of analysis: ☐ Whole brain ☐ ROI-based ☐ Both

Statistic type for inference (See Eklund et al. 2016) | *Specify voxel-wise or cluster-wise and report all relevant parameters for cluster-wise methods.*

Correction | *Describe the type of correction and how it is obtained for multiple comparisons (e.g. FWE, FDR, permutation or Monte Carlo).*

Models & analysis

n/a	Involved in the study
☐	☐ Functional and/or effective connectivity
☐	☐ Graph analysis
☐	☐ Multivariate modeling or predictive analysis

Functional and/or effective connectivity | *Report the measures of dependence used and the model details (e.g. Pearson correlation, partial correlation, mutual information).*

Graph analysis | *Report the dependent variable and connectivity measure, specifying weighted graph or binarized graph, subject- or group-level, and the global and/or node summaries used (e.g. clustering coefficient, efficiency, etc.).*

Multivariate modeling and predictive analysis | *Specify independent variables, features extraction and dimension reduction, model, training and evaluation metrics.*
