# Supplementary material for: MyoD induced enhancer RNA interacts with hnRNPL to activate target gene transcription during myogenic differentiation
Source: Nat Commun. 2019 Dec 19;10:5787. doi: 10.1038/s41467-019-13598-0 (PMC6923398; doi:10.1038/s41467-019-13598-0)
Supplement: Supplementary file 4 — Description of Additional Supplementary Files [file 41467_2019_13598_MOESM4_ESM.docx]

**Description of Additional Supplementary Files**

File name: Supplementary Data 1

Description: GRO-seq analysis in C2C12 MB vs MT cells.

File name: Supplementary Data 2

Description: seRNAs expression in mouse tissues.

File name: Supplementary Data 3

Description: GRO-seq analysis in WT vs MyoD^-/-^ cells.

File name: Supplementary Data 4

Description: CLIP-seq analysis of hnRNPL RNA binding in MT cells.

File name: Supplementary Data 5

Description: RNA-seq analysis in si-NC vs si-hnRNPL cells.

File name: Supplementary Data 6

Description: Information of oligonucleotides and primers used in the study.

File name: Supplementary Data 7

Description: Information of publicly available datasets used in the study.
