# Supplementary material for: MyoD induced enhancer RNA interacts with hnRNPL to activate target gene transcription during myogenic differentiation
Source: Nat Commun. 2019 Dec 19;10:5787. doi: 10.1038/s41467-019-13598-0 (PMC6923398; doi:10.1038/s41467-019-13598-0)
Supplement: Supplementary file 2 — Peer Review File [file 41467_2019_13598_MOESM2_ESM.pdf]

Reviewers' comments:

Reviewer #1 (Remarks to the Author):

This manuscript is an exceptionally dense analysis of the potential role of hnRNP L in the function of super-enhancers. There are certain to be some interesting findings here, but the deluge of often-subtle data detracts from the conclusiveness and clarity of the work and the ultimate model in the final figure is difficult to rationalize.

The manuscript really needs to be reworked with focus on presenting just the critical data to support the main point (quality over quantity).

The following specific concerns minimally need to be addressed before the manuscript can be fully comprehended and judged.
1) The term "seRNA" is used in both the abstract and introduction with no definition.

2) Even if they are statistically significant, the differences in figures such as 1b and 5r are too subtle to be biologically meaningful. This raises concerns regarding the model and physiologic relevance of what is being investigated

3) Why were seRNA-1 and seRNA-2 selected for further study based on Fig 1 and 2? No justification is given for why these, among all the others, are of most interest.

4) Do the siRNAs deplete the nuclear pool of seRNA-1 and -2? This is critical for the model, but no evidence is given. Since most of the seRNAs are in the cytoplasm, looking at total isn't valid.

5) What is the specificity of the impact of the knock-downs? That is, does siRNA against seRNA-1 impact expression of Atp1a1 and Igsf3?

6) In the binding experiment in Figure 4, if known eRNA binding partners were not found, how is the validity of the experiment confirmed?

7) The fact that depletion of hnRNP L doesn't impact seRNA-2 function clearly demonstrates that binding of hnRNP L (which is more specific for seRNA-2 than -1), does not imply function. This completely undermines the value of the CLIP experiment in Figure 5. Additionally, no explanation is given for why seRNA-2 might not involve hnRNP L.

8) The logic circuit doesn't fit. Loss of seRNA-1 reduces target expression and increases binding of hnRNP L to seRNA-1 target genes, Loss of hnRNP L increases seRNA-1 expression and decreases seRNA-1 target genes. So target gene expression is reduced upon both the increased and decreased binding of hnRNP L and the increased and decreased expression of seRNA-1. The authors draw a model that is consistent with this, but there is no indication in the model how such dual activities can be mechanistically explained.

Reviewer #2 (Remarks to the Author):

The authors provided a comprehensive transcriptome resource for myoblast and myotube by GRO-seq, PolyA+ and total RNA-seq. Some of bidirectionally transcribed eRNAs, such as seRNA-1 and seRNA-2, are induced by MyoD during myogenesis and promote myogenic differentiation in vitro and in vivo. Furthermore, the authors found that seRNA-1 controls the transcription of its neighboring gene through binding to hnRNPL and modulates hnRNPL occupancy at target promoters. Overall, the story is very interesting and should be published given if the following concerns can be properly

addressed.

Major concerns:
1. Using “stable/unstable” to classify eRNAs is misleading. Because the authors did not analyze their turnovers at all. Meta-gene analysis (Figure 1e) showed that the transcriptional levels (RPKM of GRO-seq and Pol II) of unstable eRNAs is ten-fold less than that of Bi-stable eRNAs, indicating the transcription but not RNA stability is the major effect.
2. Subcellular localization of seRNA-1 or seRNA-2? RNA FISH analysis of seRNA-1 showed an intensive focal site in nucleus but the majority of signals detected in cytoplasm. The authors should include a knockdown or knonckout control for these seRNAs. Otherwise, we cannot tell the specificity of FISH probes. In addition, it is better to perform co-staining with seRNA-1 DNA FISH in order to conclude that this seRNA binds to its own locus. Moreover, to achieve statistical significant, the authors should count more cells and indicate the proportion of cells showing the presented patterns. Furthermore, subcellular localization following by RT-PCR showed a significant amount of U1 detected in the cytoplasm, which is not right. U1 snRNA is exclusively localized in the nucleus. The authors should re-do this analysis. Lastly, what are the absolute numbers of seRNA-1 or 2 molecules per cell relative to hnRNPL? hnRNPL was mainly enriched in nuclei. What is the function of these two seRNAs in the cytoplasm?
3. Direct repressive role of hnRNPL on Mb expression?
The seRNA-1 tethering (Figure 6j-k) and seRNA-1 KD/KO (Figure 3f, 4k and 6f-g) led to increased occupancy of hnRNPL at Mb region and downregulation of Mb expression, which linked the increase of hnRNPL binding and decrease of Mb expression. Consistent with these observations, hnRNPL overexpression (Figure 6h) resulted in downregulation of Mb expression. However, hnRNPL KD by shRNA also led to a decrease of Mb expression, which suggested an indirect role of hnRNPL for Mb activation. Additional evidence, such as in vitro luciferase reporter assay, should be added to dissect the direct repressive role of hnRNPL from its indirect active role.
4. Role of eRNAs/hnRNPL as a general regulator of enhancers?
The authors speculated that eRNAs/hnRNPL could be a general regulator of enhancers. However, I am curious about percentages of eRNAs or seRNAs bound by hnRNPL in MB and MT cells. It is possible that highly transcribed eRNAs get more chance to be captured by hnRNPL. Could the authors address the binding specificity of hnRNPL on eRNAs?
5. To support the model that seRNA-1 directly modulates the dose of hnRNPL at Mb promoter, additional evidence is needed to clarify how seRNA-1 senses the dosage of hnRNPL and how hnRNPL is evicted from Mb promoter.
6. The authors concluded that seRNA-hnRNPL together activate transcription of the target Mb gene. Deletion of CAAA motif generates a partially loss-of-function mutant in terms of transcription attenuation of Mb, but it is also a gain-of-function mutant in terms of seRNA expression and hnRNP-L binding at the Mb promoter. What if knocking out the whole seRNA gene or the 5’ promoter regions of seRNA? How could a complete removal of seRNA-1 affect hnRNPL binding at the Mb promoter and the expression of Mb?

Minor concerns:
1. Page 7, line 6, “When overlapped with enhancer repertoire, it yielded a catalogue…”. Does those eRNAs identified by total RNA-seq but not polyA+ RNA-seq mean polyA- transcripts? Or this phenomenon just shows that polyA+ RNA-seq is insensitive for those modestly/lowly expressed eRNAs? In case those eRNAs are not polyadenylated, it’s interestingly to determine their biogenesis, subcellular localizations and functions from polyA+ eRNAs.
2. Page 10, line 7, “…eRNAs that are highly expressed in MB and MT”. The definition of highly expressed eRNAs in MB and MT need to be clarified.
3. Page 12, line 11, “Two seRNAs, seRNA-1 and seRNA-2 were selected ...”. The rationale to select seRNA-1 and seRNA-2 need to be clarified.
4. In Figure 3d, 30%~40% of U1 RNAs are localized in cytoplasm, which is inconsistent with previous report (Pessa et. al., PNAS, 2008). The increased U1 RNA in cytoplasm may be caused from nuclear leaking during fractionation.

5. Explain the causes of the inconsistence of RNA pull-down and RIP results, where seRNA-1/2 interacted with hnRNPK in RNA pull-down but this interaction was disappeared in RIP.
6. Quantify the molecules of seRNA-1 and hnRNPL in case seRNA-1 serves as molecular decoy preventing the overloading of hnRNPL.

Reviewer #3 (Remarks to the Author):

Zhao and colleagues presented some very noteworthy data concerning how enhancer RNAs (eRNAs) activate target gene transcription during myogenic differentiation. The authors categorized different eRNA subfamilies through integrating both RNA-seq and Gro-seq data in myoblasts and myotubes, and further demonstrated that MyoD is critical for the induction of eRNAs during the process of myoblasts differentiation into myotubes. MyoD-induced eRNAs (seRNA-1) interact with hnRNPL through a CAAA motif to activate cognate coding genes and promote myogenesis, and the interaction between eRNAs and hnRNPL was proposed to be a general mechanism in multiple cell lines. Finally, evidence was presented indicating that seRNA-1 modulates hnRNPL dosage and therefore RNA Pol II/H3K36me3 deposition at myoglobin (Mb) locus. Overall, the topic addressed by this study was undoubtedly interesting, the experiments were well designed and most of the conclusions drawn were supported by the data presented. Nevertheless, there are some concerns that need to be addressed to consolidate the conclusions.

Major points:
1. The authors classified eRNAs based on their stability through integrating both GRO-seq and RNA-seq data in myoblasts and myotubes, and demonstrated that signatures (histone markers, transcription factors etc.) associated with transcriptional activation were highly correlated with stability of eRNAs. As RNA transcription and stability are controlled in a distinct manner, it is therefore still unclear why these highly transcribed eRNAs are more stable than those with lower expression.
2. To strengthen the conclusion that seRNA-1 works through cis-regulation of neighboring genes, myoglobin (Mb) and apolipoprotein L6 (Apol6), to promote myogenesis, it is crucial to demonstrate that reintroduce Mb and Apol6 into cells can rescue the defects caused by seRNA-1 knockdown.
3. Several pieces of data presented in the manuscript casted doubt on hnRNPL being a bona-fide functional partner of eRNAs: all the bands, instead of the one pointed out by the authors, from antisense pulldown appeared to be less abundant compared to that from sense; knockdown of hnRNPL exhibited no effects on the transcriptional activation of neighboring genes of seRNA-2; the marginal effects of hnRNPL on transcription genome-wide (Fig. 5q) were not comparable to the wide spectrum of interaction between hnRNPL and eRNAs.
4. It is quite surprising that both knockdown of hnRNPL and removing of CAAA tract attenuated the expression of neighboring genes of seRNA-1, but simultaneously up-regulating seRNA-1, suggesting that it is unlikely the transcript itself (seRNA-1) important for regulating neighboring genes.

Minor points:
1. It is not appropriate to simply remove transcription units from gene regions when counting eRNAs as eRNAs are often found to be transcribed from intronic and exonic regions.
2. It is unclear why the eRNAs detected in GRO-seq experiments in Fig. 1a were predominantly from sense direction.
3. The exact number of neighboring genes which behaved in the same direction as eRNAs during the process of differentiation from myoblasts to myotubes should be included.
4. The specificity of FISH signals observed in Fig. 3b and 3c needs to be confirmed by simultaneously knocking down of eRNA of interest.
5. The authors need to comment on the fact that both seRNA-1 and seRNA-2 also strongly retrieved other proteins, such as PLEC, VIM and CKAP4.
6. It is unclear whether the region responsible for binding with hnRNPL in seRNA-2 also has a CAAA tract. It is also interesting to know whether a CAAA motif is enriched in all eRNAs associated with

hnRNPL.
7. It was believed that hnRNPL is involved in Pol II elongation and H3K36me3 deposition by interacting with P-TEFb and KMT3A, respectively. Indeed, Pol II elongation and H3K36me3 deposition were largely affected in the absence of hnRNPL. However, it is unclear whether P-TEFb and KMT3A also exhibited increased binding during the process of myoblasts differentiation into myotubes. Furthermore, the Pol II elongation and H3K36me3 deposition should also be examined on another neighboring gene of seRNA-1, Apol6.

Reviewer #4 (Remarks to the Author):

In their manuscript, Zhao et al used GRO-seq to measure nascent RNA in proliferating and in differentiating mouse C2C12 muscle cells. To identify enhancer RNAs, the GRO-seq results where then overlapped with the catalog of muscle enhancers that the authors have previously identified in the same conditions. Using total and PolyA+ RNA-seq and previously generated ChIP-seq datasets, the authors addressed the issues of transcript abundance, directionality and stability. Based on this initial characterization, the authors decided to narrow their study on eRNAs produced from super enhancers (seRNAs). Upon further characterization, the authors selected two seRNAs induced during myogenic differentiation (possibly in a MyoD-dependent fashion) for functional studies. seRNA-1 and 2 induction during muscle differentiation was confirmed in vitro using C2C12 and primary cells and in vivo during muscle development and regeneration. Importantly, both seRNAs were showed to be required for the expression of genes located close to the enhancers, as well as for the expression of the myogenic genes MyoD and MyHC and the in vitro differentiation of C2C12 cells. While the authors found that both seRNAs interact specifically with hnRNPL, the protein is important for mediating the activity of only seRNA-1 and only for the expression of the nearby gene Mb. In parallel, the authors reported data indicating that binding to eRNAs could be a general feature of hnRNPL. Based on these results, the authors propose that the eRNA-hnRNPL interaction represents a novel and general mechanism contributing to target mRNA activation.
While intriguing, the manuscript presents a number of critical aspects that need to be addressed to support the authors conclusions.

MAJOR POINTS

1. In their previous paper, the authors identified a total of 406 and 759 SEs together with 8389 and 11052 TEs in MB and MT, respectively. How can the number of teRNAs and seRNAs reported in the present manuscript be so much more than the actual number of TEs and SEs? Are the identified eRNAs in large part portions or isoforms of the same (larger) eRNA? Authors must address this discrepancy.

2. Without something to compare with, it is unclear how meaningful are the results of Figure 1 e-h. How abundant are the transcripts and the enrichments reported compared to known benchmarks? For example, it would be useful to have signals for known protein coding genes and lincRNAs which are known to be differentially expressed during myogenic differentiation as well as for untranscribed gene deserts. This would allow to compare the results reported in Figure 1 to known, previously characterized genomic entities in muscle cells.

3. Figure 1l is low resolution and the corresponding legend uninformative. What is it the area highlighted in yellow? The information provided in this Figure put into question the rationale used to identify SEs as the area indicated by the red bars does not differ much from adjacent areas.

4. About half of MB seRNAs of Supplementary Figure 1l is not differentially expressed or it is increased during myogenic differentiation. This raises concern that a large fraction of the seRNAs that the authors describe as more expressed in MB are instead not so. Furthermore, the same MB and MT

seRNAs of Figure 1m and Supplementary Figure 1l must be analyzed also during in vitro myogenic differentiation of freshly isolated satellite cells to support findings in primary cells. Also, it would be important to address the question of how many of the identified seRNAs are evolutionarily conserved in human muscle cells. Finally, at least for seRNA 1 and 2, the authors must determine if a human counterpart exists and if these human seRNAs are also required for myogenic differentiation.

5. I don't agree that the data of Figure 1 and Supplementary Figure 1 "demonstrate that dynamic SEs are prevalently transcribed in muscle cells". For this, the authors must address the issue of tissue/cells specificity in general for their seRNAs. What percentage of the identified eRNAs are muscle specific? This must be done in general, by comparing the authors NGS data to publicly available data. Also, for the candidates of Figure 1m and Supplementary Figure 1l, this must be done by performing RT-qPCR using RNA extracted from multiple tissues.

6. There are a couple of caveats with the results of Figure 2 e-l. First, the reported results could be simply secondary to a defect in myogenic differentiation by MyoD KO cells. Authors must show representative pictures and quantify the differentiation index, the fusion index and the number of nuclei per myofiber of C2C12 KO for MyoD compared to controls since this was not reported in their previous paper. Moreover, the authors show that MyoG is enriched at the eRNAs genomic regions and it is expressed similarly to eRNAs. Given that MyoG is directly regulated by MyoD, all the results of Figure 2 e-l could be due not by a direct activity of MyoD, but by MyoG. Authors must address this important mechanistic issue. For example, what is the effect of knocking down MyoG on the expression of the eRNAs?

7. I don't agree that the data of Supplementary Figure 3a support the "extraordinary cell/tissue specificity" of the analyzed seRNAs. Indeed, they are clearly expressed in several tissues (seRNA-1) or similarly expressed in hearth, limb and testis (seRNA-2).

8. The FISH and RT-qPCR results of Figure 3b-d don't really match. This is particularly true for seRNA-2, which by FISH seems mostly cytoplasmic. Moreover, it is curious that both seRNAs show a perinuclear cytoplasmic signal. Since the authors showed just a single nucleus, it is difficult to tell how general is this behavior. Is this consistently seen in every myonucleus? Does it match with any sub-cellular structure compartment (for example the ER)? RNA-FISH for a control protein coding gene and treatment with RNAse before the RNA-FISH for seRNAs must be provided to support the specificity of the RNA-FISH results.

9. Since the seRNAs are cytoplasmic, work by binding hnRNPL and are capable to regulate reporters containing the promoter of their targets, they should work in trans. Hence, I have some difficulty in understanding why their overexpression (particularly for seRNA-1 for which the model of Figure 7 implies an in trans interaction with hnRNPL) is ineffective. Important information/controls are missing. Have the seRNAs been overexpressed in myoblasts or myotubes? Are the ectopically expressed seRNAs localized similarly to the endogenous seRNAs? Are the ectopically expressed seRNAs associated to hnRNPL?

10. The results of Supplementary Figure 3i, j suggest that seRNA-1 and 2 work in trans by affecting the activity of the MyoG and MyHC promoters. Where these genomic regions identified in the seRNA-1 ChIRP? Is hnRNPL involved in this activity of seRNA-1 and 2? If not, what is the mechanism through which seRNA-1 and 2 regulate MyoG, MyHC and myogenic differentiation?

11. MyoG expression in Fig. 3i, l is barely visible, while it must be strongly induced by myogenic differentiation and (differently from the results of Fig. 3h, k) there is no obvious effect of seRNA-1/2 knockdown on MyoG expression.

12. The results of Fig. 3o, p are intriguing as they suggest that the seRNAs might have a biological relevance in vivo. If the authors would manage to demonstrate this, their story would be significantly

improved. Are mice treated with seRNA-1 or seRNA-2 defective in muscle regeneration? Authors must show histology of muscles treated with BaCl2 plus seRNA-1 or seRNA-2 siRNAs and controls siRNAs and quantify centrally nucleated fibers, muscle fiber cross-sectional area, muscle infiltrate, muscle fibrosis and adipose tissue deposition. A role for seRNA-1 or seRNA-2 in vivo in muscle regeneration will strongly support the biological relevance of the eRNAs.

13. The fact that hnRNPL has no role in the activity of seRNA-2 raises strong concern regarding the general biological relevance of the interaction of hnRNPL with eRNAs as proposed by the authors. seRNA-2 is important for the regulation of nearby genes, myogenic genes and muscle differentiation but none of this is due to its interaction with hnRNPL. Consequently, there is concern that most of the hnRNPL/eRNA interactions that the authors identified by CLIP could have limited biological relevance. All in all, the authors have supported a role for hnRNPL interaction with eRNAs only for seRNA-1 and this is apparently limited only to the regulation of Mb and Apol6 gene expression, not for the seRNA-1 role in myogenic differentiation.

14. Is knockdown of hnRNPL reducing the expression of MyoG and MyHC like the knockdown of seRNA-1? Is knockdown of hnRNPL in C2C12 and in vivo causing myogenic defects similar to those caused by seRNA-1 knockdown? This is key to understand the biological relevance of hnRNPL for the biology of the seRNA and the mechanism proposed by the authors.

15. I don't agree that the fact that regions positive for hnRNPL CLIP tags show higher GRO-seq reads than those with no CLIP signal (Fig. 5f), demonstrate that "hnRNPL binding with eRNAs is a transcriptome-wide event". This is probably simply a reflection that the inefficiency of CLIP, like any IP approach, allows to detect only relatively stable and abundant transcripts, independently of the fact that they belong to enhancer regions.

16. It is surprising that, despite they used them to identify hnRNPL, the authors did not show CLIP-seq signal for the genomic regions of seRNA-1 and 2 in Figure Fig. 5i. Where seRNA-1 and 2 identified by CLIP-seq? How are the CLIP-seq tags in the seRNA-1 and 2 genomic loci?

17. It is unclear if the genes affected by hnRNPL knockdown are enriched for genes regulated during myogenesis and (more importantly) for the same gene categories enriched in the seRNAs knockdown ((Supplementary Figure 1d). Again, the authors must strengthen the link and relevance of the seRNAs-hnRNPL circuit for myogenesis.

18. I don't agree that the results of Figure 5 "support the notion that hnRNPL-eRNA interaction is critical in regulating target genes expression". seRNA-1 is the only clear example provided by the authors that the interaction between hnRNPL and one eRNA is involved in the regulation of nearby genes. For seRNA-2, despite a specific binding with hnRNPL, there is no role of hnRNPL in the regulation of the seRNA-2 targets. While the authors have provided a genome-wide correlation between hnRNPL-eRNA and gene expression, this remains a correlation with no other clear example (beside seRNA-1) of a direct role of the hnRNPL-eRNA interaction in the regulation of specific targets. Moreover, the authors failed to show that the interaction between hnRNPL and eRNAs is functionally relevant for muscle differentiation.

19. Authors must provide much improved results for the co-IP of Figure 6d, with clearly distinguishable bands and not smeared signals.

20. The results of Figure 6c, f, g are very difficult to reconcile with those of Figure 1m, 3f and 4f. The level of seRNA-1 increases of nearly 100 folds between MB and MT. The level of hnRNPL enrichment at the Mb locus also increases between MB and MT. Accordingly, knockdown of seRNA-1 or hnRNPL decrease Mb gene expression. If seRNA-1 works by inhibiting hnRNPL recruitment to the Mb locus, then why hnRNPL enrichment on the Mb locus is not higher in MB in which seRNA-1 levels are low? Why hnRNPL enrichment at the Mb locus increases in MT despite a ~100 fold increase in seRNA-1

level? How authors envision the double/discordant activity of seRNA-1 to take place mechanistically? How can this mechanism regulate selectively Mb, given that Apol6 is not affected by seRNA-1 tethering? Can the authors exclude off target effects of seRNA-1 knockdown, hnRNPL overexpression or seRNA-1 tethering? For example, what is the control for Figure 6 j-k? It appears that the authors used untransfected cells or cells transfected with an empty vector. Instead, the appropriate control would be to use a seRNA-1 lacking the hnRNPL binding sequence. This is the best control to support a role for hnRNPL in the activity depicted as opposed to a role of an unknown RNA-binding protein or a generic transcription interference caused by CAS9 blocking access of the transcriptional machinery by steric interference (Cell. 2013 Feb 28; 152(5): 1173–1183). Can the author show that the decreased Mb expression caused by seRNA-1 tethering is specifically due to direct hnRNPL recruitment to the Mb locus by seRNA-1? Why this complex mechanism would be in place to regulate selectively Mb?

MINOR

- The authors did not provided indication of the timepoint/differentiation level of MT used for GRO-seq.

Point to point response

Reviewer #1:

This manuscript is an exceptionally dense analysis of the potential role of hnRNP L in the function of super-enhancers. There are certain to be some interesting findings here, but the deluge of often-subtle data detracts from the conclusiveness and clarity of the work and the ultimate model in the final figure is difficult to rationalize. The manuscript really needs to be reworked with focus on presenting just the critical data to support the main point (quality over quantity). The following specific concerns minimally need to be addressed before the manuscript can be fully comprehended and judged.

Thanks for the comment. We agree that the focus of the study is the dissection of eRNA functional mechanism thus some data on the categorization of eRNAs in Fig. 1 and the phenotypically dissection in Fig. 3 may have distracted from the conclusiveness and clarity of the work. We have now removed some panels of the original Fig. 1 from the main figure. However, we hesitated to remove other data considering the large number of comments we received from other reviewers concerning those. More detailed suggestions from the reviewer on what to remove will be greatly appreciated.

1.1. The term "seRNA" is used in both the abstract and introduction with no definition.

We apologize for not giving definition for seRNA. We have now added the definition in both abstract and introduction on Pages 2, 4 and 7. Briefly, seRNAs are eRNAs generated from super enhancers (SEs) instead of typical enhancers (TEs).

1.2. Even if they are statistically significant, the differences in figures such as 1b and 5r are too subtle to be biologically meaningful. This raises concerns regarding the model and physiologic relevance of what is being investigated.

Thanks for the critical comment. In the original Fig. 1b, the neighboring genes were set within ± 150 kb region flanking the eRNA genomic locus according to commonly used criteria (Ørom et al, Cell, 2010). We have now changed the window to ± 100 kb and found the fold change and confidence level both increased (Revised Fig. 1b). We speculate that the correlation between eRNA and neighbor gene expression will be even more significant if we could infer the authentic target genes of eRNA using genome-wide enhancer-promoter interaction profile; this, unfortunately, is currently unavailable in muscle cells. The difference in Fig. 5r is indeed subtle; we agree that more solid evidence is needed to extend our model to genome-wide eRNAs spectrum. We have thus revised the manuscript on Pages 17, 19 and 22 to tone down our point. Please also see our answer to Reviewr 2's comment 2.4, Reviewer 3's comment 3.3 and Reviewer 4's comment 4.13.

1.3. Why were seRNA-1 and seRNA-2 selected for further study based on Fig 1 and 2? No justification is given for why these, among all the others, are of most interest.

Thanks for the excellent comment. We apologize for not giving the justification why seRNA-1 and seRNA-2 were selected. We have now revised the text on Page 10 to include the justification. In brief, seRNA-1 and seRNA-2 were selected because they are among the highest induced

seRNAs in myotube vs myoblast (Supplementary Fig. 5a). We believe these seRNAs can potentially promote myoblast differentiation thus worthy further investigation.

1.4. Do the siRNAs deplete the nuclear pool of seRNA-1 and -2? This is critical for the model, but no evidence is given. Since most of the seRNAs are in the cytoplasm, looking at total isn't valid.

Thanks for the great comment. We agree it is important to show the depletion of seRNA-1 and -2 in nuclear pool. We have now repeated the siRNA knockdown experiments followed by cellular fractionation assay to detect the nuclear level of seRNA-1 and seRNA-2. We indeed observed their decrease in the nuclear pool. The data can be found in Supplementary Fig. 6a and Page 12 of the revised text.

1.5. What is the specificity of the impact of the knock-downs? That is, does siRNA against seRNA-1 impact expression of Atp1a1 and Igsf3?

Thanks for the good point. We have now performed the suggested experiment and found seRNA-1 knockdown did not decrease the expression of Atp1a1 and Igsf3; similarly, seRNA-2 knockdown had no impact on the expression of Mb and Apol6. The data can be found in Supplementary Fig. 6b and Page 12 of the revised text.

1.6. In the binding experiment in Figure 4, if known eRNA binding partners were not found, how is the validity of the experiment confirmed?

Thanks for the critical comment. We agree it is important to confirm the validity of the binding experiment. We have now performed binding experiment with a positive control, Linc-p21 RNA, which is known to bind with hnRNPK (Huarte et al, Cell, 2010; Bao et al, Cell Res, 2015). Indeed, RNA pull down with Linc-p21 retrieved hnRNPK confirming the efficiency of our procedure. Also, the same procedure has also been used in several of our previous publications (Zhou et al., Nat Commun., 2015; Wang et al., Cell Res., 2015; Chen et al., Cell Discov., 2017). Additionally, we have already presented results from other experiments including RIP, deletion mapping etc. in Fig. 4 to support the specificity of seRNA-1 or -2 interaction with hnRNPL. The newly added data can be found in Supplementary Fig. 7a and Page 14 of the revised text.

1.7. The fact that depletion of hnRNP L doesn't impact seRNA-2 function clearly demonstrates that binding of hnRNP L (which is more specific for seRNA-2 than -1), does not imply function. This completely undermines the value of the CLIP experiment in Figure 5. Additionally, no explanation is given for why seRNA-2 might not involve hnRNP L.

Thanks for the critical comment. It is true that our model of seRNA/hnRNPL synergism does not seem to apply to seRNA-2 as knockdown of hnRNPL did not have any impact on seRNA-2 targets. We speculate that the specificity of seRNA-2 interaction with hnRNPL is not strong enough compared with seRNA-1 as seRNA-2 has only one CACACA motif while seRNA-1 has eight copies of CAAA tract. Nevertheless, we believe that we have provided enough evidence to show our mechanistic model applies well on seRNA-1 and may very well likely to extend beyond seRNA-1. For example, we did observe higher CLIP-seq tags in eRNAs are associated with higher expression level of eRNAs neighboring genes (Fig. 5p) and neighboring genes with eRNAs binding are more vulnerable to hnRNPL knockdown (Fig. 5r). In addition, we also

performed analyses in HeLa and mESC cells to show the potential connection between eRNA/hnRNPL (Fig. 5j-o). Like all other studies, we could only concentrate on one seRNA to provide thorough dissection of the mechanism; it will be necessary to include more eRNA candidates for testing in the future. We have revised the text on Pages 17, 19 and 22 to include the above discussion. In addition, Reviewer 2, 3 and 4 all raised a similar concern; please also see our answers to their comments 2.4, 3.3 and 4.13.

1.8. The logic circuit doesn't fit. Loss of seRNA-1 reduces target expression and increases binding of hnRNP L to seRNA-1 target genes, Loss of hnRNP L increases seRNA-1 expression and decreases seRNA-1 target genes. So target gene expression is reduced upon both the increased and decreased binding of hnRNP L and the increased and decreased expression of seRNA-1. The authors draw a model that is consistent with this, but there is no indication in the model how such dual activities can be mechanistically explained.

Thanks for the critical comment. We agree that the mechanistic insight behind the model was not sufficient in the original submission. Your comment together with the other two reviewers' (Reviewer 2 comment 2.5 and Reviewer 4 comment 4.20) has driven us to explore deeper into the mechanism of how seRNA-1/hnRNPL interaction regulates Mb expression. First, from the collective data we have so far we believe that the hnRNPL dosage on the target promoter is critical to induce gene expression and seRNA-1 binding with hnRNPL functions to prevent the overloading of hnRNPL. Therefore, loss of seRNA-1 binding by knockdown or removing CAAA led to aberrant enrichment of hnRNPL on the target locus and inhibited the gene activation (Figs. 3f, 4k, 6f, 6g), which can be phenocopied by overexpressing hnRNPL in cells (Fig. 6h). Tethering a seRNA-1 also caused overdose of hnRNPL on the target locus thus similar transcriptional inhibition (Fig. 6i-k). Second, we believe the transcriptional activating role of hnRNPL is mediated by its interaction with Pol II elongation machinery according to prior reports (Giraud et al., PNAS, 2014; Huang et al., Mol Cell, 2012; Yuan et al., JBC, 2009). Indeed we showed that hnRNPL interacts with CDK9 and CCNT1 (Fig. 6d) and concordant enrichment of all three proteins were seen on the target promoter in myotube (Fig. 6c); interestingly, seRNA-1 knockdown led to increased binding of hnRNPL and CDK9 but decreased CCNT1 binding (Fig. 6f), suggesting that the complex interaction of hnRNPL with transcriptional machinery needs to be further investigated in the next chapter of the study. Accordingly, we have now revised our model in Fig. 7 and the text on Page 21.

<u>Reviewer #2:</u>

The authors provided a comprehensive transcriptome resource for myoblast and myotube by GRO-seq, PolyA+ and total RNA-seq. Some of bidirectionally transcribed eRNAs, such as seRNA-1 and seRNA-2, are induced by MyoD during myogenesis and promote myogenic differentiation in vitro and in vivo. Furthermore, the authors found that seRNA-1 controls the transcription of its neighboring gene through binding to hnRNPL and modulates hnRNPL occupancy at target promoters. ***Overall, the story is very interesting and should be published given if the following concerns can be properly addressed.***

Major concerns:

2.1. Using "stable/unstable" to classify eRNAs is misleading. Because the authors did not analyze their turnovers at all. Meta-gene analysis (Figure 1e) showed that the transcriptional levels (RPKM of GRO-seq and Pol II) of unstable eRNAs is ten-fold less than that of Bi-stable eRNAs, indicating the transcription but not RNA stability is the major effect.

Thanks for the excellent comment. We agree using "stable/unstable" is misleading considering we did not analyze eRNA turnovers. These terms only refer to whether an eRNA can be detected in steady state RNA-seq or GRO-seq only. We have now revised the manuscript on Page 23 to give a better description for the terms.

2.2. Subcellular localization of seRNA-1 or seRNA-2? RNA FISH analysis of seRNA-1 showed an intensive focal site in nucleus but the majority of signals detected in cytoplasm. The authors should include a knockdown or knonckout control for these seRNAs. Otherwise, we cannot tell the specificity of FISH probes. In addition, it is better to perform co-staining with seRNA-1 DNA FISH in order to conclude that this seRNA binds to its own locus. Moreover, to achieve statistical significant, the authors should count more cells and indicate the proportion of cells showing the presented patterns. Furthermore, subcellular localization following by RT-PCR showed a significant amount of U1 detected in the cytoplasm, which is not right. U1 snRNA is exclusively localized in the nucleus. The authors should re-do this analysis. Lastly, what are the absolute numbers of seRNA-1 or 2 molecules per cell relative to hnRNPL? hnRNPL was mainly enriched in nuclei. What is the function of these two seRNAs in the cytoplasm?

Thanks for all the excellent comments/questions. We agree FISH is the key to show seRNA localization. We have now re-evaluated our original FISH procedure and found its specificity may be questionable. After many trials, we have finally optimized our FISH assay adopting a published method (Shaffer et al., Plos One, 2013), in which hybridization was performed with a high probe concentration and shortened time. This method dramatically reduced false background signal and improved accuracy. In the assay as suggested we have included the following controls: 1) no probe control, 2) RNase A treatment control, 3) Malat1 positive control and 4) staining in both MB and MT stages to see whether the signals increased during differentiation. Our newly acquired FISH results (Fig. 3b, c and Supplementary Fig. 5c) indicated seRNA-1 and seRNA-2 localize in both nucleus and cytoplasm of C2C12 cells; and they both form nuclear foci presumably on the site of transcription or action. As suggested, to achieve statistical significant, we have also counted more cells and found the percentage of seRNA-1 positive cells increased from 4% to 74% in MT vs MB, and from 10% to 48% for seRNA-2 (Fig. 3b, c). We also agree it is a good idea to do co-staining of seRNA and its DNA loucs to show the seRNA localizes to its own locus. Unfortunately, after repeated tries of the co-staining, we were not able to succeed in retaining RNA signal after DNA FISH. Nevertheless, our ChIRP-PCR result (Fig. 6b) indeed showed seRNA-1 binds with its own locus, which may explain the formation of some foci in FISH.

We agree that our original cell fractionation assay may not be well performed; there may be nuclear leaking during fractionation to cause U1 detection in cytoplasm. To this end, we have repeated the assay to show that U1 nuclear portion accounts for ~5.9% of total U1 signal in MB and ~15.5% in MT (Revised Fig. 3d), comparable to the level shown in previous report (Pessa et al., PNAS, 2008). In this batch of data, we found that seRNA-1 had a comparable level between nuclear and cytoplasmic portion in MT and seRNA-2 is slightly enriched in nuclear portion. We

have included the above described results in Fig. 3d and revised the corresponding text on Page 11.

We agree it is a good idea to quantify the absolute numbers of seRNA-1 or 2 molecules per cell relative to hnRNPL. The same comment was also raised in your comment 2.12. We have now quantified the copy number for seRNA-1 and seRNA-2 following the procedure from a previous report (Li et al., Nature, 2013). The results showed that seRNA-1 is present at levels of ~0.12 copy/cell in MB, and ~7.97 copies/cell in MT (DM day 2); seRNA-2 is around 2.44 copies/cellin MB and 10.04 copies/cell in MT (Supplementary Fig. 5d, e). The copy numbers appear to be low for exerting the proposed interaction with hnRNPL, we reason that the local concentration of the molecule on the nuclear foci (as revealed by FISH in Fig. 3b, c) is high enough to facilitate the interaction with hnRNPL and subsequent action on target locus. For hnRNPL, we performed IF staining and demonstrated that the protein is abundantly expressed and localized in nucleus in MB while expressed both in both cytoplasm and nucleus in MT (Supplementary Fig. 9h). We also attempted the co-staining of hnRNPL protein and seRNA-1 with the aim to see whether they co-localize in the nuclear but unfortunately did not succeed after repeated tries. Until now we have not been able to find any method to quantify a protein molecule and will be open to any suggestion from the reviewer. We have included the above described results and revised the corresponding text on Pages 11 and 20.

Lastly, it is a great question to ask about the function of these two seRNAs in cytoplasm. We agree the presence of these seRNAs in cytoplasm may infer some functional roles beyond transcriptional regulation at chromatin level. However, due to the time limitation in this study we could only focus on dissecting their nuclear roles while leaving the cytoplasmic roles to the next chapter. By wide speculation, we can imagine maybe in cytoplasm the seRNAs could also be binding with hnRNPL to facilitate the latter's post-transcriptional functions in RNA processing or translational regulation; analogously, another eRNA, ncRNA-a7 was shown to act as an enhancer and regulate Snail transcription in cis and mediate translational suppression on E-cadherin in cytoplasm through interaction with a ribonucleoprotein (RNP) complex, consisting of hnRNP K, FXR1, FXR2, PUF60 and SF3B3 (Ørom et al, Cell, 2010; Gumireddy et al., EMBO J, 2013).

2.3. Direct repressive role of hnRNPL on Mb expression?
The seRNA-1 tethering (Figure 6j-k) and seRNA-1 KD/KO (Figure 3f, 4k and 6f-g) led to increased occupancy of hnRNPL at Mb region and downregulation of Mb expression, which linked the increase of hnRNPL binding and decrease of Mb expression. Consistent with these observations, hnRNPL overexpression (Figure 6h) resulted in downregulation of Mb expression. However, hnRNPL KD by shRNA also led to a decrease of Mb expression, which suggested an indirect role of hnRNPL for Mb activation. Additional evidence, such as in vitro luciferase reporter assay, should be added to dissect the direct repressive role of hnRNPL from its indirect active role.

Thanks for the comment. We agree that more mechanistic insight is needed to solidify our model involving seRNA/hnRNPL. We have performed the suggested reporter assay and found that overexpression of hnRNPL in C2C12 cells, even a low dose of hnRNPL caused reduction in Mb promoter activity (Supplementary Fig. 9f), suggesting over-dosage of hnRNPL may repress Mb expression directly. In addition, tethering seRNA-1 to either seRNA-1 promoter or Mb promoter

also decreased the Mb expression (Fig. 6j, k), again suggesting the overdosed hnRNPL binding impedes Mb expression. Taken together, our data suggest that the correct hnRNPL dosage on the target promoter is critical to activate Mb gene expression and the transcriptional activating role of hnRNPL is mediated by its interaction with Pol II elongation machinery according to prior reports (Giraud et al., PNAS, 2014; Huang et al., Mol Cell, 2012; Yuan et al., JBC, 2009). Indeed we showed that hnRNPL interacts with CDK9 and CCNT1 (Fig. 6d) and concordant enrichment of all three proteins were seen on the target promoter in myotube (Fig. 6c). Loss of hnRNPL led to disruptive binding of these transcriptional machinery (Fig. 6e) and Mb reduction. Please also see our answer to your comment 2.5 below.

2.4. Role of eRNAs/hnRNPL as a general regulator of enhancers?
The authors speculated that eRNAs/hnRNPL could be a general regulator of enhancers. However, I am curious about percentages of eRNAs or seRNAs bound by hnRNPL in MB and MT cells. It is possible that highly transcribed eRNAs get more chance to be captured by hnRNPL. Could the authors address the binding specificity of hnRNPL on eRNAs?

Thanks for the comment. We have now analyzed our available CLIP-seq from MT cells and found the percentage of all eRNAs bound by hnRNPL is limited (5.49%); only 6.36% for teRNAs and 4.57% for seRNAs respectively. Nevertheless, we did observe higher number of CLIP-seq tags in eRNAs are associated with higher expression level of the eRNA neighboring genes (Fig. 5p) and neighboring genes with eRNAs binding are more vulnerable to hnRNPL knockdown (Fig. 5r), implying a potential link between eRNA/ hnRNPL interaction in general. Still, we agree that more solid evidence is needed to extend this model to genome-wide eRNAs spectrum. We have thus revised the manuscript on Pages 17, 19 and 22 to tone down our point. Please also see our answer to Reviewer 1's comment 1.7, Reviewer 3's comment 3.3 and Reviewer 4's comment 4.13.

Also, to exclude the possibility that highly transcribed eRNAs get more chance to be captured by hnRNPL in CLIP-seq, we found that there is no positive correlation when plotting the hnRNPL CLIP-seq tags against eRNA expression level (Supplementary Fig. 8c and Page 17 of the text); this to some extend indicated the specificity of hnRNPL binding.

2.5. To support the model that seRNA-1 directly modulates the dose of hnRNPL at Mb promoter, additional evidence is needed to clarify how seRNA-1 senses the dosage of hnRNPL and how hnRNPL is evicted from Mb promoter.

Thanks for the comment. Your comment together with the other two reviewers' (Reviewer 1 comment 1.8 and Reviewer 4 comment 4.20) has driven us to explore deeper into the mechanism of how seRNA-1/hnRNPL interaction regulates Mb expression. First, from the collective data we have so far we believe that the hnRNPL dosage on the target promoter is critical to induce gene expression and seRNA-1 binding with hnRNPL functions to prevent the overloading of hnRNPL. Therefore, loss of seRNA-1 binding by knockdown or removing CAAA led to aberrant enrichment of hnRNPL on the target locus and inhibits the gene activation (Figs. 3f, 4k, 6f, 6g), which can be phenocopied by overexpressing hnRNPL in cells (Fig. 6h). Tethering a seRNA-1 also caused overdose of hnRNPL on the target locus thus similar transcriptional inhibition (Fig. 6i-k). The dosage control is probably executed stochastically through the molecular ratio between seRNA-1 and hnRNPL binding. As determined in Supplementary Fig. 5e, a relatively

low number of seRNA-1 molecules were found in MT cells which can bind with a certain number of hnRNPL proteins on the target locus. More solid evidence on this can be obtained in the future in the next chapter of the study. Second, we believe the transcriptional activating role of hnRNPL is mediated by its interaction with Pol II elongation machinery according to prior reports (Giraud et al., PNAS, 2014; Huang et al., Mol Cell, 2012; Yuan et al., JBC, 2009). Indeed we showed that hnRNPL interacts with CDK9 and CCNT1 (Fig. 6d) and concordant enrichment of all three proteins were seen on the target promoter in myotube (Fig. 6c); interestingly, seRNA-1 knockdown led to increased binding of hnRNPL and CDK9 but decreased CCNT1 binding, suggesting that the complex interaction of hnRNPL with transcriptional machinery needs to be further investigated in the next chapter of the study. Accordingly, we have now revised our model in Fig. 7 and the text on Page 21.

2.6. The authors concluded that seRNA-hnRNPL together activate transcription of the target Mb gene. Deletion of CAAA motif generates a partially loss-of-function mutant in terms of transcription attenuation of Mb, but it is also a gain-of-function mutant in terms of seRNA expression and hnRNP-L binding at the Mb promoter. What if knocking out the whole seRNA gene or the 5' promoter regions of seRNA? How could a complete removal of seRNA-1 affect hnRNPL binding at the Mb promoter and the expression of Mb?

Thanks for the comment. We agree that theoretically knocking out the whole seRNA-1 gene or the 5' promoter region can provide additional insights. However, we reason this may disrupt important regulatory regions such as enhancers for Mb gene, making it difficult to distinguish the outcome of deleting seRNA-1 or the DNA region. To provide additional evidence, we have now established another mutant cell line in which a short sequence was removed in the second exon of seRNA-1 while leaving CAAA tact intact. The removal of this region did not reduce the expression of Mb or change the hnRNPL binding at Mb locus (Supplementary Fig. 9c-e), strengthening the importance of CAAA tract and the interaction between seRNA-1 and hnRNPL in regulating Mb expression. We have included the above results and revised the corresponding text on Page 20.

Minor concerns:

2.7. Page 7, line 6, "When overlapped with enhancer repertoire, it yielded a catalogue…". Does those eRNAs identified by total RNA-seq but not polyA+ RNA-seq mean polyA- transcripts? Or this phenomenon just shows that polyA+ RNA-seq is insensitive for those modestly/lowly expressed eRNAs? In case those eRNAs are not polyadenylated, it's interestingly to determine their biogenesis, subcellular localizations and functions from polyA+ eRNAs.

Thanks for the interesting comment. We found that some eRNAs identified by total RNA-seq and absent in PolyA+ RNA-seq are indeed polyA- but this is not always the case. We agree that it is important to ponder on the biogenesis, subcellular localization and functions of PolyA + vs. PolyA- eRNAs. However we argue this may be beyond the focus scope of this study and should be investigated in the next chapter of our study.

2.8. Page 10, line 7, "…eRNAs that are highly expressed in MB and MT". The definition of highly expressed eRNAs in MB and MT need to be clarified.

We apologize for the confusion caused by the unclear writing. To define the eRNAs highly expressed in MB and MT, we compared their expression in MB and MT. Then we used log_2 (Fold change) > 2 or <-2 with adjusted p-value < 0.05 as cutoff to select eRNAs that are highly expressed in MT and MB respectively. We have now revised the manuscript on Page 9 to clarify on this point.

2.9. Page 12, line 11, "Two seRNAs, seRNA-1 and seRNA-2 were selected ...". The rationale to select seRNA-1 and seRNA-2 need to be clarified.

Thanks for the suggestion. As pointed out in the answer to Reviewer 1's comment 1.3, we have now revised the text on Page 10 to include the rationale. In brief, seRNA-1 and seRNA-2 were selected out because they are among the highest induced seRNAs in MT vs MB. We believe these seRNAs can potentially promote myoblast differentiation thus became the focus of our study. The data can be found in Supplementary Fig. 5a and Page 10 of the revised text.

2.10. In Figure 3d, 30%~40% of U1 RNAs are localized in cytoplasm, which is inconsistent with previous report (Pessa et. al., PNAS, 2008). The increased U1 RNA in cytoplasm may be caused from nuclear leaking during fractionation.

Thanks for the critical comment. As pointed out in the answer to the above Comment 2.2, we have now repeated the assay to show that U1 nuclear portion accounts for ~5.9% of total U1 signal in MB and ~15.5% of total U1 level in MT (Fig. 3d); this is comparable to the level shown in previous report (Pessa et al., PNAS, 2008).

2.11. Explain the causes of the inconsistence of RNA pull-down and RIP results, where seRNA-1/2 interacted with hnRNPK in RNA pull-down but this interaction was disappeared in RIP.

Thanks for the comment. The discrepancy between these two results can be explained by distinct conditions applied in the two assays. Briefly, RIP experiment was performed in a native condition without crosslinking and the outcome depends on the kinetic properties of the RNA-protein interaction, as well as relative abundances of the RBP and its RNA targets (Wheeler et al., Wiley Interdiscip Rev RNA., 2017). We think the loss of the detection of seRNA-1 and seRNA-2 in the RIP experiments may be due to the stringency of the wash conditions or low affinity between them and hnRNPK in vivo. We have revised text on Page 15 to include the above point.

2.12. Quantify the molecules of seRNA-1 and hnRNPL in case seRNA-1 serves as molecular decoy preventing the overloading of hnRNPL.

Thanks for the comment. We agree it is a good idea to quantify both molecules. The same comment was also raised in your comment 2.2. We have now quantified the copy number for seRNA-1 and seRNA-2 following the procedure from a previous report (Li et al., Nature, 2013). The results showed that seRNA-1 is present at levels of ~0.12 copy/cell in MB, and ~7.97 copies/cell in MT (DM day 2); seRNA-2 is around 2.44 copies/cell in MB and 10.04 copies/cell in MT (Supplementary Fig. 5d, e). The copy numbers appear to be low for exerting the proposed interaction with hnRNPL, we reason that the local concentration of the molecule on the nuclear foci (as revealed by FISH in Fig. 3b, c) is high enough to facilitate the interaction with hnRNPL and subsequent action on target locus.

Reviewer #3:

Zhao and colleagues presented some very noteworthy data concerning how enhancer RNAs (eRNAs) activate target gene transcription during myogenic differentiation. The authors categorized different eRNA subfamilies through integrating both RNA-seq and Gro-seq data in myoblasts and myotubes, and further demonstrated that MyoD is critical for the induction of eRNAs during the process of myoblasts differentiation into myotubes. MyoD-induced eRNAs (seRNA-1) interact with hnRNPL through a CAAA motif to activate cognate coding genes and promote myogenesis, and the interaction between eRNAs and hnRNPL was proposed to be a general mechanism in multiple cell lines. Finally, evidence was presented indicating that seRNA-1 modulates hnRNPL dosage and therefore RNA Pol II/H3K36me3 deposition at myoglobin (Mb) locus. ***Overall, the topic addressed by this study was undoubtedly interesting, the experiments were well designed and most of the conclusions drawn were supported by the data presented.*** *Nevertheless, there are some concerns that need to be addressed to consolidate the conclusions.*

Major points:

3.1. The authors classified eRNAs based on their stability through integrating both GRO-seq and RNA-seq data in myoblasts and myotubes, and demonstrated that signatures (histone markers, transcription factors etc.) associated with transcriptional activation were highly correlated with stability of eRNAs. As RNA transcription and stability are controlled in a distinct manner, it is therefore still unclear why these highly transcribed eRNAs are more stable than those with lower expression.

Thanks for the comment. As also pointed out by Reviewer 2 comment 2.1, using “stable/unstable” to classify eRNAs could be misleading because we did not analyze their turnovers. These terms only refer to whether an eRNA can be detected in steady state RNA-seq or GRO-seq only. We agree in the future it may be necessary to further explore the biogenesis, stability etc to explain why highly transcribed eRNAs are more stable than those with lower expression. As suggested by other existing studies, it is likely that RNA synthesis rate and decay rate are coupled, which can be mediated by core RNA Pol II proteins, promoters, transcription factors, decay factors, etc (Das et al., Microb Cell., 2017; Bellofatto et al., Cell, 2011; Bregman et al., Cell, 2011; Trcek et al., Cell, 2011; Haimovich et al., Cell, 2013; Pefanis et al., Cell, 2015). We have revised the manuscript on Page 23 to give a better explanation for the term.

3.2. To strengthen the conclusion that seRNA-1 works through cis-regulation of neighboring genes, myoglobin (Mb) and apolipoprotein L6 (Apol6), to promote myogenesis, it is crucial to demonstrate that reintroduce Mb and Apol6 into cells can rescue the defects caused by seRNA-1 knockdown.

Thanks for the critical comment. As suggested, we have now over-expressed Mb and Apol6 into cells with seRNA-1 stable knockdown and evaluated myogenic markers expression. As shown in Supplementary Fig. 6k, we found reintroducing Mb or Apol6 into the cells did not rescue the delayed myogenesis caused by seRNA-1 knockdown. We reason that in addition to activating the late myotube genes Mb and Apol6 to impact on myotube structure and contractile activity, seRNA-1 may also somehow regulate early myogenic genes such as Myogenin to impact on myogenic differentiation. Therefore, overexpression of Mb or Apol6 may only rescue the myotube defect in a late stage but not early differentiation eg. Myogenin expression. Altogether

our data seem to suggest that additional mechanism may exist to mediate seRNA-1 regulation of myogenesis eg. the Myogenin expression and this may not even involve hnRNPL. In the current study, our focus is to dissect how it regulates Mb transcription synergistically with hnRNPL; it will be interesting to explore additional mechanisms in the future. We have revised the text on Pages 13 and 24 to include the point. Please also see our answer to comment 4.10 from reviewer 4 who has a related comment.

3.3. Several pieces of data presented in the manuscript casted doubt on hnRNPL being a bona-fide functional partner of eRNAs: all the bands, instead of the one pointed out by the authors, from antisense pulldown appeared to be less abundant compared to that from sense; knockdown of hnRNPL exhibited no effects on the transcriptional activation of neighboring genes of seRNA-2; the marginal effects of hnRNPL on transcription genome-wide (Fig. 5q) were not comparable to the wide spectrum of interaction between hnRNPL and eRNAs.

Thanks for the critical comment. We believe we have provided solid evidence on how seRNA-1/hnRNPL interaction regulates Mb expression and provided some evidence to demonstrate the possible generalization of our model in muscle cells as well as other cells. As pointed out in the answer to Reviewer 2's comment 2.4 and Reviewer 1' comment 1.7, by analyzing our available CLIP-seq from MT cells we found the percentage of eRNAs bound by hnRNPL is indeed limited (5.49%). Nevertheless, we did observe higher CLIP-seq tags in eRNAs are associated with higher expression level of the eRNA neighboring genes (Fig. 5p) and neighboring genes with eRNAs binding are more vulnerable to hnRNPL knockdown (Fig. 5r), implying a potential link between eRNA/ hnRNPL interaction and neighboring genes expression in general. In addition, we also performed analyses in Hela and mESC cells to show the potential connection between eRNA and hnRNPL (Fig. 5j-o). That said, we agree that the wide spectrum of interaction needs to be solidified with more evidence in the future. We have thus revised the manuscript on Pages 17, 19 and 22 to tone down our conclusion. Lastly, we think the overly increased abundance in seRNA-1 vs. antisense pull down in Fig. 4a is probably due to stronger protein-interacting ability of seRNA-1 with many other proteins, which however was not seen in seRNA-2 pull down.

3.4. It is quite surprising that both knockdown of hnRNPL and removing of CAAA tract attenuated the expression of neighboring genes of seRNA-1, but simultaneously up-regulating seRNA-1, suggesting that it is unlikely the transcript itself (seRNA-1) important for regulating neighboring genes.

Thanks for the comment. It is indeed intriguing that knockdown of hnRNPL induced a modest increase in seRNA-1 expression. We reason that there may exist a transcriptional repression of hnRNPL on seRNA-1 locus mediated by the region harboring CAAA tract. Upon hnRNPL deletion, seRNA-1 was de-repressed thus up-regulated, which however could not activate Mb induction because of the lacking of binding partner hnRNPL. Alternatively, seRNA-1 interaction with hnRNPL may repress seRNA-1 transcription itself thus loss of the interaction with CAAA deletion in turn stimulates seRNA-1 transcription. This will be consistent with our finding hnRNPL knockdown induces seRNA-1 expression. Nevertheless, the produced mutant seRNA-1 transcript lacks the ability to interact with hnRNPL thus failed to activate the neighboring gene. We have revised the manuscript on Pages 16, 20 and 21 to provide clear interpretation. In addition, as suggested by Reviewer 2 in his/her comment 2.6, we have also generated an additional KO cell in which a part of second exon of seRNA-1 was removed. Compared to

control cells, we did not find reduction in Mb expression or change in hnRNPL binding at Mb locus in this mutant, strengthening the importance of CAAA tract and the interaction between seRNA-1 and hnRNPL is essential in regulating Mb expression. Please see Supplementary Fig. 9c-e.

Minor points:

3.5. It is not appropriate to simply remove transcription units from gene regions when counting eRNAs as eRNAs are often found to be transcribed from intronic and exonic regions.

Thanks for the comment. We agree that eRNAs can be generated from intronic or exonic enhancers. Theoretically, these regions should be also taken into consideration when counting eRNAs. However, when using GRO-seq to define eRNAs, it is hard to distinguish reads from a genuine enhancer or the host genic region if the eRNA is generated from exons or introns. To circumvent this problem, we adopted the method from (Zhao et al., Cell Rep, 2016) to separate GRO-seq transcripts into five distinct, non-overlapping classes and only used the intergenic ones for eRNA analyses.

3.6. It is unclear why the eRNAs detected in GRO-seq experiments in Fig. 1a were predominantly from sense direction.

Thanks for the comment. We apologize for the misleading presentation in Fig. 1a. We did observe extensive divergent transcription of eRNAs in our GRO-seq datasets (Fig. 2d-e). In the original Fig.1a, GRO-seq signals were normalized and plotted in one direction. We have now included two-directional plot in Supplementary Fig. 1e to show the bi-directional transcription.

3.7. The exact number of neighboring genes which behaved in the same direction as eRNAs during the process of differentiation from myoblasts to myotubes should be included.

Thanks for the comment. We have now re-analyzed the RNA-seq datasets and found a total of 423 neighboring genes showing the same trend as eRNAs during muscle differentiation (157 up- and 256 down-regulated respectively). The information can be found on Page 5 of the revised text.

3.8. The specificity of FISH signals observed in Fig. 3b and 3c needs to be confirmed by simultaneously knocking down of eRNA of interest.

Thanks for the comment. The same comment is also raised by Reviewer 2. Please see our answer to his/her comment 2.2. Briefly, we have now repeated the FISH assay using a different method from Arjun Raj group (Shaffer et al., Plos One, 2013), in which hybridization was performed in a short time at a higher probe concentration. We found this method reduced false background signal and greatly improved accuracy. We also included the following controls: 1) no probe control, 2) RNase A treatment control, 3) Malat1 positive control and 4) staining in both MB and MT stages to see whether the signals increased during differentiation. Our new FISH results indicated seRNA-1 and seRNA-2 localize in both nucleus and cytoplasm of C2C12 cells; they both form nuclear foci (Fig. 3b, c and Supplementary Fig. 5c). The percentage of seRNA-1-

positive cells increased from 4% to 74% and, for seRNA-2, from 10% to 48% during the transition from C2C12 MBs to MTs, respectively (Fig. 3b, c).

3.9. The authors need to comment on the fact that both seRNA-1 and seRNA-2 also strongly retrieved other proteins, such as PLEC, VIM and CKAP4.

This is a valid point. It is true PLEC, VIM and CKAP4 are among the top ranked proteins retrieved by both seRNA-1 and seRNA-2. We think it is because these proteins are highly abundant cytoskeleton associated proteins enriched in muscle cells, thus most likely their interactions with seRNA-1 or -2 are non-specific and hard to interpret the gene regulatory function of eRNAs. We chose hnRNPs for downstream validation because of their possible roles in gene regulation and known involvement with lncRNA regulation (Yuan et al., J Biol Chem, 2009; Giraud et al., PNAS, 2014; Atianand et al., Cell, 2016; Huarte et al., Cell, 2010). We have revised the manuscript on Page 14 to clarify on this point.

3.10. It is unclear whether the region responsible for binding with hnRNPL in seRNA-2 also has a CAAA tract. It is also interesting to know whether a CAAA motif is enriched in all eRNAs associated with hnRNPL.

Thanks for the comment. Indeed seRNA-2 has a CACACA canonical hnRNPL motif at the 3'end while seRNA-1 has eight CAAA tracts; This may lead to its relatively weak interaction with hnRNPL in vivo thus no functional synergism detected on regulating seRNA-2 target. As suggested, we also examined whether a CAAA motif is enriched in all eRNAs associated with hnRNPL in CLIP-seq and noticed 113 of 356 eRNAs have CAAA tract (at least two copies).

3.11. It was believed that hnRNPL is involved in Pol II elongation and H3K36me3 deposition by interacting with P-TEFb and KMT3A, respectively. Indeed, Pol II elongation and H3K36me3 deposition were largely affected in the absence of hnRNPL. However, it is unclear whether P-TEFb and KMT3A also exhibited increased binding during the process of myoblasts differentiation into myotubes. Furthermore, the Pol II elongation and H3K36me3 deposition should also be examined on another neighboring gene of seRNA-1, Apol6.

Thanks for the comment. We have now performed ChIP-PCR experiments on both Mb and Apol6 loci and observed an increased binding of CCNT1, CDK9, and KMT3a across Mb loci in MT vs MB, suggesting their functional synergism with hnRNPL in inducing of Mb expression. Instead, their binding across Apol6 locus is not the same: hnRNPL, KMT3a and CDK9 binding was increased, but the overall binding for H3K36me3 and CCNT1 was not changed. The results can be found in Fig. 6c and Supplementary Fig. 9b as well as Page 19 of the text

Reviewer #4:

In their manuscript, Zhao et al used GRO-seq to measure nascent RNA in proliferating and in differentiating mouse C2C12 muscle cells. To identify enhancer RNAs, the GRO-seq results where then overlapped with the catalog of muscle enhancers that the authors have previously identified in the same conditions. Using total and PolyA+ RNA-seq and previously generated ChIP-seq datasets, the authors addressed the issues of transcript abundance, directionality and stability. Based on this initial characterization, the authors decided to narrow their study on

eRNAs produced from super enhancers (seRNAs). Upon further characterization, the authors selected two seRNAs induced during myogenic differentiation (possibly in a MyoD-dependent fashion) for functional studies. seRNA-1 and 2 induction during muscle differentiation was confirmed in vitro using C2C12 and primary cells and in vivo during muscle development and regeneration. Importantly, both seRNAs were showed to be required for the expression of genes located close to the enhancers, as well as for the expression of the myogenic genes MyoD and MyHC and the in vitro differentiation of C2C12 cells. While the authors found that both seRNAs interact specifically with hnRNPL, the protein is important for mediating the activity of only seRNA-1 and only for the expression of the nearby gene Mb. In parallel, the authors reported data indicating that binding to eRNAs could be a general feature of hnRNPL. Based on these results, the authors propose that the eRNA-hnRNPL interaction represents a novel and general mechanism contributing to target mRNA activation.

While intriguing, the manuscript presents a number of critical aspects that need to be addressed to support the authors conclusions.

MAJOR POINTS

4.1. In their previous paper, the authors identified a total of 406 and 759 SEs together with 8389 and 11052 TEs in MB and MT, respectively. How can the number of teRNAs and seRNAs reported in the present manuscript be so much more than the actual number of TEs and SEs? Are the identified eRNAs in large part portions or isoforms of the same (larger) eRNA? Authors must address this discrepancy.

Thanks for the comment. Indeed we have identified a much higher number of teRNAs/seRNAs compared to the number of TEs/SEs. We believe this is mainly because we employed GRO-seq for eRNA definition. As you known GRO-seq detects nascent enhancer transcription allowing us to define the transcription initiation regions and transcription units across the genome de novo. Compared to steady state RNAseq this allowed us to identify all possible transcriptional sites thus a large number of eRNAs. It is not surprising to observe one large enhancer hosts multiple eRNAs, which has been previously shown (Chae et al, BMC Bioinformatics, 2015). We indeed observed isoforms of the same eRNAs but can not possibly assess the degree of isoform formation using GRO-seq data. Please see revised text on Page 5.

4.2. Without something to compare with, it is unclear how meaningful are the results of Figure 1 e-h. How abundant are the transcripts and the enrichments reported compared to known benchmarks? For example, it would be useful to have signals for known protein coding genes and lincRNAs which are known to be differentially expressed during myogenic differentiation as well as for untranscribed gene deserts. This would allow to compare the results reported in Figure 1 to known, previously characterized genomic entities in muscle cells.

Thanks for the comment. We have now re-plotted Figures 1 e-h to include comparison with protein coding gene (PCGs), lncRNA and gene desert regions. In brief, eRNAs are more enriched for transcription factor binding compared to PCGs, lncRNAs or genome random regions (Supplementary Fig. 2e, j). In addition, the Bi-stable and Uni-stable eRNAs are more enriched for chromatin signatures linked to enhancer activity (H3K27ac, H3K4me1) compared

with other types of transcripts; PCGs instead are enriched for promoter mark (H3K4me3) and active transcription elongation marks (H3K79me2 and H3K36me3) (Supplementary Fig. 2b, g). Please see Supplementary Fig. 2a-j for details and Pages 6-7 of the text.

4.3. Figure 1l is low resolution and the corresponding legend uninformative. What is it the area highlighted in yellow? The information provided in this Figure put into question the rationale used to identify SEs as the area indicated by the red bars does not differ much from adjacent areas.

Thanks for the comment. We apologize for unclear marking in Fig. 1l. We have now revised the figure (Fig. 1i) and corresponding figure legend to improve the resolution and clarity. We have removed the yellow mark and included a GRO-seq transcript unit track to indicate the eRNA transcriptional site. The red bar points to the SEs which were defined based on H3K27ac signals through ROSE algorithm (Whyte et al., Cell, 2013). Indeed the H3K27ac signals in the two SEs are much higher than adjacent areas. Other histone marks including H3K4me1, H3K4me2 as comparisons do not show the same trend. We have revised the description on Page 7 to improve the clarity of the result description.

4.4. About half of MB seRNAs of Supplementary Figure 1l is not differentially expressed or it is increased during myogenic differentiation. This raises concern that a large fraction of the seRNAs that the authors describe as more expressed in MB are instead not so. Furthermore, the same MB and MT seRNAs of Figure 1m and Supplementary Figure 1l must be analyzed also during in vitro myogenic differentiation of freshly isolated satellite cells to support findings in primary cells. Also, it would be important to address the question of how many of the identified seRNAs are evolutionarily conserved in human muscle cells. Finally, at least for seRNA 1 and 2, the authors must determine if a human counterpart exists and if these human seRNAs are also required for myogenic differentiation.

Thanks for the comment. We apologize for the confusing result presented in Suppl. Fig. 1l. When examining eRNA expression dynamics in MB and MT using GRO-seq and ChIP-seq (Fig. 1 a, b), only two time points were included i.e. MB as proliferating myoblast (differentiated for 0 hr) and MT as late differentiating myotubes (differentiated for 60 hr for ChIPseq and 48hr for GRO-seq). When performing qRT-PCR in Supplementary Fig. 1l (revised Supplementary Fig. 3a), however, 5 time points (-24, 0, 24, 72 and 120 hrs) were included. Some MB eRNAs interestingly displayed an increase at early differentiation stage but decreased at late stage. If comparing early vs. late stage (-24 vs 72 or 120h), most of them in fact showed a decreased expression. We have now revised the text on Pages 7-8 to make the statement more accurate.

We agree it is better to analyze the expression dynamics of selected seRNAs during in vitro myogenic differentiation of freshly isolated satellite cells. We have now performed the experiment (Supplementary Fig. 3c). Consistent with the results from C2C12 cells, 9 out of 11 MT seRNAs showed increased expression during SC differentiation (72h vs 48h). For MB seRNAs, 7 out of 10 were detectable and indeed 5 showed a decrease in the process. We have now revised the text on Page 8 to describe this result.

We also agree it is necessary to address the seRNA conservation. We have now followed the procedure previously reported to compare the genome coordinates of eRNAs in mouse cells to the human genome (hg19). The eRNAs residing within or overlapping with conserved human

genome regions are considered to be conserved pair-wise with human. As a result, we found that 8.44% eRNAs are conserved; Interestingly, seRNA-1 and seRNA-2 are not on the list. The data were presented in Supplementary Fig. 5b, and Pages 10-11 of the revised text.

4.5. I don't agree that the data of Figure 1 and Supplementary Figure 1 "demonstrate that dynamic SEs are prevalently transcribed in muscle cells". For this, the authors must address the issue of tissue/cells specificity in general for their seRNAs. What percentage of the identified eRNAs are muscle specific? This must be done in general, by comparing the authors NGS data to publicly available data. Also, for the candidates of Figure 1m and Supplementary Figure 1l, this must be done by performing RT-qPCR using RNA extracted from multiple tissues.

Thanks for the critical comment. To address tissue specificity concern, we have now examined eRNAs expression in 18 different cells/tissues including MB, MT, BAT, bone marrow, BMDM, cerebellum, cortex, heart, kidney, liver, lung, MEF, olfactory bulb, placenta, small intestine, spleen, testis, thymus, and found that only a small portion of eRNA are specifically expressed in myoblast or myotube (Supplementary Fig. 3e). However, when compared with protein-coding genes, lncRNAs/eRNAs indeed showed a higher tissue specificity in particular seRNAs showed the highest specificity. In addition, we analyzed seRNAs expression individually in the RNA-seq profiles from 33 mouse tissues and found seRNAs are not restricted to limb tissues (Supplementary Data 2); this was also confirmed by our RT-qPCR detection of RNAs from multiple tissues (Supplementary Fig. 3f). Please also see Page 8 of the revised text.

4.6. There are a couple of caveats with the results of Figure 2 e-l. First, the reported results could be simply secondary to a defect in myogenic differentiation by MyoD KO cells. Authors must show representative pictures and quantify the differentiation index, the fusion index and the number of nuclei per myofiber of C2C12 KO for MyoD compared to controls since this was not reported in their previous paper. Moreover, the authors show that MyoG is enriched at the eRNAs genomic regions and it is expressed similarly to eRNAs. Given that MyoG is directly regulated by MyoD, all the results of Figure 2 e-l could be due not by a direct activity of MyoD, but by MyoG. Authors must address this important mechanistic issue. For example, what is the effect of knocking down MyoG on the expression of the eRNAs?

Thanks for the critical comment. Following your suggestion, we have now further characterized MyoD KO cells indeed showing that MyoD KO cells had defect in myogenic differentiation (Supplementary Fig. 4d), which is consistent with the molecular signature reported in this study (Fig. 2, Supplementary Fig. 4 and Supplementary Data 3) and previous finding from a separate study (Kim et al., Asian-Australas J Anim Sci., 2017). However, we reason the regulation of eRNAs by MyoD is not a simply secondary to the defect in myogenic differentiation by MyoD loss as direct binding of MyoD is seen on their promoters and moreover overexpression of MyoD in 10T1/2 cells led to eRNA induction (Fig. 2h-l). To test if MyoG can regulate eRNAs, we found several seRNAs (seRNA-1, seRNA-2 and seRNA-3) but not all were decreased by MyoG knockdown in C2C12 cells (Supplementary Fig. 4h), suggesting MyoD regulatory roles on seRNAs could not be fully substituted by MyoG. We have also revised the text on Pages 9-10.

4.7. I don't agree that the data of Supplementary Figure 3a support the "extraordinary cell/tissue specificity" of the analyzed seRNAs. Indeed, they are clearly expressed in several tissues (seRNA-1) or similarly expressed in hearth, limb and testis (seRNA-2).

Thanks for the comment. As answered to the above comment 4.5, we agree with your opinion and have revised the statement on Page 8.

4.8. The FISH and RT-qPCR results of Figure 3b-d don't really match. This is particularly true for seRNA-2, which by FISH seems mostly cytoplasmic. Moreover, it is curious that both seRNAs show a perinuclear cytoplasmic signal. Since the authors showed just a single nucleus, it is difficult to tell how general is this behavior. Is this consistently seen in every myonucleus? Does it match with any sub-cellular structure compartment (for example the ER)? RNA-FISH for a control protein coding gene and treatment with RNAse before the RNA-FISH for seRNAs must be provided to support the specificity of the RNA-FISH results.

Thanks for the critical comment. We apologized for the low quality of original FISH images and insufficient controls included to tell the specificity of the signals. The same comment is also raised by Reviewer 2. Please see our answer to his or her comment 2.2. Briefly, we have now repeated the FISH assay using a different method from Arjun Raj group (Shaffer et al., Plos One, 2013), in which hybridization was performed in a short time at a higher probe concentration. We found this method reduced false background signal and greatly improved accuracy. We also included the following controls: 1) no probe control, 2) RNase A treatment control, 3) Malat1 positive control and 4) staining in both MB and MT stages to see whether the signals increased during differentiation. Our new FISH result is different from the original one. First, seRNA-1 and seRNA-2 localize in both nucleus and cytoplasm of C2C12 cells; they both form nuclear foci; a perinuclear cytoplasmic signal was however no longer observed (Fig. 3b, c and Supplementary Fig. 5c). In addition, when quantifying the positively stained cells, the percentage of seRNA-1-positive cells increased from 4% to 74% during the transition from C2C12 MBs to MTs and, for seRNA-2, from 10% to 48%, (Fig. 3b, c). We hope the improved FISH data have answered the reviewer's concerns.

4.9. Since the seRNAs are cytoplasmic, work by binding hnRNPL and are capable to regulate reporters containing the promoter of their targets, they should work in trans. Hence, I have some difficulty in understanding why their overexpression (particularly for seRNA-1 for which the model of Figure 7 implies an in trans interaction with hnRNPL) is ineffective. Important information/controls are missing. Have the seRNAs been overexpressed in myoblasts or myotubes? Are the ectopically expressed seRNAs localized similarly to the endogenous seRNAs? Are the ectopically expressed seRNAs associated to hnRNPL?

Thanks for the excellent comment. First we would like to point out there seems to be some misunderstanding of our reporter data as we didn't have any data showing hnRNPL regulating reporters containing the promoter of their targets. As pointed out in the answer to Comment 4.8, the newly acquired results from FISH and cell fractionation assays now suggest that seRNA-1 enrichment in cytoplasm is in fact low. We think seRNA-1/hnRNPL regulation of its target locus is mainly in cis based on the following lines of evidence: 1) Despite low copy number in cell, it forms clear nuclear foci presumably representing the site of generation or action on target locus; 2) our ChIRP-PCR detects binding of seRNA-1 on its target locus. We have now largely revised the text on Pages 11-12. For over-expression experiment, we performed transfection in myoblast and switched to differentiation medium one day later; the overexpression level was thus detected in myotubes at differentiation Day 2. We have not detected the localization of ectopically expressed seRNAs but suspect they would not be on the target loci. According to prior reports

(Ørom et al, Cell, 2010; Wang et al., Cell Res, 2015), an in trans function of lncRNAs is not likely if ectopically expressed lncRNA does not exert any functional roles.

4.10. The results of Supplementary Figure 3i, j suggest that seRNA-1 and 2 work in trans by affecting the activity of the MyoG and MyHC promoters. Where these genomic regions identified in the seRNA-1 ChIRP? Is hnRNPL involved in this activity of seRNA-1 and 2? If not, what is the mechanism through which seRNA-1 and 2 regulate MyoG, MyHC and myogenic differentiation?

Thanks for the critical comments. Myogenic luciferase reporters were commonly used to assess the degree of differentiation (Lu et al., EMBO J, 2013; Zhou et al., Nat Commun., 2015). It was employed to show knockdown of seRNA-1 or seRNA-2 impedes differentiation process. We think it is hard to infer whether the seRNAs act *in trans* on these myogenic promoters. To test further, we have also performed ChIRP-PCR and found no binding of seRNA-1 on MyoG promoter on MyHC promoter (Supplementary Fig. 9a). Therefore, it is currently unclear how seRNA-1 regulates MyoG, MyHC. Its promoting role in myogenic differentiation may stem from its ability to regulate Mb and Apol6 as well as other genes. In the current study, our focus is to dissect how it regulates Mb synergistically with hnRNPL. We have revised text on Pages 13, 19 and 24 to include the above point. Please also see our answer to Reviewer 3's comment 3.2.

4.11. MyoG expression in Fig. 3i, l is barely visible, while it must be strongly induced by myogenic differentiation and (differently from the results of Fig. 3h, k) there is no obvious effect of seRNA-1/2 knockdown on MyoG expression.

Thanks for the comment. We have repeated the WB using the same batch samples by changing the blocking reagent to 3% milk. The improved result can be found in revised Fig. 3i and l.

4.12. The results of Fig. 3o, p are intriguing as they suggest that the seRNAs might have a biological relevance in vivo. If the authors would manage to demonstrate this, their story would be significantly improved. Are mice treated with seRNA-1 or seRNA-2 defective in muscle regeneration? Authors must show histology of muscles treated with BaCl2 plus seRNA-1 or seRNA-2 siRNAs and controls siRNAs and quantify centrally nucleated fibers, muscle fiber cross-sectional area, muscle infiltrate, muscle fibrosis and adipose tissue deposition. A role for seRNA-1 or seRNA-2 in vivo in muscle regeneration will strongly support the biological relevance of the eRNAs.

Thanks for the comment. We agreed the biological relevance of eRNAs will be strongly supported with in vivo data. We have now performed additional in vivo assays to show that injection of siRNAs targeting seRNA-1 after $BaCl_2$ induced injury regeneration slightly reduced the number of newly formed fibres and increased Oil Red O staining, but did not reach statistical confidence level (Supplementary Fig. 6h, i). No difference of Collagen I staining was observed between control and seRNA-1 knockdown group either (Supplementary Fig. 6j). Knockdown of seRNA-2, on the other hand, reduced the number of newly formed fibres, and increased adipose tissue deposition (Supplementary Fig. 6h, i). Our results thus demonstrated both seRNA-1 and seRNA-2 may be required for muscle regeneration process. Nevertheless, we must point out the siRNA strategy cannot specifically target seRNAs expressed in satellite cells; more solid genetic evidence is needed to confirm seRNA functionality in muscle regeneration in vivo. We have added these results in Supplementary Fig. 6h-j and revised the text on Page 13.

4.13. The fact that hnRNPL has no role in the activity of seRNA-2 raises strong concern regarding the general biological relevance of the interaction of hnRNPL with eRNAs as proposed by the authors. seRNA-2 is important for the regulation of nearby genes, myogenic genes and muscle differentiation but none of this is due to its interaction with hnRNPL. Consequently, there is concern that most of the hnRNPL/eRNA interactions that the authors identified by CLIP could have limited biological relevance. All in all, the authors have supported a role for hnRNPL interaction with eRNAs only for seRNA-1 and this is apparently limited only to the regulation of Mb and Apol6 gene expression, not for the seRNA-1 role in myogenic differentiation.

Thanks for the critical comment. The similar comment has been raised by Reviewers 1, 2 and 3 (comment 1.7, 2,4 and 3.3), promoting us to carefully re-think of the general biological relevance of the interaction of hnRNPL with eRNAs. Although we did observe higher number of CLIP-seq tags in eRNAs are associated with higher expression level of the eRNA neighboring genes (Fig. 5p) and neighboring genes with eRNAs binding are more vulnerable to hnRNPL knockdown (Fig. 5r), implying a potential link between eRNA/ hnRNPL interaction in general. Still, we agree that more solid evidence is needed to extend this model to genome-wide eRNAs spectrum. We have thus revised the manuscript on Pages 17, 19 and 22 to tone down our point.

4.14. Is knockdown of hnRNPL reducing the expression of MyoG and MyHC like the knockdown of seRNA-1? Is knockdown of hnRNPL in C2C12 and in vivo causing myogenic defects similar to those caused by seRNA-1 knockdown? This is key to understand the biological relevance of hnRNPL for the biology of the seRNA and the mechanism proposed by the authors.

Thanks for the critical comment. We agree evidence for biological relevance of hnRNPL will strengthen the synergisms of seRNA-1/hnRNPL action as we proposed. We have now performed the experiments to dissect hnRNPL functions during C2C12 differentiation process. Interestingly, the data (Supplementary Fig. 9h-j) appear to suggest hnRNPL represses muscle differentiation, which is opposite to seRNA-1's pro-myogenic function. We reason that this may be caused by the pleiotropic roles that hnRNPL plays in gene regulation. In addition to regulating seRNA targets at a transcriptional level, it is widely known as a post-transcriptional regulator impacting many mRNAs. We thus believe the functional synergism between seRNA and hnRNPL should be dissected on their target genes using transcription as a read-out while their biological relevancy in regulating myogenic differentiation is limited. We have included the above results and discussion on Pages 20 and 24 of the revised text.

4.15. I don't agree that the fact that regions positive for hnRNPL CLIP tags show higher GRO-seq reads than those with no CLIP signal (Fig. 5f), demonstrate that "hnRNPL binding with eRNAs is a transcriptome-wide event". This is probably simply a reflection that the inefficiency of CLIP, like any IP approach, allows to detect only relatively stable and abundant transcripts, independently of the fact that they belong to enhancer regions.

Thanks for the comment. To resolve this issue, we have now performed correlation analyses between CLIP-seq signals and transcript expression level. As shown in Supplementary Fig. 8c, we did not observe strong association between them, suggesting that hnRNPL binding to RNAs could not be simply explained by their expression level. We have included the above results on

Page 17 of the revised text. Still, as pointed out in the answer to above comment 4.13, we have toned down the generalization of our model to eRNAs transcriptome-wide.

4.16. It is surprising that, despite they used them to identify hnRNPL, the authors did not show CLIP-seq signal for the genomic regions of seRNA-1 and 2 in Figure Fig. 5i. Where seRNA-1 and 2 identified by CLIP-seq? How are the CLIP-seq tags in the seRNA-1 and 2 genomic loci?

Thanks for the comment. We have examined the two loci and interestingly did not detect seRNA-1 among hnRNPL CLIP targets. After careful thinking, we reason this may be caused by intrinsic issue associated with the CLIP-seq data processing pipeline. Briefly, the pipeline eliminated highly repeated reads due to the difficulty in uniquely aligning them to genome; as a result, CAAA repeats contained in a large portion of CLIP raw reads were eliminated (Supplementary Fig. 8f). seRNA-1 was unfortunately eliminated as it possesses eight copies of CAAA repeats. No CLIP tags were detected for seRNA-2 either, probably because the stringent condition used in CLIP procedure disrupted the relatively weaker interaction between seRNA-2 and hnRNPL in vivo. As answered to Reviewer 1's comment 1.7, seRNA-2 only contains one CACACA motif. We have included the above results and discussion on page 18 of the revised text.

4.17. It is unclear if the genes affected by hnRNPL knockdown are enriched for genes regulated during myogenesis and (more importantly) for the same gene categories enriched in the seRNAs knockdown ((Supplementary Figure 1d). Again, the authors must strengthen the link and relevance of the seRNAs-hnRNPL circuit for myogenesis.

Thanks for the comment. As pointed out in the above answer to Comment 4.14, we noticed a repressive role of hnRNPL in C2C12 differentiation. Analyzing RNA-seq revealed that the up-regulated genes upon hnRNPL knockdown are enriched for genes that increase during myogenesis while down-regulated genes are enriched for decreased genes (Fig. 5q, Supplementary Fig. 1f and Supplementary Data 5). We reason that the relevance of seRNA/hnRNPL circuit should be investigated on the transcriptional level using target gene transcription as a read-out.

4.18. I don't agree that the results of Figure 5 "support the notion that hnRNPL-eRNA interaction is critical in regulating target genes expression". seRNA-1 is the only clear example provided by the authors that the interaction between hnRNPL and one eRNA is involved in the regulation of nearby genes. For seRNA-2, despite a specific binding with hnRNPL, there is no role of hnRNPL in the regulation of the seRNA-2 targets. While the authors have provided a genome-wide correlation between hnRNPL-eRNA and gene expression, this remains a correlation with no other clear example (beside seRNA-1) of a direct role of the hnRNPL-eRNA interaction in the regulation of specific targets. Moreover, the authors failed to show that the interaction between hnRNPL and eRNAs is functionally relevant for muscle differentiation.

Thanks for the comment. Please see the answers to Comment 4.13, 4.14, 4.16, 4.17. We have revised the text to tone down our statement on the generality of our model.

4.19. Authors must provide much improved results for the co-IP of Figure 6d, with clearly distinguishable bands and not smeared signals.

We apologize for the poor quality of the IPs. We have now repeated the Co-IP with optimized procedure involving no crosslinking and more suitable for nuclear proteins (Giraud et al, PNAS, 2014). Please see revised Fig. 6d and methods on Page 40.

4.20. The results of Figure 6c, f, g are very difficult to reconcile with those of Figure 1m, 3f and 4f. The level of seRNA-1 increases of nearly 100 folds between MB and MT. The level of hnRNPL enrichment at the Mb locus also increases between MB and MT. Accordingly, knockdown of seRNA-1 or hnRNPL decrease Mb gene expression. If seRNA-1 works by inhibiting hnRNPL recruitment to the Mb locus, then why hnRNPL enrichment on the Mb locus is not higher in MB in which seRNA-1 levels are low? Why hnRNPL enrichment at the Mb locus increases in MT despite a ~100 fold increase in seRNA-1 level? How authors envision the double/discordant activity of seRNA-1 to take place mechanistically? How can this mechanism regulate selectively Mb, given that Apol6 is not affected by seRNA-1 tethering? Can the authors exclude off target effects of seRNA-1 knockdown, hnRNPL overexpression or seRNA-1 tethering? For example, what is the control for Figure 6 j-k? It appears that the authors used untransfected cells or cells transfected with an empty vector. Instead, the appropriate control would be to use a seRNA-1 lacking the hnRNPL binding sequence. This is the best control to support a role for hnRNPL in the activity depicted as opposed to a role of an unknown RNA-binding protein or a generic transcription interference caused by CAS9 blocking access of the transcriptional machinery by steric interference (Cell. 2013 Feb 28; 152(5): 1173–1183). Can the author show that the decreased Mb expression caused by seRNA-1 tethering is specifically due to direct hnRNPL recruitment to the Mb locus by seRNA-1? Why this complex mechanism would be in place to regulate selectively Mb?

Thanks for the comment. Your comment together with the other two reviewers' (Reviewer 1 comment 1.8 and Reviewer 2 comment 2.5) has driven us to explore deeper into the mechanism of how seRNA-1/hnRNPL interaction regulates Mb expression. First, from the collective data we have so far we believe that in myotube the hnRNPL dosage on the target promoter is critical to induce gene expression and seRNA-1 binding with hnRNPL functions to prevent the overloading of hnRNPL. Therefore, loss of seRNA-1 binding by knockdown or removing CAAA led to aberrant enrichment of hnRNPL on the target locus and inhibits the gene activation (Figs. 3f, 4k, 6f, 6g), which can be phenocopied by overexpressing hnRNPL in cells (Fig. 6h). Tethering a seRNA-1 also caused overdose of hnRNPL on the target locus thus similar transcriptional inhibition (Fig. 6i-k). The dosage control is probably executed stochastically through the molecular ratio between seRNA-1 and hnRNPL binding. As determined in Supplementary Fig. 5e, a relatively low number of seRNA-1 molecules were found in MT cells which can bind with a certain number of hnRNPL proteins. More solid evidence on this can be obtained in the future in the next chapter of the study. It is likely the mechanism occurs only in MT not in MB therefore it is hard to infer that hnRNPL enrichment on the Mb locus should be higher in MB in which seRNA-1 levels are low. Second, we believe the transcriptional activating role of hnRNPL is mediated by its interaction with Pol II elongation machinery according to prior reports (Giraud et al., PNAS, 2014; Huang et al., Mol Cell, 2012; Yuan et al., JBC, 2009). Indeed we showed that hnRNPL interacts with CDK9 and CCNT1 (Fig. 6d) and concordant enrichment of all three proteins were seen on the target promoter in myotube (Fig. 6c); interestingly, seRNA-1 knockdown led to increased binding of hnRNPL and CDK9 but decreased CCNT1 binding, suggesting that the complex interaction of hnRNPL with transcriptional machinery needs to be further investigated in the next chapter of the study. Lastly, it remains unclear how hnRNPL is

recruited to the Mb target and how the selectivity is achieved. We believe this may not involve seRNA-1 as loss of seRNA-1 actually led to increased recruitment. It may be mediated by other transcriptional regulators or transcriptional machinery. In fact, the transcriptional functions of post-transcriptional regulators are becoming a focus of intense investigation. For example, RBFox2 broadly interacts with nascent RNA to coordinate transcriptional repression through interacting with PRC2 (Wei et al., Mol Cell, 2016). Due to the limited time given for revision, we feel this can be reserved to the next chapter of the study. However, we are open to any suggestions that the reviewer offers. Please see page 21 of the revised text and our answers' to other reviewers' comments Reviewer 1 comment 1.8 and Reviewer 2 comment 2.5.

As for the control issues mentioned in your comment, we now have added several controls to evaluate the results. For siRNA knockdown experiments, we found that seRNA-1 depletion had no effect on Atp1a1 and Igsf3 and seRNA-2 loss did not decrease Mb or Apol6 either, attesting to the specificity of the seRNA knockdown effect (Supplementary Fig. 5b). We have included the above results and discussion on Page 12 of the revised text. For RNA tethering experiments, we have constructed the mutants with CAAA tract removed. We found tethering seRNA-1 RNA to the seRNA-1 or Mb promoter (Fig. 6i) both led to overdosed amount of hnRNPL deposition on the Mb locus thus the reduced level of Mb transcription (Fig. 6j, k). These effects were however alleviated if the mutant with CAAA deletion was tethered to seRNA-1 or Mb promoter, strengthening the importance of CAAA tract in controlling hnRNPL dosage and Mb expression (Fig. 6j, k). We have included the above results and discussion on Page 20 of the revised text.

MINOR

4.21. The authors did not provided indication of the timepoint/differentiation level of MT used for GRO-seq.

Thanks for the comment. We apologize for unclear information. The C2C12 MT used for GRO-seq was differentiated for two days. We have now provided phase contrast images of the cells to show its differentiation level. Please see revised Supplementary Fig. 1b and Page 5 of the revised text.

Reviewers' comments:

Reviewer #1 (Remarks to the Author):

The authors have satisfactorily addressed all my concerns.

Reviewer #2 (Remarks to the Author):

The authors have comprehensively addressed all my concerns. I feel that this revised manuscript is acceptable for publication in Nature Communication.

Just one minor error to be corrected: On page 15,"we next sought … while seRNA-1 itself was up-regulated (Supplementary Fig. 4e)". The figure citation should be Supplementary Fig. 7f.

Reviewer #3 (Remarks to the Author):

The authors have adequately addressed all my concerns.

Reviewer #4 (Remarks to the Author):

In their revised manuscript, Zhao et al provide some useful control and address some of the initial concern. Nevertheless, the new data fail to solve and actually exacerbates the most important issues as discussed below.

1. As showed by the authors, Mb or Apol6 expression cannot rescue the myogenic defect of cells expressing shRNAs for seRNA-1. This indicates that the regulation of Mb and Apol6 gene expression is irrelevant for the ability of seRNA-1 to stimulate myogenesis. Moreover, the new data by the authors indicate that seRNA-1 does not regulate MyoG or MyHC and that their reduced expression in cells expressing shRNAs for seRNA-1 is simply secondary to a reduced myogenic differentiation of the cells. Finally, as pointed out by the authors, the ability of seRNA-1 to regulate myogenesis "may not even involve hnRNPL". Indeed, the authors have shown that hnRNPL has a biological activity opposite to that of seRNA-1 as it represses myogenesis. Hence, the authors did not provided any insight regarding the molecular mechanism through which seRNA-1 might regulate muscle differentiation. In addition, the biological and functional relevance of the data concerning seRNA-1 interplay with hnRNPL appears very limited. We might have learned a new mechanism through which Mb expression is regulated, but we have learned nothing regarding the regulation of cellular functions by seRNAs raising concern regarding the significance of the findings reported in the manuscript.

2. Another important issue is that the authors did not add any new result to clarify the mechanism responsible for the model proposed in Figure 7, which remain difficult to reconcile with the available data.

3. Only 14% of neighboring genes show the same trend as eRNAs during muscle differentiation. Hence, the vast majority of the eRNAs does not seem functionally relevant for the expression of their neighboring genes.

4. The fact that seRNA-1 and seRNA-2 are not conserved in humans further undermine the biological and functional relevance of the reported findings.

5. Based on the novel data showing that most seRNAs are ubiquitously expressed, the authors must remove "dynamic SEs are prevalently transcribed in muscle cells" from page 8 and any other sentence

alluding the fact that their seRNAs are muscle-cell enriched.

6. The new RNA-FISH results are still not convincing since, compared to Malat1, the signal for seRNA-1 and seRNA-2 seems non-specific. To conclusively support their findings, the authors must repeat the RNA-FISH comparing control to knockdown cells.

7. The answer to my question 4.16 is unsatisfactory. The authors can reanalyze their CLIP-seq data without eliminating repetitive reads. Also, the authors can check seRNA-1 and seRNA-2 enrichment in the hnRNPL CLIP using quantitative PCR and appropriate controls.

8. The new co-IP results in Figure 6d are unconvincing. Indeed, bands for hnRNPL (CCNT1 IP) and CCNT1 or CDK9 (hnRNPL IP) are weak and with a different pattern comparing to the INPUT.

<u>Point to point response</u>

<u>Editor</u>

We feel that the story needs to focus on solid data about hnRNP L/seRNA-1/Mb gene expression; therefore we would suggest moving some of the data (e.g. CLIP data) to supplementary material. We also agree with reviewer #4 point 2 that fig 7 (seRNA-1 regulating the dose of hnRNPL) is not fully supported by the data so the figure needs to be removed or revised. We would expect the revised manuscript to provide further evidence of RNA-FISH, CLIP analysis, and co-immunoprecipitation & western blotting. Please ensure that the corresponding dot plots are overlaid in the bar charts. Please provide how the statistic results have been derived in the source data file.

Thanks for your kind review of our manuscript and suggestions in the revision. We agree that part of the model remains hypothetical so we have now revised the model and please see revised Figure 7. We have now added further evidence of RNA-FISH, CLIP-seq analysis and CLIP-PCR, Co-IP as suggested by Reviewer #4. Please see revised Supplementary Fig. 5d, Supplementary Fig. 8g, revised Fig. 6d and the responses below to Reviewer #4's comments. In addition, we have now revised all our bar chats in the format with overlaid individual dot plots. For statistical results, we have provided the exact Mean, SD values and p values in the source data and pointed out the exact statistical method used in corresponding figure legends.

<u>Reviewer #2:</u>

The authors have comprehensively addressed all my concerns. I feel that this revised manuscript is acceptable for publication in Nature Communication. Just one minor error to be corrected: On page 15,"we next sought … while seRNA-1 itself was up-regulated (Supplementary Fig. 4e)". The figure citation should be Supplementary Fig. 7f.

We thank the reviewer for pointing out this error. We have now corrected it.

<u>Reviewer #4:</u>

In their revised manuscript, Zhao et al provide some useful control and address some of the initial concern. Nevertheless, the new data fail to solve and actually exacerbates the most important issues as discussed below.

1. As showed by the authors, Mb or Apol6 expression cannot rescue the myogenic defect of cells expressing shRNAs for seRNA-1. This indicates that the regulation of Mb and Apol6 gene expression is irrelevant for the ability of seRNA-1 to stimulate myogenesis. Moreover,

the new data by the authors indicate that seRNA-1 does not regulate MyoG or MyHC and that their reduced expression in cells expressing shRNAs for seRNA-1 is simply secondary to a reduced myogenic differentiation of the cells. Finally, as pointed out by the authors, the ability of seRNA-1 to regulate myogenesis "may not even involve hnRNPL". Indeed, the authors have shown that hnRNPL has a biological activity opposite to that of seRNA-1 as it represses myogenesis. Hence, the authors did not provided any insight regarding the molecular mechanism through which seRNA-1 might regulate muscle differentiation. In addition, the biological and functional relevance of the data concerning seRNA-1 interplay with hnRNPL appears very limited. We might have learned a new mechanism through which Mb expression is regulated, but we have learned nothing regarding the regulation of cellular functions by seRNAs raising concern regarding the significance of the findings reported in the manuscript.

We thank the reviewer for the critical comment. We agree that we have not solved all puzzles related to the study even with the newly added data. We would like to emphasize that our main focus is to unveil a novel mechanism that eRNAs exploit to regulate target gene activation and we have successfully achieved the aim by showing eRNAs can bind with hnRNPL to regulate target promoter activation; the novelty and significance of this mechanism was supported by a few recent works suggesting various RNA-binding proteins may control transcription through harnessing regulatory RNAs (Wei et al., Mol Cell, 2016; Xiao et al., Cell, 2019; Li et al, Nat Rev Genet., 2019). Nonetheless, we acknowledge that the biological significance of this regulatory axis remains to be fully teased out. We agree that it is a bit elusive to show that seRNA-1 and -2 regulation of myogenesis is probably not through Mb or Apol6; as pointed on Page 12-13 of the text, since both Mb and Apol6 proteins may regulate myotube function, an alternative way to dissect the regulation relation may be looking into myotube function, for example, through examining O_2 consumption and mitochondrial respiratory capacity as Mb protein plays essential roles in O_2 storage, PO_2 (oxygen partial pressure) buffering and facilitated O_2 diffusion. Likewise, the biological synergism between seRNA-1 and hnRNPL may be dissected from the above aspect. Alternatively, we propose to remove Fig. 3h-p to Suppl. Fig in order to keep the entire story tightly structured.

2. Another important issue is that the authors did not add any new result to clarify the mechanism responsible for the model proposed in Figure 7, which remain difficult to reconcile with the available data.

Thanks for the critical comment. We agree that part of the model remains hypothetical so we have now revised the model to reflect what our current findings suggest.

3. Only 14% of neighboring genes show the same trend as eRNAs during muscle differentiation. Hence, the vast majority of the eRNAs does not seem functionally relevant for the expression of their neighboring genes.

Thanks for the comment. The most accurate way to assign an enhancer to their target genes is to use the enhance-promoter interaction profile, which is however not available in muscle cells. Another common practice is to assume the proximal neighboring genes as target genes of an enhancer. As pointed out on Page 5 of the text, when performing the expression analysis, all neighboring genes within ± 100 kb region flanking an eRNA genomic locus (Ørom et al, Cell, 2010) were used. Since some of the genes may not be regulated by the eRNA, causing the fact only 14% showed correlation. We predict that the correlation between eRNA and neighbor gene expression will be higher if the authentic target genes of eRNA can be inferred using genome-wide enhancer-promoter interaction profile.

4. The fact that seRNA-1 and seRNA-2 are not conserved in humans further undermine the biological and functional relevance of the reported findings.

Thanks for the comment. As pointed out above, our main focus in this study is to dissect how eRNAs regulate the transcription of target genes and what kind of novel protein partners are involved in the mechanism. Although seRNA-1 and seRNA-2 are not conserved in humans, we believe the mechanism can apply to other eRNAs in human or mouse. To support this, we showed that in Hela cells, hnRNPL can interact with eRNAs genome-widely (Supplementary Fig. 8j-m) and in mESCs we demonstrated it can associate with several seRNA candidates (Supplementary Fig. 8n-p). In line with our findings, multiple studies recently demonstrated that various RNA-binding proteins could control transcription through harnessing regulatory RNAs (Wei et al., Mol Cell, 2016; Xiao et al., Cell, 2019; Li et al, Nat Rev Genet., 2019). Nevertheless, we agree that the study of conserved eRNAs will yield more biological significance which can be investigated in the next chapter of our study.

5. Based on the novel data showing that most seRNAs are ubiquitously expressed, the authors must remove "dynamic SEs are prevalently transcribed in muscle cells" from page 8 and any other sentence alluding the fact that their seRNAs are muscle-cell enriched.

Thanks for the critical comment. We have now corrected this statement on Page 8.

6. The new RNA-FISH results are still not convincing since, compared to Malat1, the signal for seRNA-1 and seRNA-2 seems non-specific. To conclusively support their findings, the authors must repeat the RNA-FISH comparing control to knockdown cells.

Thanks for the comment. First we would like to point out Malat1 is an abundantly expressed lncRNA in C2C12 or any cells; it has a Ct value lower than GAPDH's in RT-qPCR detection.

It is thus very easy to be detected by RNA-FISH whatever approaches used (Chen et al., Cell Discov., 2017 and this study). In contrast, as shown in Supplementary Fig. 5f, the levels of seRNA-1 and seRNA-2 are relatively low thus not possible to obtain strong signal as Malat1's. Nonetheless, we are confident that our RNA-FISH signal is specific; to confirm this, we have now repeated FISH in sh-Control vs sh-seRNA cells as suggested by the reviewer. Indeed, we observed a decrease in the number of foci in sh-seRNA vs sh-Control cells for both seRNA-1 and -2. Please see revised Supplementary Fig. 5d and revised text on page 11.

7. The answer to my question 4.16 is unsatisfactory. The authors can reanalyze their CLIP-seq data without eliminating repetitive reads. Also, the authors can check seRNA-1 and seRNA-2 enrichment in the hnRNPL CLIP using quantitative PCR and appropriate controls.

Thanks for the comment. We have reanalyzed the CLIP-seq data without eliminating the repetitive reads and found seRNA-1 had 313 CLIP reads and seRNA-2 24 reads respectively. To further confirm the association of seRNA-1 and seRNA-2 with hnRNPL, as suggested, we have performed CLIP-PCR and found there is 7.13- and 5.23- fold enrichment for seRNA-1 and seRNA-2 respectively when compared to IgG control. For controls, 7SK showed a 17.11 fold enrichment, serving as a positive control (Giraud et al., PNAS, 2014) and no enrichment was observed for seRNA-1 target Mb. Please see revised Supplementary Fig. 8g and revised text on page 18.

8. The new co-IP results in Figure 6d are unconvincing. Indeed, bands for hnRNPL (CCNT1 IP) and CCNT1 or CDK9 (hnRNPL IP) are weak and with a different pattern comparing to the INPUT.

Thanks for the critical comment. We have now repeated the Co-IP experiments. For CCNT1 IP, we increased the input protein and now retrieved much higher amount of hnRNPL. For hnRNPL IP, we repeated with a different antibody (ARP40368_P050, Aviva Systems Biology), which was recently demonstrated to be effective in IP reaction (Xiao et al., Cell, 2019). As shown in revised Fig. 6d, hnRNPL IP efficiency was markedly improved with a significant increase in the retrieved CCNT1 and CDK9 proteins although we still observed different patterns between IP and Input groups; multiple bands were seen in Input lane while only one in IP lane. We speculate this may be due to the selective enrichment for one isoform or protein carrying post-translational modifications. Similar phenomenon was observed in Giraud et al., PNAS, 2014 and Sundararaman et al., Mol Cell, 2016. Please see revised Fig. 6d.

REVIEWERS' COMMENTS:

Reviewer #4 (Remarks to the Author):

The authors have provided stronger data to support their CLIP and co-IP findings, nevertheless there are still issues with specific experiments and the overall novelty/significance of the findings.
For example, the RNA-FISH results are still unconvincing and the authors have not provided any further data to support/explain the molecular mechanism underlying the model of Figure 7.
The authors cited recent work to support the significance of RNA-binding proteins transcriptional control through harnessing regulatory RNAs (Wei et al., Mol Cell, 2016; Xiao et al., Cell, 2019). Nevertheless, it is important to point out that the cited papers describe a major role for RNA-binding proteins in the regulation of PRC2- or YY1-dependent activities and provide strong mechanistic insights on findings.
I maintain that the novelty, biological relevance and mechanistic insight of the authors findings is limited. Sequence-specific hnRNPL binding to lncRNAs to regulate gene expression has been already reported (Cell. 2016 Jun 16;165(7):1672-1685). There is no demonstration that binding to eRNAs is key for genome-wide hnRNPL activity. Most the described eRNAs have not been characterized. SeRNA-1 appears to regulate myogenesis, but the authors offer no mechanistic insight. Even the mechanism through which seRNA-1 regulates Mb gene expression remains in large part hypothetical.

Point to point response

Editor

Your manuscript entitled "MyoD induced enhancer RNA interacts with hnRNPL protein via CAAA motif to activate target gene transcription during myogenic differentiation" has now been seen again by Reviewer #4, whose comments appear below. In light of his/her advice I am delighted to say that we are happy, in principle, to publish a suitably revised version in Nature Communications under the open access CC BY license (Creative Commons Attribution v4.0 International License). We therefore invite you to revise your paper one last time to address the remaining concerns of Reviewer #4. Abstract and the text should be revised to avoid generalizing conclusion from seRNA-1/hnRNPL/Mb to other eRNAs. Regulation of hnRNPL dosage is not fully supported, so please revise sentences to not mention "dosage" regulation. The main manuscript text should be shortened to 6,500 words or fewer. At the same time we ask that you edit your manuscript to comply with our format requirements and to maximise the accessibility and therefore the impact of your work.

Thanks for your kind acceptance of our manuscript. We have revised the manuscript to address the remaining concerns of Reviewer #4. Specifically, abstract and text have been revised to avoid generalizing conclusion from seRNA-1/hnRNPL/Mb to other eRNAs. "dosage" was removed from the sentences accordingly. In addition, we have shortened the manuscript to approximately 6500 words; several Supplementary Notes were created to host surplus text. Please see the following point-to-point responses to the Reviewer #4's comment and to the editorial requests.

Reviewer #4:

The authors have provided stronger data to support their CLIP and co-IP findings, nevertheless there are still issues with specific experiments and the overall novelty/significance of the findings. For example, the RNA-FISH results are still unconvincing and the authors have not provided any further data to support/explain the molecular mechanism underlying the model of Figure 7. The authors cited recent work to support the significance of RNA-binding proteins transcriptional control through harnessing regulatory RNAs (Wei et al., Mol Cell, 2016; Xiao et al., Cell, 2019). Nevertheless, it is important to point out that the cited papers describe a major role for RNA-binding proteins in the regulation of PRC2- or YY1-dependent activities and provide strong mechanistic insights on findings. I maintain that the novelty, biological relevance and mechanistic insight of the authors findings is limited. Sequence-specific hnRNPL binding to lncRNAs to regulate gene expression has been already reported (Cell. 2016 Jun 16;165(7):1672-1685). There is no demonstration that binding to eRNAs is key for genome-wide hnRNPL activity. Most the described eRNAs have not been characterized.

SeRNA-1 appears to regulate myogenesis, but the authors offer no mechanistic insight. Even the mechanism through which seRNA-1 regulates Mb gene expression remains in large part hypothetical.

We thank the reviewer for the critical comment. We agree that we have not solved all puzzles related to the study. In the current version, we have thoroughly revised the text to avoid generalizing conclusion from seRNA-1 to other eRNAs. Despite previous work showing hnRNPL interaction with lncRNAs, we maintain that our study carries sufficient novelty, biological relevance and mechanistic insight as this is the first showing hnRNPL regulation of gene expression through binding with eRNAs. Considering the current ferventness in exploring non-classical functions of RBPs, ours study stands in the frontier of this field thus deserves publication on Nature Communications.
